# The metabolite α-KG induces GSDMC-dependent pyroptosis through death receptor 6-activated caspase-8

**DOI:** 10.1038/s41422-021-00506-9

**Published:** 2021-05-19

**Authors:** Jia-yuan Zhang, Bo Zhou, Ru-yue Sun, Yuan-li Ai, Kang Cheng, Fu-nan Li, Bao-rui Wang, Fan-jian Liu, Zhi-hong Jiang, Wei-jia Wang, Dawang Zhou, Hang-zi Chen, Qiao Wu

**Affiliations:** 1 State Key Laboratory of Cellular Stress Biology, School of Life Sciences, Xiamen University, Xiamen, Fujian China; 2 School of Pharmaceutical Sciences, Xiamen University, Xiamen, Fujian China

**Keywords:** Cell death, Cell signalling, Cancer metabolism, Post-translational modifications

## Abstract

Pyroptosis is a form of regulated cell death mediated by gasdermin family members, among which the function of GSDMC has not been clearly described. Herein, we demonstrate that the metabolite α-ketoglutarate (α-KG) induces pyroptosis through caspase-8-mediated cleavage of GSDMC. Treatment with DM-αKG, a cell-permeable derivative of α-KG, elevates ROS levels, which leads to oxidation of the plasma membrane-localized death receptor DR6. Oxidation of DR6 triggers its endocytosis, and then recruits both pro-caspase-8 and GSDMC to a DR6 receptosome through protein-protein interactions. The DR6 receptosome herein provides a platform for the cleavage of GSDMC by active caspase-8, thereby leading to pyroptosis. Moreover, this α-KG-induced pyroptosis could inhibit tumor growth and metastasis in mouse models. Interestingly, the efficiency of α-KG in inducing pyroptosis relies on an acidic environment in which α-KG is reduced by MDH1 and converted to L-2HG that further boosts ROS levels. Treatment with lactic acid, the end product of glycolysis, builds an improved acidic environment to facilitate more production of L-2HG, which makes the originally pyroptosis-resistant cancer cells more susceptible to α-KG-induced pyroptosis. This study not only illustrates a pyroptotic pathway linked with metabolites but also identifies an unreported principal axis extending from ROS-initiated DR6 endocytosis to caspase-8-mediated cleavage of GSDMC for potential clinical application in tumor therapy.

## Introduction

Cell death is a very complicated and important biological process that contributes to the regulation of various physiological functions and maintenance of body homeostasis. Dysregulation of cell death programming leads to the occurrence of various diseases, including cancers. Therefore, further understanding the functional mechanism and signaling pathways involved in cell death would benefit the treatment of various diseases. Pyroptosis is a form of regulated cell death mediated by gasdermin family proteins.^1^ In humans, the gasdermin family consists of six members: GSDM-A, -B, -C -D, -E (also called DFNA5), and DFNB59. Mice lack GSDMB but express three GSDMAs (mGSDMA1–3) and four GSDMCs (mGSDMC1–4).^2^ Structurally, all gasdermin family members except DFNB59 have an N-terminal pore-forming domain, a C-terminal autoinhibitory domain, and a loop domain that links the N- and C-terminal domains. Protease-mediated cleavage within the linker loop releases the N-terminal domain, which then oligomerizes to form nonselective pores at the plasma membrane and causes membrane permeability changes, cell swelling and membrane rupture. For example, inflammasome-activated caspase-1/4/5/11 cleave GSDMD to produce an N-terminal fragment that triggers pyroptosis,^2–4^ while proapoptotic stimuli activate caspase-3, which cleaves GSDME to induce pyroptosis.^5,6^

The functions of pyroptosis have become a hot spot in cancer research. The Shao group found that GSDME-mediated pyroptosis is associated with the side effects of chemotherapy. Following treatment with chemotherapeutic drugs, activated caspase-3 cleaves GSDME to induce pyroptosis in GSDME-expressing cells. Unfortunately, GSDME is silenced in most tumor cells but is highly expressed in normal cells and tissues, which leads to strong induction of pyroptosis in normal tissues upon the administration of chemotherapeutic drugs.^5^ Therefore, specifically inducing pyroptosis in tumor cells while avoiding normal cell and tissue damage has become a key issue. We previously demonstrated that iron reinforces the effect of ROS-inducing drugs in boosting ROS levels, leading to the activation of GSDME and pyroptosis of melanoma cells. Hence, iron supplementation was used as a sensitizer in combination with clinical drugs to inhibit the growth and metastasis of melanoma in a mouse model, with decreased toxicity.^7^ Although the currently limited research findings cannot fundamentally explain the two outcomes of pyroptosis in normal and tumor tissues, targeting pyroptosis for tumor therapy may be a good strategy, because pyroptosis of tumor cells would not only overcome the apoptosis resistance of tumor cells but also trigger antitumor immunity.^8–11^ However, the specific pyroptosis inducers in tumors are largely unknown.

Cell fate is closely associated with metabolic homeostasis. α-Ketoglutarate (α-KG) is an essential metabolite in the tricarboxylic acid (TCA) cycle and plays a significant role in physiological processes, including lipid biosynthesis, oxidative stress reduction, protein modification, autophagy, and cell death. Several studies have shown that α-KG is a prospective antitumor agent. For example, α-KG suppresses breast cancer oncogenesis by switching metabolism from glycolysis to oxidative phosphorylation.^12^ Accumulated α-KG blocks malignant progression by driving tumor cell differentiation in p53-null tumors.^13^ Moreover, increasing the α-KG/succinate ratio by disrupting mitochondrial complex I suppresses tumor growth.^14,15^ However, the role of α-KG in pyroptosis remains unreported.

The aim of this study was to investigate the molecular mechanism and signaling pathway of α-KG in pyroptotic cell death. We demonstrated that α-KG is promiscuously reduced to another metabolite, L-2HG, by the metabolic enzyme MDH1 in an acidic environment, which then increases ROS levels to induce the oxidation and internalization of the plasma membrane-localized death receptor DR6. Internalized DR6 further recruits both pro-caspase-8 and GSDMC to the DR6 receptosome that provides a platform for the cleavage of GSDMC by active caspase-8, thereby inducing pyroptosis. This pyroptosis was sufficient for the inhibition of tumor growth and metastasis in mouse models. Collectively, this study not only identifies the key node protein (DR6), pyroptosis initiator (caspase-8), and executor (GSDMC) in the α-KG-induced pyroptotic pathway but also provides an important research direction for clinical tumor treatment via pyroptosis induction.

## Results

### α-KG induces pyroptosis through caspase-8-mediated cleavage of GSDMC

**Fig. 1:**
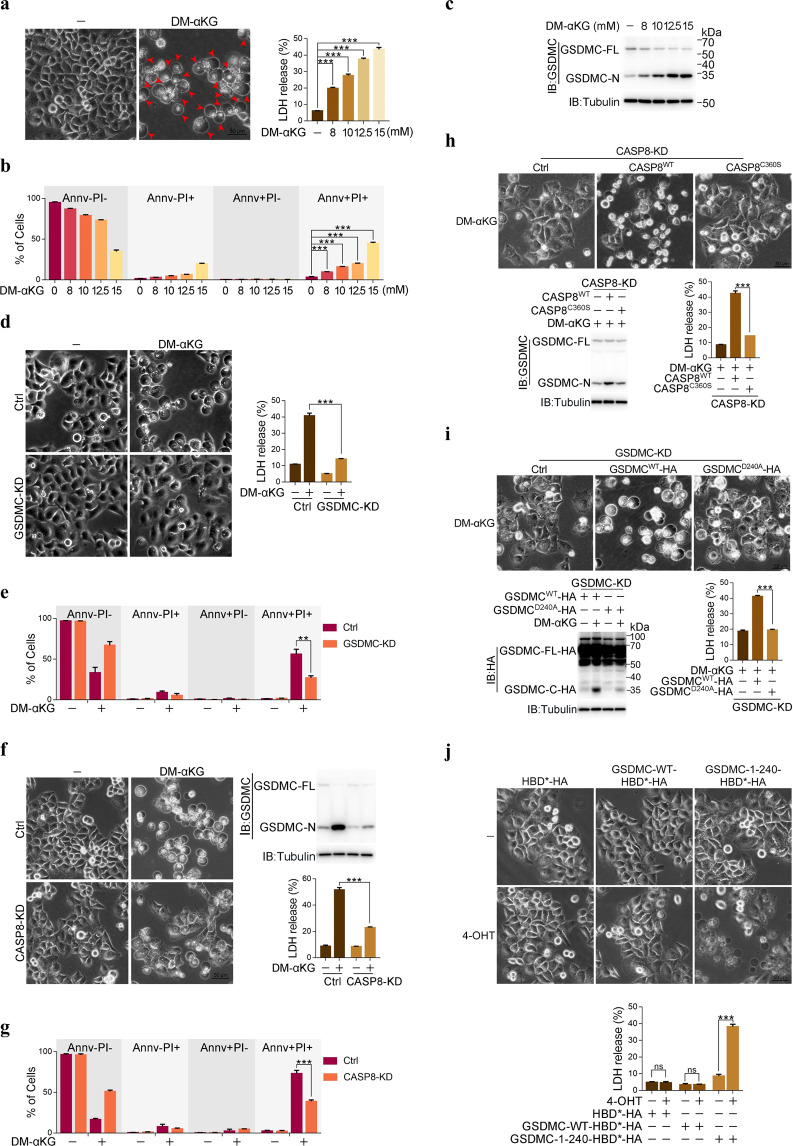
α-KG induces pyroptosis by caspase-8 cleavage of GSDMC. HeLa cells were treated with DM-αKG (15 mM) for 24 h or the indicated times to assess pyroptotic features (including characteristic morphology, GSDMC cleavage, LDH release, and Annv^+^/PI^+^ cells), unless specially defined. **a**–**c** DM-αKG induced pyroptosis in HeLa cells. Pyroptotic morphology and LDH release (**a**), percentage of Annv^+^/PI^+^ cell population (**b**), and GSDMC cleavage (**c**) at different concentrations of DM-αKG as indicated in HeLa cells were shown. Red arrows indicate pyroptotic cells in **a**. Cells were stained with Annexin V (Annv) and propidium iodide (PI), then analyzed by flow cytometry in **b**. The molecular weight is marked on the right in **c**. GSDMC-FL, GSDMC full length; GSDMC-N, GSDMC cleavage at N-terminus. **d**, **e** Effects of GSDMC on DM-αKG-induced pyroptosis (**d**) and Annv^+^/PI^+^ cell population (**e**). GSDMC was knocked down first in HeLa cells, the cells were then treated with DM-αKG. **f**, **g** Effects of caspase-8 on DM-αKG-induced pyroptosis (**f**) and Annv^+^/PI^+^ cell population (**g**). Caspase-8 was knocked down first in HeLa cells, the cells were then treated with DM-αKG. **h** Effect of caspase-8 enzymatic activity on DM-αKG-induced pyroptotic characteristics. Caspase-8 enzymatic dead mutant CASP8^C360S^ was expressed in caspase-8 knockdown (KD) cells. CASP8^WT^ was used as a positive control. **i** Effect of GSDMC^D240A^ on DM-αKG-induced pyroptotic characteristics. siRNA-resistant GSDMC^D240A^ was expressed in GSDMC knockdown HeLa cells. GSDMC^WT^ was used as a positive control. **j** The N-terminus of GSDMC (GSDMC-1–240-HBD*-HA) had an ability to induce pyroptosis. GSDMC-WT-HBD*-HA or GSDMC-1–240-HBD*-HA was transfected into HeLa cells as indicated, the cells were then treated with 4-OHT (3 μM) for 2 h. GSDMC-WT-HBD*-HA was used as a negative control. Tubulin was used to determine the amount of loading proteins. All data are presented as means ± SEM of two or three independent experiments. ***P < 0.001; ns, not significant. The data were analyzed using one-way ANOVA followed by Dunnett’s multiple comparison test (**a**, **b**, **h**, **i**) or two-way ANOVA followed by the Bonferroni test (**d**–**g**, **j**).

The metabolite α-ketoglutarate (α-KG), a key metabolite in the TCA cycle, plays important roles in both metabolic and cellular pathways.^16^ Since exogenously added α-KG cannot pass through the cell membrane, we used dimethyl-α-ketoglutarate (DM-αKG, a cell-permeable analog of α-KG) to treat HeLa cervical carcinoma cells and unexpectedly found that DM-αKG significantly induced cell death with typical morphological features of pyroptosis, i.e., cell swelling and large areas of plasma membrane blebbing (Fig. 1a, left; pyroptotic cells are indicated with red arrows), accompanied by rupture of the cell membrane, as indicated by the release of LDH (Fig. 1a, right) and an increase in the Annv/PI double-positive cell population (Fig. 1b). This DM-αKG-induced pyroptotic morphology was also observed in many other cancer cell lines, including SGC-7901 and BGC-823 human gastric cancer cells, HCT116 human colon cancer cells, Huh7 human hepatoma cells and B16 mouse melanoma cells, although some other cancer cell lines were insensitive to DM-αKG (Supplementary information, Fig. S1a). Some nontumor cell lines, such as LX-2 human hepatic stellate cells, HaCaT human keratinocyte cells, HFL-1 human lung fibroblast cells, and L929 mouse fibroblast cells, were also resistant to DM-αKG (Supplementary information, Fig. S1b). These results indicated that α-KG can induce lytic cell death accompanied by a pyroptotic morphology in many cancer cell lines.

We further sought to confirm whether this α-KG-induced cell death was pyroptosis. Cleaved gasdermin family proteins are the executors of pyroptosis.^17^ Among gasdermin family members, the cleavage of only GSDMC, not GSDMA, GSDMB, GSDMD, or GSDME, was induced by DM-αKG in a dose-dependent manner in HeLa cells (Fig. 1c and Supplementary information, Fig. S1c). Knocking down or knocking out GSDMC substantially attenuated DM-αKG-induced cell death not only in HeLa cells but also in SGC-7901 cells (Fig. 1d, e and Supplementary information, Fig. S1d–f). There are four *Gsdmc* orthologs in mice (*mGSDMC1–4*). Combined knockdown of these GSDMCs in B16 mouse melanoma cells also impaired the induction of cell pyroptosis by DM-αKG (Supplementary information, Fig. S1g). Given that the α-KG-induced pyroptotic morphology and LDH release were not impaired by pretreatment with Z-DEVD (an inhibitor of apoptosis through inhibition of Caspase-3), necrosulfonamide (NSA, an inhibitor of necroptosis through inhibition of MLKL), or ferrostatin-1 (Fer-1, a ferroptosis inhibitor) (Supplementary information, Fig. S1h), it was concluded that α-KG specifically induces GSDMC-dependent pyroptosis.

Treatment with Z-VAD, a pancaspase inhibitor, suppressed DM-αKG-induced GSDMC cleavage, thereby ameliorating the pyroptotic cell morphology and reducing LDH release in HeLa, SGC-7901 and B16 cells (Supplementary information, Fig. S1i, j). These results strongly suggest that caspases might be involved in GSDMC cleavage. To determine the participation of caspase subtypes, different exogenous recombinant caspases were incubated with GSDMC proteins that were isolated from GSDMC-overexpressing human embryonic kidney (HEK293T) cells. The results revealed that GSDMC was effectively cleaved by caspase-8 and -9 in vitro (Supplementary information, Fig. S1k). However, DM-αKG treatment activated only caspase-8 but not caspase-9 in HeLa cells in a time- and dose-dependent manner (Supplementary information, Fig. S1l), suggesting the involvement of caspase-8 but not caspase-9 in α-KG-induced pyroptosis. Indeed, knockdown of caspase-8 but not caspase-9 prevented the initiation of DM-αKG-induced pyroptosis in HeLa cells (Fig. 1f, g and Supplementary information, Fig. S1d, m). The involvement of caspase-8 in DM-αKG-induced pyroptosis was also observed in SGC-7901 and B16 cells (Supplementary information, Fig. S1n). In caspase-8 knockdown HeLa cells, re-expression of wild-type caspase-8 (CASP8^WT^) but not its enzymatically dead mutant (CASP8^C360S^)^18^ promoted DM-αKG-induced GSDMC cleavage and pyroptosis (Fig. 1h and Supplementary information, Fig. S1o). Clearly, active caspase-8 is required for the cleavage of GSDMC and the subsequent pyroptosis in response to α-KG stimulation.

Caspase cleaves target proteins downstream of an Asp residue. Given that the in-gel migration of the N-terminal cleavage product of GSDMC indicated that its molecular weight is approximately 35 kDa (Fig. 1c), the potential Asp site of α-KG-induced caspase-8-mediated cleavage in GSDMC may lie in a sequence of between 200 and 300 amino acid residues. To investigate this possibility, Asp231, Asp232, Asp233, Asp240 and Asp276 in GSDMC were separately mutated to Ala (Supplementary information, Fig. S1p, top; key sites are highlighted in red), and the cleavage of these GSDMC mutants upon DM-αKG stimulation was then evaluated. DM-αKG efficiently induced the cleavage of GSDMC^WT^, GSDMC^D231/232A^, GSDMC^D233A^ and GSDMC^D276A^ but only slightly promoted GSDMC^D240A^ cleavage (Supplementary information, Fig. S1p, bottom). Similarly, the in vitro cleavage assay also revealed that caspase-8-mediated cleavage of GSDMC was clearly impaired when Asp240 was mutated to Ala (Supplementary information, Fig. S1q). Clearly, Asp240 is a critical site for caspase-8 cleavage. As a result of mutation, GSDMC^D240A^ lost its ability to restore DM-αKG-induced pyroptosis in GSDMC knockdown or knockout cells (Fig. 1i and Supplementary information, Fig. S1r). Asp240 is only present in mouse GSDMC4 (Asp233) (Supplementary information, Fig. S1s). To investigate the involvement of mouse GSDMCs, *mGSDMC1–4* were cloned and transfected separately into B16 cells. Only mGSDMC4 could be clearly detected by the anti-GSDMC antibody we used (Supplementary information, Fig. S1t). We thus used anti-Flag antibody to detect the cleavage of Flag-tagged mGSDMCs, and found that DM-αKG could induce the cleavage of mGSDMC4, but not mGSDMC1, mGSDMC2 or mGSDMC3; however, Z-VAD treatment significantly abolished DM-αKG-induced the cleavage of mGSDMC4 (Supplementary information, Fig. S1u). Knockdown of mGSDMC4 alone was sufficient to impair DM-αKG-induced pyroptosis in B16 cells (Supplementary information, Fig. S1v), demonstrating the involvement of mGSDMC4 in DM-αKG-induced pyroptosis. Moreover, when Asp233 in mGSDMC4 was mutated to Ala, both DM-αKG-induced mGSDMC4 cleavage in B16 cells (Supplementary information, Fig. S1w, left) and caspase-8-mediated mGSDMC4 cleavage in the in vitro case (Supplementary information, Fig. S1w, right) were substantially diminished. Collectively, these results indicate that caspase-8 mainly cleaves GSDMC at Asp240 (Asp233 in mouse GSDMC4) in response to αKG treatment.

To further verify the pore-forming activity of GSDMC cleaved by caspase-8, full-length GSDMC or residues 1–240 of GSDMC were fused to a hormone-binding domain (HBD*)-HA tag, which forced dimerization of GSDMC upon treatment with 4-hydroxytamoxifen (4-OHT).^19^ It was obvious that 4-OHT triggered pyroptosis and LDH release in GSDMC-1–240-HBD*-HA-expressing but not GSDMC-WT-HBD*-HA-expressing HeLa cells (Fig. 1j and Supplementary information, Fig. S1x). Therefore, the N-terminal region of GSDMC from amino acids 1 to 240 is sufficient to induce pyroptosis even without α-KG stimulation. Very recently, it was reported that under hypoxic conditions, TNFα induces the cleavage of GSDMC at Asp365 in breast cancer cells.^20^ However, cleavage at this site was not detected upon αKG stimulation, suggesting that the cleavage of GSDMC may vary depending on the stimulus or cell type.

### DR6 responds to α-KG-induced ROS to initiate pyroptosis

**Fig. 2:**
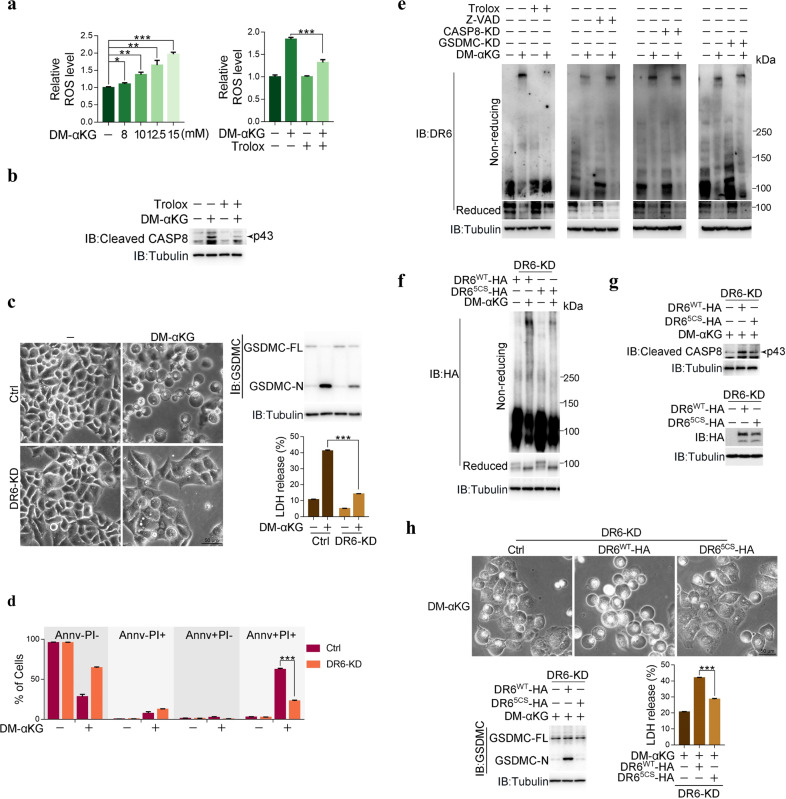
DR6 responds to ROS signals to induce pyroptosis. HeLa cells were treated with DM-αKG (15 mM) for 6 h to determine the ROS level or 24 h to assess the DR6 oxidation, caspase-8 activation, and pyroptotic features (including cell morphology, GSDMC cleavage, LDH release, and Annv^+^/PI^+^ cells), unless specially defined. Inhibitors Trolox (400 μM) and Z-VAD (40 μM) were used to pretreat cells for 2 h. **a** Determination of ROS levels at the indicated concentrations of DM-αKG (left), with or without pretreatment of Trolox (right). **b** Effect of Trolox on DM-αKG-induced activation of caspase-8. Active caspase-8 indicated by arrow was at p43 site. **c**, **d** Effects of DR6 on DM-αKG-induced pyroptotic morphology, GSDMC cleavage, LDH release (**c**) and Annv^+^/PI^+^ cell population (**d**). DR6 was knocked down first in HeLa cells, the cells were then treated with DM-αKG. **e** Effects of Trolox and Z-VAD or caspase-8 and GSDMC on DM-αKG-induced DR6 oxidation. In first two panels, Trolox or Z-VAD was used to pretreat cells. In last two panels, Caspase-8 or GSDMC was knocked down first in cells, the cells were then treated with DM-αKG. DR6 oxidation was observed by running non-reducing gels. **f**﻿–**h** Effects of DR6 oxidation mutant DR6^5CS^ on DM-αKG-induced DR6 oxidation (**f**), caspase-8 activation (**g**) and pyroptotic characteristics (**h**). siRNA-resistant DR6^5CS^ was expressed in DR6 knockdown cells. DR6^WT^ was used as a positive control. The expression levels of DR6^WT^-HA and DR6^5CS^-HA were indicated in **g** (bottom). Tubulin was used to determine the amount of loading proteins. All data are presented as means ± SEM of two or three independent experiments. *P < 0.05, **P < 0.01, ***P < 0.001. The data were analyzed using one-way ANOVA followed by Dunnett’s multiple comparison test (**a** (left), **h**) or two-way ANOVA followed by the Bonferroni test (**a** (right), **c**, **d**).

The mechanism underlying αKG-induced pyroptosis was further studied. Elevation of ROS levels is associated with the induction of pyroptosis.^7^ We found that DM-αKG treatment elevated ROS levels in a dose-dependent manner in HeLa cells (Fig. 2a, left) and that this elevation of ROS levels was required for α-KG-induced pyroptosis, as indicated by the evident impairment of DM-αKG-induced caspase-8 activation and pyroptosis when ROS were scavenged by Trolox, an inhibitor of ROS^21^ (Fig. 2a, right, b and Supplementary information, Fig. S2a, b). Similar results for GSDMC cleavage impaired by Trolox were also obtained in SGC-7901 and B16 cells (Supplementary information, Fig. S2c). Therefore, α-KG induces pyroptosis by activating ROS signaling.

It remains unknown how ROS signals are responded to initiate pyroptosis. The death receptor family members upstream of caspase-8 include TNFR1, FAS, DR3, DR4 (also called trail-r1), DR5 (also called trail-r2), and DR6.^22^ Knocking down TNFR1, FAS, DR3, DR4, or DR5 had no effect on the DM-αKG-induced pyroptotic morphology of cells (Supplementary information, Fig. S2d, e). However, knocking down or knocking out DR6 impaired the manifestation of the DM-αKG-induced pyroptotic morphology, cleavage of GSDMC and rupture of the cell membrane in HeLa, SGC-7901 and B16 cells (Fig. 2c, d and Supplementary information, Fig. S2f, g), indicating the involvement of DR6 in α-KG-induced pyroptosis. The finding that DM-αKG-induced caspase-8 cleavage was attenuated by Trolox (Fig. 2b) suggests a possible link between DR6 and the α-KG-mediated increase in ROS levels. ROS transmit signals by inducing protein oxidation.^23^ In the current context, the DM-αKG-induced increases in ROS levels clearly contributed to DR6 oxidation in both HeLa and SGC-7901 cells, as revealed by the detection of DR6 in the high molecular weight complex by SDS-PAGE under nonreducing conditions (Fig. 2e and Supplementary information, Fig. S2h), which was abolished by pretreatment of cells with the ROS scavenger Trolox or incubation of cell lysates with the reducing agent β-mercaptoethanol (Fig. 2e and Supplementary information, Fig. S2i), indicating clustering of DR6 facilitated by the formation of intermolecular disulfide bonds. Since this high molecular weight complex was not detected in DR6 KO cells (Supplementary information, Fig. S2j), and other well-known ROS inducers (such as H_2_O_2_, antimycin A, oligomycin, NaAsO_2_, and rotenone) barely induced DR6 modification (Supplementary information, Fig. S2k), it is likely that α-KG specifically induces oxidative modification of DR6. Moreover, DR6 oxidation in HeLa cells was not influenced by either knockdown of caspase-8 and GSDMC or treatment with Z-VAD (Fig. 2e). Collectively, these results suggest not only that DR6 is an upstream factor of caspase-8 and GSDMC but also that DR6 may respond to the α-KG-induced increase in ROS signaling to induce pyroptosis.

ROS regulate protein function via oxidation of cysteine (Cys) residues.^23^ DR6 is a transmembrane protein with three separate regions: the extracellular region (N-terminus), the transmembrane region, and the intracellular region (C-terminus).^24^ Five Cys residues are in the C-terminus of DR6 (Supplementary information, Fig. S2l; all Cys residues are marked in red), and 19 Cys residues are in the N-terminal region. Comparison of the oxidation levels in the HA-tagged N- and C-terminus of DR6 indicated that the Cys oxidation level in the C-terminal region (DR6-C) was much higher than that in the N-terminal region (DR6-N) (Supplementary information, Fig. S2m), which suggests that DM-αKG mainly induces oxidation of the C-terminal intracellular domain of DR6. Individual Cys mutations in the C-terminal domain did not impair DM-αKG-induced DR6 oxidation (Supplementary information, Fig. S2n), implying that multiple oxidation sites are involved. Mutation of two, three or four Cys residues slightly impaired DM-αKG-induced DR6 oxidation, but mutation of all five Cys residues in the C-terminus of DR6 (DR6^5CS^) largely blocked DM-αKG-induced DR6 oxidation (Fig. 2f and Supplementary information, Fig. S2o), suggesting that αKG-induced oxidation occurs at these five Cys residues. Oxidation of these Cys residues was critical for DR6-mediated pyroptosis, as transfection of DR6^5CS^ failed to rescue αKG-induced caspase-8 activation and pyroptosis in either DR6 knockdown or DR6 knockout cells (Fig. 2g, h and Supplementary information, Fig. S2p, q). Together, these findings suggest that DR6 responds to α-KG-induced increases in ROS signals through oxidation and thus is a prerequisite for α-KG-induced pyroptosis.

### Oxidation of DR6 leads to its internalization

**Fig. 3:**
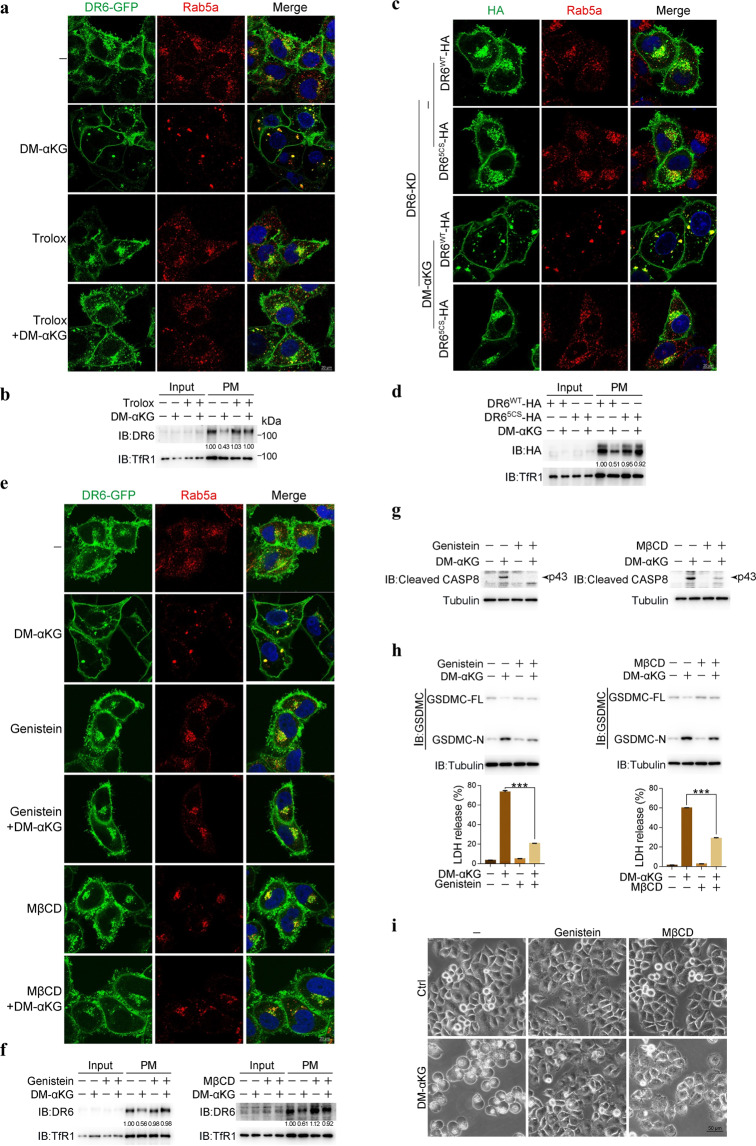
ROS-induced oxidation promotes DR6 endocytosis. HeLa cells were treated with DM-αKG (15 mM) for 6 h to observe protein localization under confocal microscope and analyze protein amounts by fractionation, or 24 h to assess DR6 oxidation level and pyroptotic features (including cell morphology, GSDMC cleavage, and LDH release), unless specially defined. Inhibitors Trolox (400 μM), Genistein (20 μM) or MβCD (1 mM) was used to pretreat cells for 2 h. **a**, **b** Indication of DR6 puncta in cells (**a**) and DR6 expression in plasma membrane (PM) (**b**) in response to DM-αKG stimulation. DR6-GFP was transfected into cells, the cells were then pretreated with Trolox, followed by DM-αKG treatment. Endogenous Rab5a was used as an early endosome marker (**a**). The amounts of DR6 in PM were quantified and shown in **b**. **c**, **d** Effect of DR6 oxidation on DM-αKG-induced DR6 puncta in cells (**c**) and DR6 expression in PM (**d**). siRNA-resistant DR6^WT^ and DR6^5CS^ were expressed in DR6 knockdown cells. **e**, **f** Effects of Genistein and MβCD on DM-αKG-induced DR6 puncta in cells (**e**) and DR6 expression in PM (**f**). DR6-GFP was transfected into cells, the cells were then pretreated with inhibitors, followed by DM-αKG treatment. **g**–**i** Effects of Genistein and MβCD on DM-αKG-induced caspase-8 activation (**g**), GSDMC cleavage and LDH release (**h**), and pyroptotic morphology (**i**). Cells were pretreated with inhibitors as indicated, followed by DM-αKG treatment. Tubulin was used to determine the amount of loading proteins. All data are presented as means ± SEM of two or three independent experiments. ***P < 0.001. The data were analyzed using two-way ANOVA followed by the Bonferroni test.

It was reported that the binding of ligands initiates clustering of death receptors, such as TNFR1 and FAS, followed by internalization of the ligand-receptor complex via clathrin-dependent endocytosis.^22^ Since the α-KG-induced increase in ROS signaling stimulates clustering of DR6 through oxidation, it is possible that α-KG may induce internalization of DR6. Indeed, when HeLa cells were treated with DM-αKG, internalization of DR6 was clearly observed, and the internalized DR6 colocalized with the early endosome marker Rab5a to form cytoplasmic puncta (Fig. 3a), accompanied by a decrease in the DR6 content in the plasma membrane (PM) fraction (Fig. 3b). We herein call these cytoplasmic puncta DR6 receptosomes. Trolox blocked DM-αKG-induced DR6 internalization and puncta formation (Fig. 3a, b). DM-αKG stimulated internalization and puncta formation of only DR6^WT^ and did not have these effects on DR6^5CS^ (Fig. 3c, d). Therefore, after responding to the α-KG-initiated ROS signals, oxidized DR6 in the plasma membrane is internalized into the cytosol to form DR6 receptosomes.

We also sought to determine whether DR6 internalization and puncta formation are required for α-KG-induced pyroptosis. To this end, genistein or MβCD, two endocytic inhibitors, were applied to block DR6 internalization in HeLa cells. Although treatment with genistein or MβCD alone negligibly affected DM-αKG-induced DR6 oxidation (Supplementary information, Fig. S3a), each agent alone clearly blocked DM-αKG-induced DR6 internalization and puncta formation (Fig. 3e, f), activation of caspase-8 (Fig. 3g), cleavage of GSDMC and pyroptosis induction (Fig. 3h, i). Clathrin (CLT)-mediated endocytosis facilitates internalization and recycling of receptors including death receptors, and dynamin (DNM) is the major regulator of clathrin-mediated endocytosis.^25^ Individually knocking down all subtypes of CLT proteins in HeLa cells impaired DM-αKG-induced caspase-8 activation and pyroptosis (Supplementary information, Fig. S3b–e). Similarly, the DM-αKG-induced effects were also blocked by knocking down DNM1 or DNM2 (Supplementary information, Fig. S3b, f–h). This endocytosis-dependent pyroptosis induction was also observed in SGC-7901 and B16 cells (Supplementary information, Fig. S3i). Therefore, α-KG-induced DR6 oxidation and clustering trigger clathrin-mediated endocytosis of DR6 and formation of DR6 receptosomes.

### Internalized DR6 recruits caspase-8 to cleave GSDMC

**Fig. 4:**
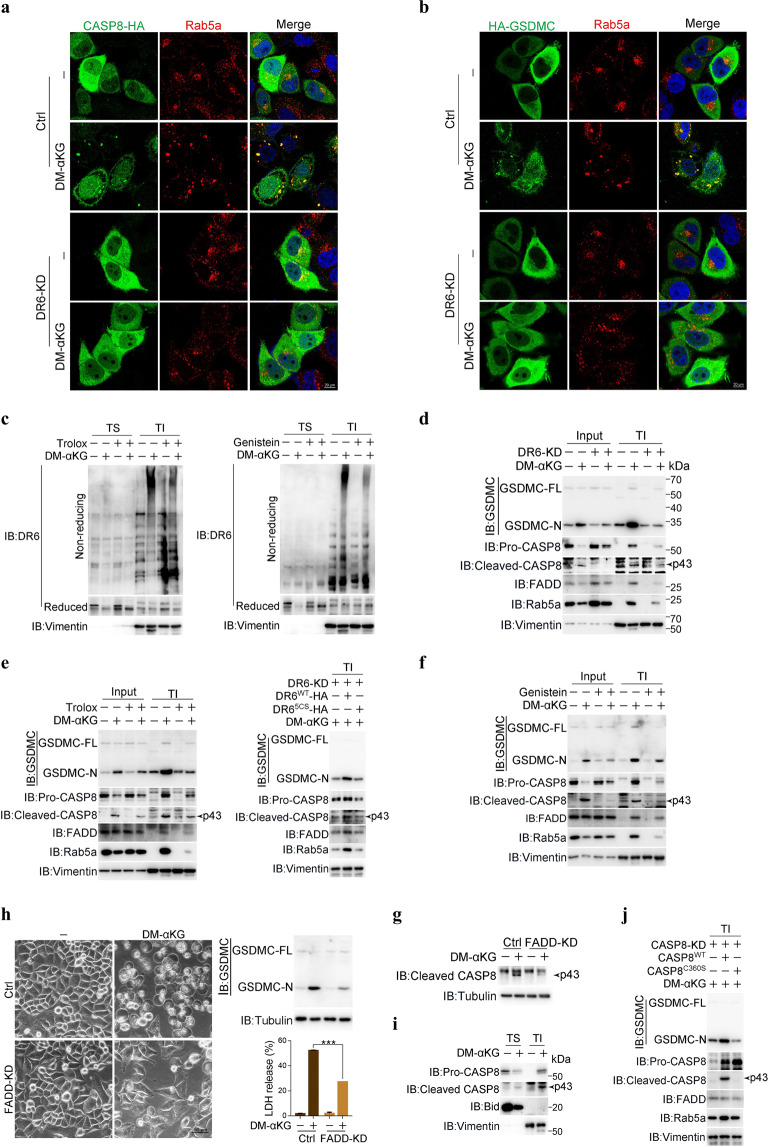
Oxidized DR6 recruits both caspase-8 and GSDMC to DR6 receptosomes. HeLa cells were treated with DM-αKG (15 mM) for 6 h to observe protein localization under confocal microscope, or 24 h to assess DR6 oxidation level and the pyroptotic features (including cell morphology, GSDMC cleavage, and LDH release), unless specially defined. Inhibitors Trolox (400 μM) or Genistein (20 μM) was used to pretreat cells for 2 h. **a**, **b** Effects of DR6 on the localization of Caspase-8 (**a**) and GSDMC (**b**) in the DR6 receptosomes. Caspase-8-HA or HA-GSDMC was transfected into control or DR6 knockdown cells as indicated, the cells were then treated with DM-αKG. Endogenous Rab5a was used as an early endosome marker. **c** Localization of oxidized DR6 in the TI fractions. Cells were treated with DM-αKG, with or without pretreatment of Trolox or Genistein as indicated. TI, the Triton X-100 insoluble fractions. TS, the Triton X-100 soluble fractions. **d**﻿–**f** Analysis of adapter FADD, pro-caspase-8, active caspase-8 and cleaved GSDMC expressions in the TI fractions under different conditions with DM-αKG treatment, including in DR6 knockdown cells (**d**), pretreatment with Trolox (**e**, left), or genistein (**f**), and in DR6^5CS^ expressing cells in which DR6 was knocked down first (**e**, right). DR6^WT^ was used as a positive control. **g**, **h** Effects of adapter FADD on DM-αKG-induced caspase-8 activation (**g**) and pyroptotic features (**h**). FADD was knocked down in cells. **i** Detection of DM-αKG-induced pro-caspase-8, cleaved caspase-8 and Bid levels in TS and TI fractions. **j** Effect of caspase-8 enzymatic activity on DM-αKG-induced expression of FADD, pro-caspase-8, active caspase-8 and GSDMC cleavage in the TI fractions. siRNA-resistant CASP8^C360S^ was expressed in caspase-8 knockdown cells. CASP8^WT^ was used as a positive control. Tubulin was used to determine the amount of loading proteins. All data are presented as means ± SEM of two or three independent experiments. ***P < 0.001. The data were analyzed using two-way ANOVA followed by the Bonferroni test.

Internalized death receptor receptosomes are important for the assembly and activation of caspase-8.^22^ Given that α-KG induces the formation of DR6 receptosomes in HeLa cells, we sought to determine whether caspase-8 is recruited to DR6 receptosomes. Pro-caspase-8, originally dispersed evenly in the cytosol, was translocated to the cytoplasmic puncta and colocalized with either Rab5a or DR6 upon stimulation of cells with DM-αKG, and this translocation of caspase-8 largely depended on the presence of DR6 and DM-αKG-induced ROS elevation, DR6 oxidation and endocytosis (Fig. 4a and Supplementary information, Fig. S4a). Interestingly, DM-αKG also promoted GSDMC translocation to the DR6 receptosome in a manner dependent on DR6, ROS, and endocytosis (Fig. 4b and Supplementary information, Fig. S4b). The findings that knocking down either caspase-8 or GSDMC did not impair the formation of DR6 receptosomes (Supplementary information, Fig. S4c) and that DM-αKG specifically enhanced the interaction of DR6 with caspase-8 and GSDMC (Supplementary information, Fig. S4d) suggest that when DR6 is internalized upon α-KG stimulation, it may recruit pro-caspase-8 and GSDMC to the DR6 receptosome through interaction.

This hypothesis was supported by the results of a cell fractionation assay. It was reported that clustered and internalized death receptosomes are resistant to mild detergents such as Triton X-100.^26^ Indeed, DM-αKG treatment specifically induced the accumulation of the oxidized but not the reduced form of DR6 in the Triton X-100-insoluble (TI) fraction in a ROS- and endocytosis-dependent manner (Fig. 4c), emphasizing the importance of DR6 oxidation in the formation of DR6 receptosomes. Importantly, this α-KG-induced accumulation of oxidized DR6 in the TI fraction was closely associated with the recruitment of caspase-8 and GSDMC. When DR6 was knocked down or ROS-induced oxidation of DR6 was abolished, DM-αKG-induced accumulation of caspase-8 and GSDMC in the TI fraction was greatly diminished (Fig. 4d, e). In addition, inhibition of endocytosis impaired DM-αKG-induced accumulation of caspase-8 and GSDMC in the TI fraction (Fig. 4f). Therefore, the oxidation and subsequent endocytosis of DR6 are prerequisites for the recruitment of caspase-8 and GSDMC to the DR6 receptosome in response to α-KG.

The outcome of caspase-8 and GSDMC accumulation in the receptosome was further investigated. This recruitment of pro-caspase-8 to the receptosome was linked with accumulation of the adapter protein FADD, which led to direct activation of caspase-8 (at 43 kDa) in the receptosome (Fig. 4d–f), consistent with reports that pro-caspase-8 becomes self-activated after recruitment to the receptosome.^27,28^ When FADD, but not another adapter TRADD, was knocked down, DM-αKG no longer activated caspase-8 and induced pyroptosis (Fig. 4g, h and Supplementary information, Fig. S4e, f), indicating FADD-dependent activation of caspase-8.

Notably, active caspase-8 was concentrated in the TI fraction but was not released into the Triton X-100-soluble (TS) fraction upon DM-αKG stimulation (Fig. 4i), leading to the cleavage of GSDMC directly in the TI fraction (Fig. 4d–f) to produce the GSDMC N-terminus for pyroptosis execution in the plasma membrane (Supplementary information, Fig. S4g). However, Bid, a substrate of caspase-8 for apoptosis induction, was not recruited to the TI fraction (Fig. 4i), and its cleavage was scarcely detected upon DM-αKG stimulation (Supplementary information, Fig. S4h). In contrast, treatment with TNFα plus cycloheximide (CHX), a well-known approach for extensive caspase-8 activation, induced not only the cleavage of caspase-8 substrates, such as Bid, caspase-3 and GSDMC, but also the activation of the caspase-3 target GSDME (Supplementary information, Fig. S4h). Therefore, the compartmentalized activation of caspase-8 in DR6 receptosomes ensures the specificity of pyroptotic induction by DM-αKG.

The activation of caspase-8 was indispensable for DM-αKG-induced pyroptosis, because CASP8^C360S^, which was enzymatically dead, did not effectively induce the cleavage of GSDMC in the TI fraction, although it remained in the TI fraction as effective as CASP8^WT^ even in the presence of DM-αKG (Fig. 4j). Together, these results demonstrate that α-KG-induced endocytosis of oxidized DR6 plays a crucial role in the recruitment of both caspase-8 and GSDMC to DR6 receptosomes, where caspase-8 is self-activated and subsequently cleaves GSDMC.

### Physiological role of α-KG in repressing tumor growth and metastasis in mouse models

**Fig. 5:**
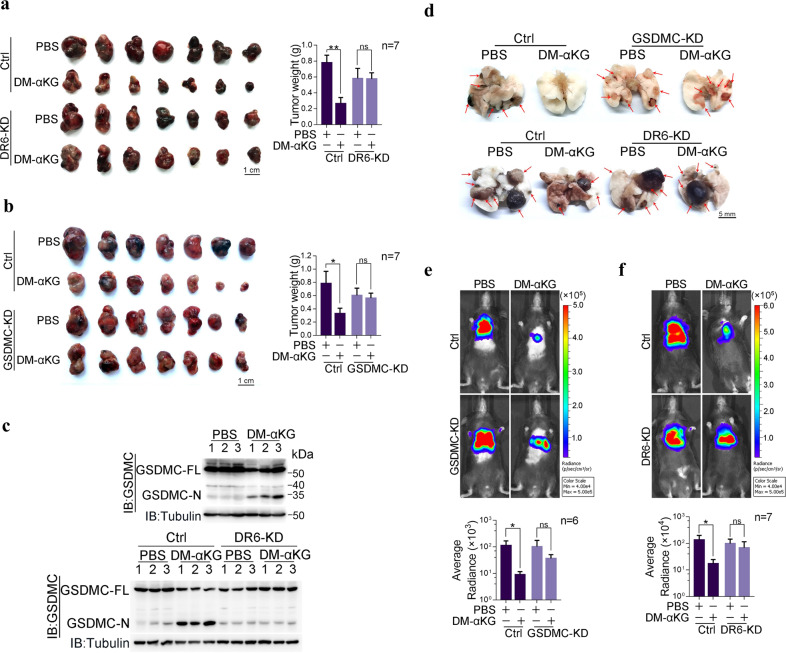
α-KG inhibits tumor growth and metastasis in mouse models. In xenograft model, mice were administered with DM-αKG (500 mg/kg) once per day for one week. In metastasis model, mice were administered with DM-αKG (500 mg/kg) once per day for 30 days. **a**, **b** Indication of xenograft tumors in terms of size and weight. B16 cells with or without knockdown of DR6 (**a**) or GSDMC (**b**) were injected into C57BL/6J mice to form subcutaneous xenografts (*n* = 7). **c** The corresponding expression levels of GSDMC in the tumor samples from **a** were indicated. **d**–**f** Effect of DM-αKG on tumor metastasis. B16 cells with GSDMC (**e**, *n* = 6) or DR6 (**f**, *n* = 7) knockdown were intravenously injected into C57BL/6J mice. Representative images of metastatic tumors in the lung are indicated by red arrows (**d**). The corresponding luciferase signal intensities of the metastatic tumors in the mice are shown (**e** (up), **f** (up)). Tumor metastasis was quantified using bioluminescence imaging (**e** (down), **f** (down)). All data are presented as means ± SEM. *P < 0.05, **P < 0.01; ns, not significant. The data were analyzed using two-way ANOVA followed by the Bonferroni test.

To test the antitumor effect of α-KG in vivo, nude mice bearing HeLa cell-derived xenografts were studied. Intratumoral injection of DM-αKG significantly reduced xenograft tumor growth, as revealed by the decreased weight and size of tumors in this group compared to tumors in the control group (Supplementary information, Fig. S5a). DR6-mediated and GSDMC-executed pyroptosis was required for this inhibitory effect of α-KG, as knockdown of either DR6 or GSDMC in HeLa cells almost abolished the suppressive effect of DM-αKG on tumor growth (Supplementary information, Fig. S5a, b). Upon administration of DM-αKG, DR6-dependent cleavage of GSDMC in xenograft tumor tissues was clearly observed (Supplementary information, Fig. S5c). In DR6 knockdown HeLa cells, re-expression of DR6^WT^ restored the suppressive effects of α-KG on xenograft tumor growth and GSDMC cleavage; however, transfection of DR6^5CS^ failed to do so (Supplementary information, Fig. S5d, e). Similarly, transfection of GSDMC^WT^ but not GSDMC^D240A^ into GSDMC KD HeLa cells enhanced the effects of DM-αKG on reducing tumor sizes and weights (Supplementary information, Fig. S5f). This effect of DM-αKG was also observed in B16 cell-derived xenografts. Administration of DM-αKG evidently suppressed tumor growth in a DR6- and GSDMC-dependent manner (Fig. 5a, b) and was associated with DM-αKG-induced GSDMC cleavage (Fig. 5c). Clearly, α-KG inhibited tumor growth by inducing pyroptosis in a DR6- and GSDMC-dependent manner. Notably, although the body weight, colon and spleen were sensitive to several pyroptosis inducers,^5^ no obvious side effects were observed in these organs in normal mice upon DM-αKG administration (Supplementary information, Fig. S5g–i), suggesting the high tolerance of mice to α-KG treatment.

The suppressive effect of α-KG on the metastasis of melanoma cells was also evaluated. As expected, administration of DM-αKG dramatically inhibited lung metastasis of B16 cells in mice, and this inhibitory effect of α-KG relied on the presence of GSDMC and DR6, as DM-αKG did not suppress lung metastasis of B16 cells with GSDMC or DR6 knockdown (Fig. 5d–f). In conclusion, α-KG functions as an antioncogenic metabolite to inhibit tumor growth and metastasis via a DR6- and GSDMC-mediated pyroptotic pathway.

### The enzyme MDH1 catalyzes the conversion of α-KG to L-2HG to elevate ROS

Collectively, the above results demonstrate a unique α-KG-initiated signal transduction pathway proceeding from the elevation of ROS levels to DR6 endocytosis and then from GSDMC cleavage to pyroptosis induction. However, how the ROS level is elevated by α-KG is still unknown. α-KG can function as a co-substrate for many dioxygenases, such as PHD hydroxylase, the Jmjc-domain family of histone demethylases and the TET family of DNA dioxygenases.^29^ However, pretreatment with dimethyloxalylglycine (DMOG) that could inhibit α-KG-dependent DNA hydroxymethylation and histone demethylation,^30^ did not influence αKG-induced ROS elevation or pyroptosis (Supplementary information, Fig. S6a), excluding the involvement of αKG-dependent demethylation of DNA and histones. As an intermediate metabolite in the TCA cycle, α-KG may be metabolized to succinyl-CoA by the α-ketoglutarate dehydrogenase complex (OGDHC) through oxidative decarboxylation or converted to isocitrate by isocitrate dehydrogenase 1 (IDH1) through reductive carboxylation in the TCA cycle.^31^ Moreover, mutations of IDH1/2 in many tumors lead to the reduction of α-KG to produce D-2HG.^31^ Here, knockdown of IDH1 or IDH2 had no effect on the DM-αKG-induced increases in the ROS level or pyroptosis rate (Supplementary information, Fig. S6b–d). In contrast, knockdown of OGDH not only obviously reinforced DM-αKG-induced ROS production (Supplementary information, Fig. S6e) but also sensitized HeLa cells to DM-αKG-induced pyroptosis (Supplementary information, Fig. S6f–h). Since the OGDH complex catalyzes the main consumption of α-KG, it is likely that the OGDH complex may function as an antidote for α-KG-induced pyroptosis by directly diminishing the amount of available α-KG.

**Fig. 6:**
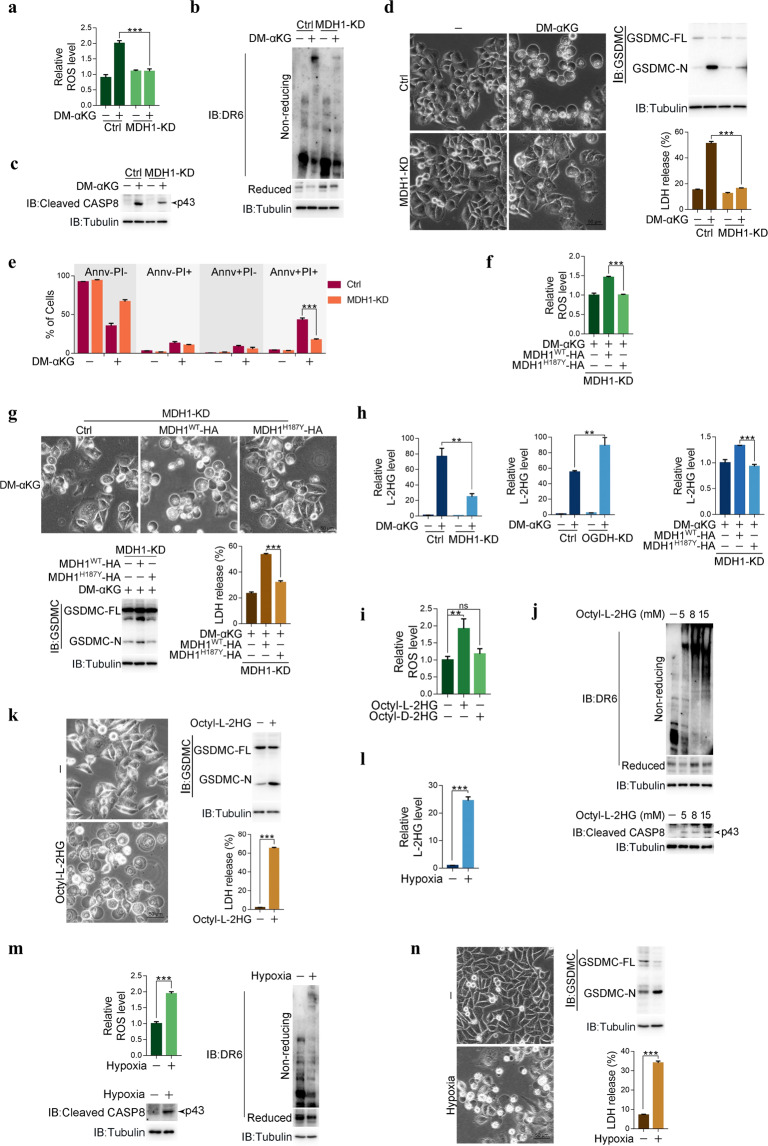
α-KG elevates ROS levels through MDH1-catalyzed conversion to L-2HG. HeLa cells were treated with DM-αKG (15 mM) for 6 h to determine the ROS level and L-2HG level, or 24 h to assess DR6 oxidation, pyroptotic features (including cell morphology, GSDMC cleavage, LDH release, and Annv^+^/PI^+^ cells), unless specially defined. Cells were treated with Octyl-L-2HG (5 mM) or Octyl-D-2HG (5 mM) for 1 h to determine the ROS level, or 2 h to assess DR6 oxidation and pyroptotic features. **a**–**c** Effects of MDH1 on DM-αKG-induced ROS level (**a**), DR6 oxidation (**b**), and caspase-8 activation (**c**). MDH1 was knocked down first in HeLa cells, the cells were then treated with DM-αKG. **d**, **e** Effects of MDH1 on DM-αKG-induced pyroptotic morphology, GSDMC cleavage, LDH release (**d**) and percentage of Annv^+^/PI^+^ cells (**e**). MDH1 was knocked down first in HeLa cells, the cells were then treated with DM-αKG. **f**, **g** Effect of MDH1 enzymatic activity on DM-αKG-induced ROS level (**f**) and pyroptosis (**g**). MDH1 was knocked down in HeLa cells, and siRNA-resistant MDH1^H187Y^ was then transfected into cells. MDH1^WT^ was used as a positive control. **h** Effects of MDH1 (left) and its enzymatic activity (right), or OGDH (middle) on the levels of L-2HG. MDH1 (left) or OGDH (middle) was knocked down in HeLa cells as indicated. In MDH1 knockdown cells, siRNA-resistant MDH1^H187Y^ was expressed, and MDH1^WT^ was used as a positive control (right). **i**﻿–**k** Effects of octyl-L-2HG on ROS level (**i**), DR6 oxidation and caspase-8 activation (**j**) and pyroptosis (**k**). HeLa cells were treated with L-2HG at indicated concentrations (**j**). **l﻿﻿**–**n** Effects of hypoxia on L-2HG level (**l**), ROS level, DR6 oxidation and caspase-8 activation (**m**) and pyroptosis (**n**). Cells were under hypoxia (0.1% O_2_) for 6 h to determine ROS level, 24 h to analyze L-2HG level, and 48 h to assess DR6 oxidation, caspase-8 activation and pyroptotic features. Tubulin was used to determine the amount of loading proteins. All data are presented as means ± SEM of two or three independent experiments. **P < 0.01, ***P < 0.001; ns, not significant. The data were analyzed using two-tailed Student’s *t*-test in **k**–**n**, and one-way ANOVA followed by Dunnett’s multiple comparison test in **f**, **g**, **h** (right), **i** or two-way ANOVA followed by the Bonferroni test in **a**, **d**, **e**, **h** (left and middle).

α-KG can be reduced to L-2HG by LDHA, MDH1 and MDH2 through their promiscuous catalytic activity.^32^ Although knockdown of neither LDHA nor MDH2 affected the DM-αKG-induced increases in the ROS level or pyroptosis rate (Supplementary information, Fig. S6b, i, j), knocking down MDH1 abolished the effects of DM-αKG on the elevation of ROS levels, oxidation of DR6, activation of caspase-8 and pyroptosis in HeLa, SGC-7901 and B16 cells (Fig. 6a–e and Supplementary information, Fig. S6b, k). These effects of MDH1 were closely associated with its catalytic activity. In MDH1 knockdown cells, re-expression of MDH1^WT^ but not its enzymatically dead mutant MDH1^H187Y^ restored the DM-αKG-induced increase in ROS levels and pyroptosis (Fig. 6f, g; Supplementary information, Fig. S6l). These results suggest that the MDH1-catalyzed reduction of α-KG to L-2HG may be involved in pyroptosis induction. Indeed, DM-αKG treatment substantially elevated the intracellular level of L-2HG, which was greatly diminished upon knockdown of MDH1 and was associated with the catalytic activity of MDH1 (Fig. 6h, left and right). In contrast, the intracellular level of L-2HG was elevated upon OGDH knockdown (Fig. 6h, middle). Treatment with octyl-L-2HG (a cell-permeable analog of L-2HG) but not octyl-D-2HG was sufficient to increase ROS levels (Fig. 6i) accompanied by subsequent DR6 oxidation, caspase-8 activation (Fig. 6j) and pyroptosis (Fig. 6k). This effect of octyl-L-2HG on pyroptosis induction was obviously impaired by knockdown of caspase-8, DR6, or GSDMC (Supplementary information, Fig. S6m–o). Based on the well-accepted function of L-2HG in inducing oxidative stress,^33^ L-2HG, a downstream product of α-KG reduction catalyzed by MDH1, relays the propyroptotic effect of α-KG.

The role of L-2HG in pyroptosis induction was further verified under physiological conditions. In line with previous reports,^34,35^ hypoxia (0.1% O_2_) obviously elevated the intracellular level of L-2HG (Fig. 6l). Notably, this increase in L-2HG was closely related to increased ROS levels, DR6 oxidation, caspase-8 activation (Fig. 6m), GSDMC cleavage and pyroptosis (Fig. 6n). When DR6 or GSDMC was knocked out, hypoxia-induced pyroptosis was clearly diminished (Supplementary information, Fig. S6p, q). Hence, the L-2HG-ROS-DR6-GSDMC pathway-related pyroptosis at least partially contributes to cell death under hypoxia stress.

### The glycolysis-produced acidic environment sensitizes cells to α-KG-induced pyroptosis

**Fig. 7:**
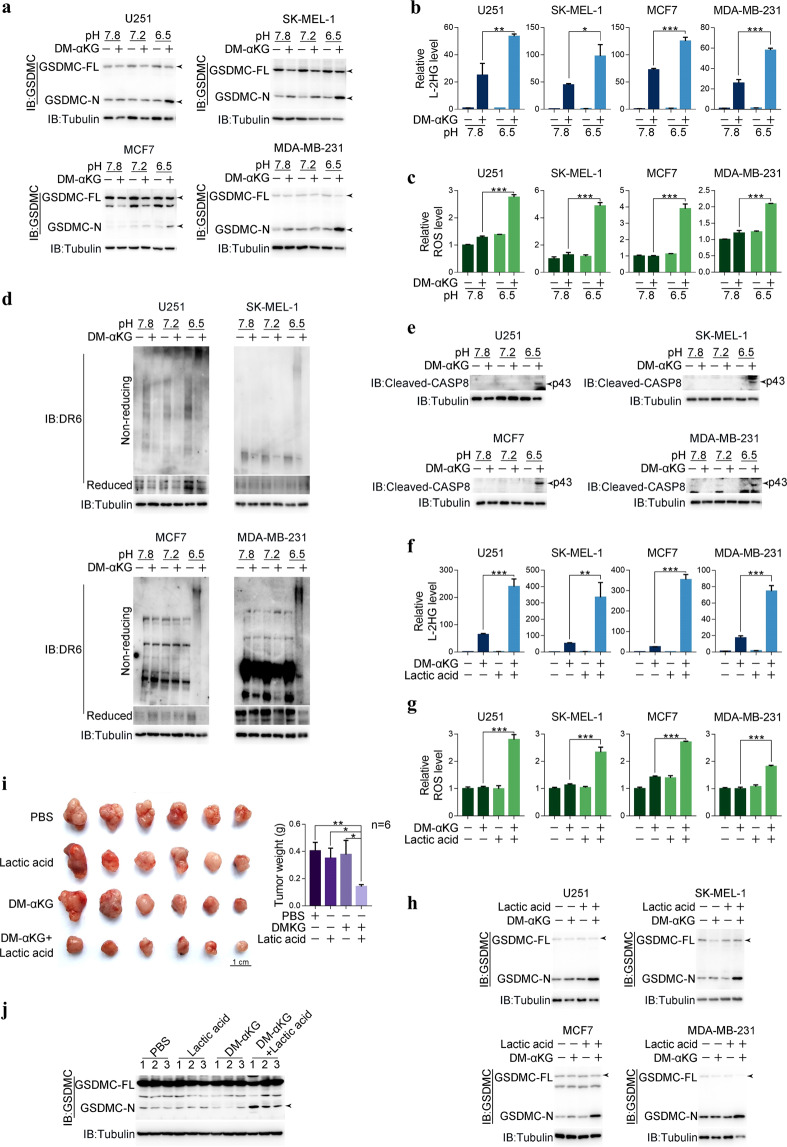
The acidic environment facilitates cell sensitivity to α-KG-induced pyroptosis. Different cancer cell lines, namely, U251, SK-MEL-1, MCF7, and MDA-MB-231 were treated with DM-αKG (15 mM) for 6 h to determine the L-2HG and ROS levels or 24 h to assess DR6 oxidation, caspase-8 activation, and pyroptotic features (including cell morphology, GSDMC cleavage, and LDH release), unless specially defined. **a**﻿–**e** Effects of different pH on DM-αKG-induced GSDMC cleavage (**a**), L-2HG levels (**b**), ROS levels (**c**), DR6 oxidation (**d**), and caspase-8 activation (**e**). Different cancer cells were cultured in medium with different pH values as indicated. **f﻿**–**h** Effects of lactic acid on DM-αKG-induced L-2HG levels (**f**), ROS levels (**g**), and GSDMC cleavage (**h**). Lactic acid (20 mM) together with DM-αKG were used to treat different cancer cells as indicated. **i**, **j** SK-MEL-1 cells were injected into nude mice to form subcutaneous xenografts (*n* = 6). Lactic acid (15 mg/kg) or DM-αKG (500 mg/kg) was intratumorally injected alone or combinedly every other day (four times). The xenograft tumors, tumor weights (**i**, *n* = 6) and GSDMC cleavage (**j**, *n* = 3) were indicated. Tubulin was used to determine the amount of loading proteins. All data are presented as means ± SEM of two or three independent experiments. *P < 0.05, **P < 0.01, ***P < 0.001. The data were analyzed using one-way ANOVA followed by Dunnett’s multiple comparison test in **i** or two-way ANOVA followed by the Bonferroni test in **b**, **c**, **f**, **g**.

We found that different cancer cell lines exhibited distinct responses, including sensitivity and insensitivity, to DM-αKG-induced pyroptosis (Supplementary information, Fig. S1a), and that these responses seemed to be unrelated to the expression of MDH1, DR6, caspase-8, and GSDMC (Supplementary information, Fig. S7a). Since an acidic environment promotes the conversion of α-KG to L-2HG,^32^ it is possible that the intracellular pH may influence the sensitivity of cancer cell lines to α-KG-induced pyroptosis. Indeed, the intracellular pH in the α-KG-sensitive cancer cell lines was much lower than that in the α-KG-insensitive cancer or noncancerous cell lines (Supplementary information, Fig. S7b). When the pH in the culture medium was reduced from 7.8 to 6.5, the originally insensitive cell lines (including the cancer cell lines U251, SK-MEL-1, MCF7 and MDA-MB-231; and the noncancerous cell lines L929, HFL-1, HaCaT, and LX-2) became responsive to DM-αKG-induced pyroptosis (Fig. 7a and Supplementary information, Fig. S7c, d), exhibiting a pyroptotic morphology and GSDMC cleavage, possibly resulting from a significant increase in the L-2HG level (Fig. 7b), ROS level (Fig. 7c), DR6 oxidation (Fig. 7d), and caspase-8 activation (Fig. 7e) under acidic pH conditions in response to DM-αKG. Conversely, elevating the pH in the culture medium led to the resistance of HeLa and B16 cells to DM-αKG-induced pyroptosis (Supplementary information, Fig. S7e). These results consistently demonstrated that an acidic environment sensitizes both normal and cancerous cells to α-KG-induced pyroptosis.

Glycolysis-generated lactic acid is one of the major sources of acidity in tumors.^36^ Treatment with lactic acid obviously lowered the intracellular pH in α-KG-insensitive cell lines (Supplementary information, Fig. S7f), leading to their acquired sensitization to DM-αKG-induced pyroptosis (Supplementary information, Fig. S7g), accompanied by increases in the levels of L-2HG (Fig. 7f) and ROS (Fig. 7g) and cleavage of GSDMC (Fig. 7h). In contrast, when glycolysis in α-KG-sensitive cancer cells was inhibited by phloretin (an inhibitor of glucose transporter 1^37^) or lonidamine (an inhibitor of hexokinase^38^), these cells no longer responded to DM-αKG, as evidenced by their failure to undergo pyroptosis (Supplementary information, Fig. S7h, i), due to the intracellular pH increase (Supplementary information, Fig. S7j) and decrease in the L-2HG level (Supplementary information, Fig. S7k). This effect of lactic acid on sensitizing cells to DM-αKG-induced pyroptosis was further verified in a xenograft model. Intratumoral injection of lactic acid or DM-αKG alone barely influenced the growth of xenografts derived from α-KG-insensitive SK-MEL-1 cells. However, co-injection of lactic acid with DM-αKG significantly decreased tumor sizes and weights (Fig. 7i), accompanied by GSDMC cleavage (Fig. 7j). Clearly, glycolysis-induced acidity in tumor cells and the tumor microenvironment may be a determinant of α-KG-induced pyroptosis.

## Discussion

**Fig. 8:**
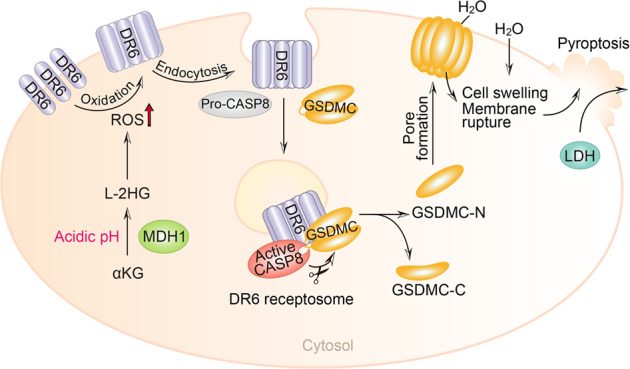
A working model of α-KG-induced pyroptosis. Under acidic environment, the metabolic enzyme MDH1 promiscuously catalyzes the conversion of α-KG to L-2HG, which then boosts ROS level. This signal is responded by the plasma membrane-localized death receptor DR6 by inducing the oxidation of DR6. Oxidized DR6 internalizes into the cytosol, and then recruits both pro-caspase-8 and GSDMC to the DR6 receptosome, in which active caspase-8 cleaves GSDMC, thereby inducing pyroptosis. This story demonstrates a novel pathway from ROS-initiated DR6 endocytosis to caspase-8-cleaved GSDMC for pyroptosis induction.

Accumulating studies have demonstrated that pyroptosis is related to a variety of diseases and conditions, including bacterial infections, chronic inflammation, autoimmune diseases, cardiovascular diseases, neurodegenerative diseases, and tumors. However, the correlations and regulatory mechanisms are largely unknown. Herein, we focused on the functions of the metabolite α-KG in pyroptosis induction and provided several important pieces of evidence: (1) α-KG induces pyroptosis by activating caspase-8 to cleave GSDMC; (2) the α-KG-induced increase in ROS levels initiates the signaling that is responded by the plasma membrane-localized death receptor DR6; (3) ROS-induced oxidation of DR6 promotes its internalization and subsequent formation of the DR6 receptosome; (4) internalized DR6 recruits both pro-caspase-8 and GSDMC to DR6 receptosomes with assistance from the adapter FADD, and the DR6 receptosome provides a platform for the cleavage of GSDMC by active caspase-8; and (5) the acidic environment induces the conversion of α-KG to L-2HG catalyzed by the metabolic enzyme MDH1, which contributes to the increase in ROS levels and then facilitates the sensitization of cancer cells to α-KG-induced pyroptosis (Fig. 8). This study identified an unreported signal transduction pathway for pyroptosis induction via metabolite regulation, a finding that is anticipated to be beneficial for the inhibition of tumor growth and metastasis.

To date, most studies have focused on the activation modes of GSDMD and GSDME, with fewer studies focusing on other members of the gasdermin family. Recently, it was reported that granzyme A from cytotoxic lymphocytes cleaves and activates GSDMB to induce pyroptosis in the targeted cells.^10^ Similar to the observations for GSDMB, GSDMD and GSDME, pyroptosis induction by GSDMC was expected. The function of GSDMC in tumor progression is controversial. On the one hand, GSDMC is highly expressed in colorectal and lung cancers.^39,40^ Silencing GSDMC leads to a significant reduction in the proliferation and tumorigenesis of colorectal cancer cells, suggesting that GSDMC functions as an oncogene.^39^ On the other hand, GSDMC was reported to play a tumor-suppressive role in gastric and esophageal carcinogenesis.^41^ However, in the current study, α-KG-induced and GSDMC cleavage-dependent pyroptosis contributed to the repression of tumor growth and metastasis, suggesting that the cleavage of GSDMC, rather than its expression level, in tumors may be a key target for tumor therapy. More recently, the Hung group reported that hypoxic stress can induce nuclear translocation of PD-L1 to induce the expression of GSDMC with the assistance of STAT3. This PD-L1-induced GSDMC was cleaved by caspase-8 to switch TNFα-induced apoptosis to pyroptosis in breast cancer cells.^42^ Thus, this finding, together with our findings, supports the identity of GSDMC as an executioner of pyroptosis and further fills in the blueprint of pyroptosis, in which an executioner could be activated by diverse upstream stimuli.

Caspase-8 not only functions as either the initiator caspase that activates apoptosis^43^ or an inhibitor of necroptosis through its cleavage of RIP kinases^44,45^ but also is involved in pathogen-induced pyroptosis by cleaving GSDMD.^46,47^ In this regard, connecting its binding and substrate protein cleavage activities, caspase-8 is a functional hub that may determine different types of cell death. In fact, a recent study also demonstrated that caspase-8 functioned as a molecular switch for apoptosis, necroptosis and pyroptosis in mouse models.^48^ During α-KG-induced pyroptosis, caspase-8 cleaves GSDMC at Asp240 to release the pore-forming N-terminal domain. However, the Hung group identified Asp365 in GSDMC as the site of caspase-8 cleavage upon treatment with TNFα/CHX in hypoxia.^42^ Although GSDMC (1–365) was also observed in the in vitro caspase cleavage assays in our study, this fragment was not detected in cells upon DM-α-KG stimulation. Instead, GSDMC (1–240) was clearly observed, and the GSDMC^D240A^ mutant abolished α-KG-induced pyroptosis. This discrepancy not only demonstrates that caspase-8 can cleave GSDMC at multiple sites but also suggests that the exact cleavage site may depend on the stimulus and cell line, although the detailed mechanism underlying this discrepancy remains to be further investigated.

The upstream activators of caspase-8 are death receptors, which are located on the surface of cells, enabling cells to respond to diverse extracellular signals.^22^ The human genome encodes 6 death receptors, all of which except DR6 are activated by the TNF family of cytokines. The extracellular ligand of DR6 appears to be the β-amyloid precursor protein (APP), which is highly expressed in neuronal tissue.^49,50^ This finding explains the functions of DR6 in neuron degeneration and differentiation.^50^ However, DR6 is also expressed in nonneuronal tissue, and the APP-independent activation and function of DR6 have been clearly demonstrated,^51,52^ suggesting the existence of other mechanisms of DR6 activation. Here, DR6 was activated independent of any extracellular ligand; instead, DR6 could respond to an intracellular signal, i.e., ROS induced by α-KG through oxidation. This ROS-activated DR6 is similar to other death receptors in terms of activation and function, including the clustering and internalization of death receptors, the recruitment of adapter proteins, the assembly of receptosomes, and the activation of caspase-8.^22^ Intriguingly, it appears that ROS-activated DR6 specifies the activation of caspase-8 for the induction of pyroptosis but not apoptosis. Although the detailed mechanism by which a cell can distinguish between proapoptotic and propyroptotic signaling through caspase-8 activity is not well understood, we speculate that the compartmentalized pool of active caspase-8 may determine whether the mode of death is apoptosis or pyroptosis. ROS-activated DR6, with assistance from the adapter FADD, recruited pro-caspase-8 to DR6 receptosomes, where caspase-8 was activated through self-cleavage. During this process, active caspase-8 was not released into the cytosol to cleave widespread cytosolic substrates, such as pro-caspase-3 and Bid, which are important for apoptosis. Instead, under this condition, specific recruitment of GSDMC to DR6 receptosomes via their interaction enabled direct GSDMC cleavage by active caspase-8 in the receptosomes, leading to the initiation of pyroptosis but not apoptosis.

Interestingly, our previous study showed that iron/CCCP increases ROS levels to induce oxidative oligomerization of Tom20, leading to GSDME-dependent pyroptosis through the mitochondrial pathway.^7^ However, this study demonstrated that α-KG-induced increases in ROS levels are responded by DR6, resulting in the formation of DR6 receptosomes and GSDMC-dependent pyroptosis. Therefore, different stimuli may induce the same outcome of pyroptosis through different pathways. Although the reasons that DR6 specifically responds to α-KG-mediated ROS increases and Tom20 responds to iron/CCCP-induced ROS increases are currently unknown and deserve further investigation, this pattern could be explained by the idea that different types, concentrations and spatial locations of ROS may determine the specificity of protein oxidation.^53,54^

Although long recognized as a key intermediate metabolite in multiple metabolic pathways, α-KG can also perform nonmetabolic functions by acting as a cosubstrate for α-KG-dependent dioxygenases, including PHD hydroxylase, the Jmjc-domain family of histone demethylases and the TET family of DNA dioxygenases.^29^ In these contexts, α-KG functions as an antitumor metabolite by inducing HIF-1α degradation as well as chromatin and DNA modification.^29^ However, α-KG-dependent dioxygenases are dispensable for pyroptosis induction, as supported by the observation that DMOG, an analog of α-KG that inhibits the activity of α-KG-dependent dioxygenases and the α-KG-dependent DNA hydroxymethylation and histone demethylation,^30^ has no effect on α-KG-induced pyroptosis. Instead, the antitumor role of α-KG in pyroptosis induction is associated with the metabolism of α-KG to L-2HG. Under physiological conditions, 2HG exists in two natural enantiomers, i.e., L-2HG and D-2HG, both of which are structurally similar to α-KG. Thus, both of these enantiomers are considered oncometabolites, principally because of their function as antagonists of α-KG.^55^ However, an antitumor role of 2HG was recently demonstrated. Both L-2HG and D-2HG can inhibit the activity of fat mass and obesity-associated protein, thereby inhibiting the m^6^A-Myc-CEBPA signaling pathway, resulting in the suppression of leukemia and glioma cell proliferation.^56^ A high level of D-2HG suppresses branched chain amino acid transaminase activity, in concert with glutaminase inhibition, led to the increased sensitivity of glioma cells to radiation.^57^ L-2HG can strengthen antitumor immunity by activating CD8^+^ T cells.^58^ In the current study, L-2HG was required for α-KG-induced pyroptosis, because it increased ROS levels, reminiscent of its role in redox homeostasis.^33,59,60^ Although it is still not clear how the L-2HG-mediated increase in ROS levels specifically induces the oxidation of DR6, the function of L-2HG sheds light on its therapeutic potential for tumors through pyroptosis induction.

However, excessive 2HG in plasma causes progressive damage to the brain, which is the main symptom of a disease called 2-hydroxyglutaric aciduria.^61^ Therefore, direct administration of L-2HG for tumor therapy may lead to severe side effects. We demonstrated that administration of α-KG can be an alternative solution because it effectively suppresses tumor growth and metastasis through pyroptosis induction without side effects. α-KG was promiscuously metabolized into L-2HG by the metabolic enzyme MDH1 in an acidic environment, in line with previous reports.^32,62^ Reducing the pH of the culture medium dramatically sensitized originally resistant cells to α-KG-induced pyroptosis by elevating the intracellular level of L-2HG. Because of the glycolytic metabolism in cancer cells, which generates a large amount of lactic acid, an acidic extracellular microenvironment is a major feature of tumor tissue.^36^ Under this condition, α-KG is selectively converted into L-2HG in tumors, thus specifically inducing pyroptosis in tumor cells.

In summary, given the immunogenic nature of pyroptosis, which enhances antitumor immunity,^8–11^ α-KG-based therapy may be another promising strategy, particularly for tumors that rely heavily on glycolysis.

## Materials and methods

### Reagents and antibodies

Chemical reagents DAPI (Cat# D9542), dimethyl α-ketoglutarate (Cat# 349631), ferrostatin-1 (Cat# SML0583), sodium arsenite (Cat# S7400) and trolox (Cat# 238813) were purchased from Sigma Aldrich. Genistein (Cat# HY-14596), rotenone (Cat# HY-B1756), antimycin A (Cat# HY-105755), oligomycin (Cat# HY-16589), methyl-β-cyclodextrin (Cat# HY-101461), Z-DEVD (Cat# HY-12466), DMOG (Cat# HY-15893), phloretin (Cat# HY-N0142) and lonidamine (Cat# AF-1890) were purchased from MedChemExpress. Z-VAD (Cat# A1902) was purchased from ApexBio. TNF-α (Cat# 10602-HNAE) was purchased from Sinobiological. Necrosulfonamide (Cat# T6904) was purchased from TopScience. Cycloheximide (Cat# 2112s) was purchased from Cell Signaling Technology. CM-H2DCFDA (Cat# C6827), an annexin V apoptosis detection kit with FITC (Cat# 88-8005-74), intracellular pH calibration buffer kit (Cat# P35379) and pHrodo™ green AM intracellular pH indicator (Cat# P35373) were purchased from Thermo Fisher Scientific. Plasma membrane protein extraction kit (Cat# ab65400) was purchased from Abcam. FastQuant RT kit (Cat# KR106) was purchased from Tiangen Biotech. CytoTox 96 nonradioactive cytotoxicity assay kit (Cat# G1780) and GoTaq qPCR master mix (Cat# A6101) were purchased from Promega. Lactic acid (Cat# 50-21-5) and hydrogen peroxide (Cat# 7722-84-1) were purchased from Sinopharm Chemical Reagent. Octyl-L-2HG and octyl-D-2HG were synthetized by the Funan Li Group in the School of Pharmacy, Xiamen University.

Recombinant active caspase-3 (Cat# 707-C3-010/CF) was obtained from R&D Systems. Caspase-8 (Cat# ALX-201-062-U025), caspase-7 (Cat# ALX-201-061-U025), caspase-1 (Cat# ALX-201-056-U025) and caspase-9 (Cat# ALX-201-047-U025) were obtained from Enzo Life Sciences.

Goat anti-rabbit (Cat# 31210) and anti-mouse (Cat# 31160) secondary antibodies were purchased from Thermo Fisher Scientific. Anti-tubulin (Cat# T-4026), anti-HA (Cat# H-9658) and anti-Flag (Cat# F-1804) antibodies were purchased from Sigma Aldrich. Anti-Rab5 for confocal microscopy (Cat# 3547S), anti-DR6 (Cat# 93026S), anti-cleaved Caspase-8 (Cat# 9496S), anti-Bid (Cat# 2002 T), anti-Caspase-9 (Cat# 9502S) and anti-LDHA (Cat# 3582T) antibodies were purchased from Cell Signaling Technology. Anti-MDH1 (Cat# 15904-1-AP), anti-Vimentin (Cat# 10366-1-AP), anti-OGDH (Cat# 15212-1-AP), anti-TfR1 (Cat# 10084-2-AP), anti-FADD (Cat# 14906-1-AP) and anti-TRADD (Cat# 15468-1-AP) antibodies were purchased from Proteintech. The anti-GSDMC (Cat# A14550, Lot# 0210740201) antibody was purchased from ABclonal. Anti-caspase-8 (Cat# 551244), anti-active caspase-3 (Cat# 9502S) and anti-Rab5a (Cat# 610724) antibodies were purchased from BD Biosciences. The anti-GSDME (Cat# ab215191) antibody was purchased from Abcam.

### Plasmid construction

Human *GSDMA*, *GSDMB*, *GSDMC*, *GSDMD*, *GSDME*, *Caspase-8*, *DR6*, *MDH1*, *DR5*, *mGSDMC1*, *mGSDMC2*, *mGSDMC3* and *mGSDMC4* cDNA sequences were used as templates. *GSDMA*, *GSDMB*, *GSDMC*, *GSDMC* 1–240, *GSDMD*, *GSDME*, *Caspase-8*, *DR6*, *DR5*, *MDH1*, *mGSDMC1*, *mGSDMC2*, *mGSDMC3* and *mGSDMC4* were separately cloned into a pCDH-CMV-MCS-EF1-Puro vector by using PCR/restriction digest-based cloning. *GSDMC* and *GSDMC* 1–240 were cloned into a pBOB-HBD*-HA vector, which is a gift from Dr. Jiahuai Han (School of Life Science, Xiamen University). siRNA- or sgRNA-resistant mutants were generated by introducing a silent mutation at the siRNA or sgRNA target site. The GSDMC, Caspase-8, MDH1, DR6 and mGSDMC4 mutants were generated by using a QuikChange mutagenesis kit (Stratagene) and verified by sequencing.

### Cell culture and transfection

HEK293T cells and other cell lines, including HeLa, SK-MEL-1, A549, Huh-7, MDA-MB-231, MCF-7, U251, HCT-116, SGC-7901, BGC-823, B16, LX-2, HaCaT, HFL-1, and L929 cells, were cultured in high-glucose Dulbecco’s modified Eagle medium (DMEM) (Sigma) supplemented with 10% fetal bovine serum (FBS), 100 mg/mL streptomycin and 100 IU of penicillin (Bio Basic, Inc.). HFL-1 cells were cultured in F-12 Nutrient Medium supplemented with 10% FBS. During treatment, the concentration of FBS was changed from 10% to 0.5%. The pH of DMEM was adjusted with sodium hydroxide and acetic acid as required. Cell transfection was performed by using TurboFect transfection reagent (Thermo Fisher Scientific, Bremen, Germany), and HEK293T cell transfection was performed by using the calcium phosphate method.

### Generation of the lentiviral system

The lentiviral-based vectors pLKO.1 and pll3.7 were used to express shRNA in cells. The oligonucleotides (Invitrogen) were annealed and subcloned into pLKO.1 or pll3.7 vectors. To produce lentivirus for knocking out different genes, sgRNAs were cloned into lentiGuide-Puro (Addgene 52963). pMD2.G (Addgene 12259) and psPAX2 (Addgene 12260) were used for knocking out lentiviral package. Lentiviruses were generated by transfecting sub-confluent HEK293T cells, which were transfected with the lentiviral vector and packaging plasmids via the calcium phosphate method. 48 h after transfection was initiated, the viral supernatants were collected and used to infect cells. The knockdown efficiency for the target genes was determined by western blotting or RT-PCR analysis. The following oligonucleotide sequences for the pLKO.1 shRNAs were used:

shRNA-caspase-8, 5′-GGAGCAACCCTATTTAGAA-3′;

shRNA-DR3, 5′- CAATCTGGATCCGCCTTA-3′;

shRNA-DR4, 5′-GCTGCTGGTTCCAGCAAAT-3′;

shRNA-DR5, 5′-CAAGGTCGGTGATTGTACA-3′;

shRNA-DR6, 5′-ACGGTTCCTTTATTACCAA-3′;

shRNA-FAS, 5′-CCTCCTACCTCTGGTTCTT-3′;

shRNA-TNFR1, 5′-GTGCCACAAAGGAACCTAC -3′;

shRNA-TRADD, 5′-CCCTTACAGTTTCACTCAT -3′;

shRNA-FADD, 5′-GCAGTCCTCTTATTCCTAA -3′;

shRNA-MDH1, 5′-GCTGTTAGTGTGCATTCTA-3′;

shRNA-MDH2, 5′-CCTTGTGGATGCAATGAAT-3′;

shRNA-OGDH, 5′-GGAACAGATCTTCTGTCAA-3′;

shRNA-CLTA, 5′-GCAAGAAGCAGAGTGGAAA-3′;

shRNA-CLTB, 5′-CCCAGCTATGTGACTTCAA-3′;

shRNA-CLTC, 5′-GCAAGAAAGACAGCCTTTA-3′;

shRNA-DNM1, 5′-GCACTGCAAGGGAAAGAAA-3′;

shRNA-DNM2, 5′-CCTTGACACCATCCTGAAT-3′;

shRNA-mo-DR6, 5′-GGAAGAAAGGGACAGAGAA-3′;

shRNA-mo-GSDMC1-4, 5′-GCATCTTACAGCCAAACTT-3′;

shRNA-mo-GSDMC4, 5′-GCCTCAGTCCTGGATACATTT-3′;

shRNA-mo-caspase-8, 5′-CCTACAGGGTCATGCTCTT-3′;

shRNA-mo-MDH1, 5′- GGAAGTCGGTGTGTATGAA-3′ and

shRNA-Control, 5′-GCGCGCTTTGTAGGATTCG-3′.

The following oligonucleotide sequences for the pll3.7 shRNAs were used:

shRNA-GSDMC, 5′-GGTGCTGAGTGACTTCCAA-3′, and

shRNA-Caspase-9, 5′-GCAAAGTTGTCGAAGCCAA-3′.

The following oligonucleotide sequences for the lentiGuide-Puro sgRNAs were used:

sgRNA-Control, 5′- AAATGTCAGGCCGCGCCGTT 3′;

sgRNA-GSDMC, 5′- GGAGCATCCATGGTCCACAG-3′, and

sgRNA-DR6, 5′- ACCTTTGGGAACATAAGTGG-3′.

### RT-PCR and primers

Total RNA was extracted using a TRIzol kit (Invitrogen), and complementary DNA was synthesized using a reverse transcriptase kit (Tiangen, BJ, China). For the specific quantitative RT-PCR experiments, complementary DNA was used as a template for the amplification, and the level of actin was used as a normalization control. The primer sequences used were as follows (5′-3′):

MDH2: forward, CTACCTCGGACCTGAACAGC;

reverse, CAAACCCGGATTGGCAATGA.

CLTA: forward, GGATACACGGGTAGGGCTTC;

reverse, GACAAAAACCAACCGACCCA.

CLTB: forward, CGGAGAGGAAGTGCGGTC;

reverse, CGACGACGAGAAGAAGCCAA.

CLTC: forward, AAAGAATCTGGGAAAACTCTTCAG;

reverse, TCCGTAACAAGAGCAACCGT.

DNM1: forward, AGCCCGCATTAACCGAATCT;

reverse, GGCCATGTCTGGGGTAAACA.

DNM2: forward, CCCAATCAGGGGGAGATCCT;

reverse, AAGCCCTTCTCCACATCACG.

DR3: forward, GCTGCTGGTTCAGGAATAGG;

reverse, TAAGGCGGATCCAGATTGCT.

DR4: forward, GTGTGGGTTACACCAATGCTT;

reverse, AGTTCCTGGTTTGCACTGACA.

DR5: forward, GCCCCACAACAAAAGAGGTC;

reverse, AGGTCATTCCAGTGAGTGCTA.

FAS: forward, AGATTGTGTGATGAAGGACATGG;

reverse, TGTTGCTGGTGAGTGTGCATT.

TNFR1: forward, AACGAGTGTGTCTCCTGTAGT;

reverse, GGAGTAGAGCTTGGACTTCCAC.

mo-DR6: forward, AGGAGGCGAGTGCTTGA;

reverse, GCCTCCCGGTGCTCTC.

mo-caspase-8: forward, TGAGGCAGACTTTCTGCTGG;

reverse, CTCAGGCTCTGGCAAAGTGA.

Actin: forward, CAGCCTTCCTTCCTGGGCATG;

reverse, ATTGTGCTGGGTGCCAGGGCAG.

GAPDH: forward, CATGTTCGTCATGGGTGTGAACCA;

reverse, AGTGATGGCATGGACTGTGGTCAT

### Immunoprecipitation and western blotting

Immunoprecipitation was performed as described previously.^63^ Briefly, cells were lysed with lysis buffer (50 mM Tris (pH 7.5), 150 mM NaCl, 1 mM EDTA, 1 mM EGTA, 2.5 mM sodium pyrophosphate, 1% Triton X-100, 1 mM PMSF and protease inhibitor cocktail) on ice. Then, the cell lysates were incubated overnight with the appropriate antibody at 4 °C and subsequently with protein G-Sepharose beads for another 1 h at 4 °C. The protein-antibody complexes that were recovered on the beads were collected and washed three times with lysis buffer and then subjected to western blotting.

For the oxidation analysis of DR6, the reduced and non-reduced samples were prepared with or without β-mercaptoethanol. The cells were lysed and collected with 2× SDS loading buffer (0.1 M Tris, 20% glycerol, 4% SDS, and 0.02% bromophenol blue) without β-mercaptoethanol. The samples were separated by sodium dodecyl sulfate-polyacrylamide gel electrophoresis (SDS-PAGE), while non-reduced samples were separated by 4%–12% gradient gel electrophoresis (Sangon Biotech). For other proteins, cells were lysed with lysis buffer (50 mM Tris (pH 7.5), 150 mM NaCl, 1 mM EDTA, 1 mM EGTA, 2.5 mM sodium pyrophosphate, 1% Triton X-100, 1 mM PMSF and protease inhibitor cocktail), and the samples were separated by SDS-PAGE, followed by transfer to polyvinylidene difluoride (PVDF) membranes (Millipore). The membrane was probed with primary antibodies and then incubated with the secondary antibodies. The immune-reactive products were detected by enhanced chemiluminescence (Pierce).

### Assay for GSDMC cleavage by caspases

HEK293T cells were transiently transfected with Flag-tagged GSDMC or its mutants. GSDMC and its mutants were immunoprecipitated with an anti-Flag antibody and protein A/G agarose beads 24 h after initial transfection. Then, after a 3× Flag peptide was used to elute the Flag-GSDMC complexes, 2× caspase reaction buffer (100 mM Hepes (pH 7.2), 100 mM NaCl, 0.2% Chaps, 20 mM EDTA, 10% Glycerol, and 20 mM DTT) at a ratio of 1:1 was mixed with the eluted complexes and recombinant active caspase for 3 h at 37 °C. Caspase-1, -3, -7, -8, and -9 were reconstituted according to the manufacturer’s protocol (Enzo Life Sciences). The final concentrations of caspases were 1 unit for caspase-1, -7, -8, 9 and 7 ng/mL for caspase-3. The cleavage of GSDMC by caspases was detected by western blotting.

### Detection of cell survival rate

Cells were harvested, washed twice with PBS and stained with an annexin V-FITC/PI apoptosis detection kit. The stained cells were analyzed by a BD LRS Fortessa flow cytometer.

### Measuring intracellular pH

The intracellular pH calibration buffer kit was used to measure the intracellular pH of live cells in conjunction with the pHrodo™ green AM intracellular pH indicator (a fluorogenic probe) according to the manufacturer’s protocol (Thermo Fisher Scientific).^64^ Briefly, the cells were washed with live cell imaging solution (LCIS). Ten microlitres of pHrodo™ Green AM was mixed with 100 μL of PowerLoad™ and added to 10 mL of LCIS. The cells were incubated with this mixture for 30 min at 37 °C, followed by pH measurement using a spectrometer. The cells were treated with lactic acid, phloretin or lonidamine, as required, in DMEM without phenol red. The fluorescence intensity of the cells was measured by spectrometer screening to analyze the pH of the cells.

### Extraction of plasma membrane proteins

Plasma membrane proteins and cytosolic proteins were extracted by a plasma membrane protein extraction kit according to the manufacturer’s protocols (Abcam). Briefly, cells were resuspended in a homogenization buffer mix and homogenized by being passed through a 22G syringe 20 times. The supernatants were collected after centrifugation at 700× *g* for 10 min at 4 °C and then centrifuged again at 10,000× *g* for 30 min. The supernatants were collected as the cytosol fraction, and the pellets comprised the total cellular membrane proteins. To obtain plasma membrane proteins, the pellets were resuspended and purified using an upper/lower phase solution. Both plasma membrane proteins and cytosolic proteins were dissolved in 2× SDS loading buffer and then subjected to western blotting.

### Intracellular ROS levels

Cells were incubated with CM-H2DCFDA at a final concentration of 10 μM in FBS-free DMEM for 15 min at 37 °C in the dark and then washed three times with FBS-free DMEM. The cells were collected, and ROS levels were analyzed using an FC500 (Beckman) flow cytometer.

### LDH release assay

Pyroptosis was indicated by detecting the activity of LDH released into cell culture supernatants using a CytoTox 96 nonradioactive cytotoxicity assay kit (Promega) according to the manufacturer’s protocol.

### Microscopy

Cells were seeded in 6-well plates at 40%–60% confluency. The morphology of the pyroptotic cells was captured under a Nikon microscope as phase contrast images. For confocal microscopy, the cells were washed with DMEM and then fixed in 4% paraformaldehyde. The cells were blocked using blocking buffer (3% BSA and 0.2% Triton X-100) and then incubated with the appropriate primary antibody overnight at 4 °C. After washing with washing buffer (0.2% BSA and 0.05% Triton X-100), the cells were incubated with FITC or Texas Red-conjugated secondary antibodies (Life Technologies) for 1 h at 37 °C in the dark. To indicate the nuclei, the cells were stained with 4′,6-diamidino-2-phenylindole (DAPI, 50 μg/mL) for 5 min. Images were captured under a Zeiss LSM 780 confocal microscope.

### Preparation of the TI fraction

Cells were lysed with pre-cold Triton X-100 lysis buffer (50 mM Tris-HCl (pH 7.5), 150 mM NaCl, 1% Triton X-100, 1 mM PMSF and protease cocktail). After incubation for 15 min on ice, the lysates were collected and centrifuged at 15,000× *g* for 30 min at 4 °C to obtain the TI precipitates. The supernatants were collected as soluble fractions. The precipitates were washed 3 times with lysis buffer and with lysis buffer containing 1% SDS at 60 °C in a water bath for 45 min. The precipitates were then sonicated and collected for western blotting analyses. The total proteins were directly collected by Triton X-100 lysis buffer containing 1% SDS.

### LC-MS analysis for L-2HG quantification

For L-2HG detection in different situations, cells (5 × 10^6^) were washed three times with pre-chilled PBS. After adding 1 mL of pre-chilled 80% methanol, the cells were scraped off, and then, after vortexing and centrifugation, the supernatants were collected. Next, the supernatants were dried in a hypothermic vacuum centrifugal concentrator (Labconco Corporation). 50 μL derivatization reagent (diacetyl-L-tartaric anhydride dissolved in 50 mg/mL of acetonitrile: acetic acid (4:1, v/v)) was added to the sample. Next, the sample was derivatized at 70 °C for 2 h and diluted by adding 50 μL of additional derivatization reagent. The quantification of L-2HG was analyzed by liquid chromatography-mass spectrometry (AB SCIEX QTRAP 5500) and normalized to a standard sample.

### Mouse models

Male nude mice (BALB/c, 18–22 g, 7–8 weeks old) and C57BL/6J mice were obtained from the SLAC Laboratory Animal Center, China, and maintained in a 12-h light/dark cycle with free access to food and water. All animal experiments were approved by the Animal Ethics Committee of Xiamen University (acceptance number: XMULAC20120030).

For the xenograft tumor model, 1 × 10^5^ B16 cells, 5 × 10^6^ HeLa cells, DR6 or GSDMC reconstituted HeLa cells were suspended in 100 μL of DMEM and then injected into the anterior flanks of C57BL/6J (B16 cells) or nude mice (HeLa cells). When the tumors developed to the appropriate size, the mice were allocated into two groups for treatment with either vehicle (PBS, 100 μL per mouse) or DM-αKG (500 mg/kg), administered via intratumoural injections once per day for one week (for B16) or every other day for 2 weeks (for HeLa). The mice were sacrificed, and the tumor weights were recorded.

For the insensitive cells treated with DM-αKG, 2 × 10^6^ cells were suspended in 100 μL of DMEM and then injected into the anterior flanks of nude mice. After tumor formation, mice were allocated into four groups for treatment with vehicle (PBS, 100 μL per mouse), DM-αKG (500 mg/kg), lactic acid (15 mg/kg), or both DM-αKG and lactic acid via intratumoural injections every other day for 10 days.

For lung metastasis experiments, B16 cells (2.5 × 10^5^) that expressed luciferase were suspended in 200 μL of PBS and subsequently injected into the lateral tail veins of the mice. The treatments described above were performed by intraperitoneal injections once per day for 30 days. The resulting metastases were detected using an IVIS@ Lumina II system (Caliper Life Sciences, Hopkinton, MA, USA) 10 min after intraperitoneal injection of 3 mg of D-luciferin (15 mg/mL in PBS).

### Statistical analysis

All statistical analyses were performed with GraphPad Prism 6. The data are expressed as means ± SEM. The statistical analysis of two groups was performed using the two-tailed Student’s *t-*test. The differences between multiple groups were analyzed using one-way ANOVA followed by Dunnett’s multiple comparison test or two-way ANOVA followed by the Bonferroni test. The significance thresholds were considered significant (*P* < 0.05), highly significant (*P* < 0.01) or extremely significant (*P* < 0.001).

## Supplementary information

Fig S1

Fig S2

Fig S3

Fig S4

Fig S5

Fig S6

Fig S7
