# Supplementary material for: The metabolite α-KG induces GSDMC-dependent pyroptosis through death receptor 6-activated caspase-8
Source: Cell Res. 2021 May 19;31(9):980–97. doi: 10.1038/s41422-021-00506-9 (PMC8410789; doi:10.1038/s41422-021-00506-9)
Supplement: Supplementary file 1 — Fig S1 [file 41422_2021_506_MOESM1_ESM.pdf]

Supplementary information, Fig. S1. In this figure, different cell lines were treated with DM-αKG (15 mM) for 24 hours to detect pyroptotic features (including cell morphology, GSDMC cleavage, LDH release, and $Annv^+/PI^+$ cells), unless specially indicated otherwise.

(**a**) Different cancer cell lines as indicated were treated with DM-αKG, the cell morphologies were categorized into pyroptosis-sensitive (left) or pyroptosis-insensitive (right) groups.

(**b**) Different normal cell lines as indicated were treated with DM-αKG. The cell morphologies were shown.

(**c**) DM-αKG did not induce cleavage of GSDMA, GSDMB, GSDMD, or GSDME. Different plasmids carrying GSDMA, GSDMB, GSDMD, or GSDME were transfected into HeLa cells.

(**d**) The efficiencies of siRNA-based knockdown (KD) or CRISPR/cas9-based knockout pool (sgRNA) of GSDMC, caspase-8, or caspase-9 in HeLa cells was determined by western blotting.

(e) Knocking out GSDMC rescued cell morphology and decreased LDH release.

(**f-g**) Knocking down GSDMC impaired the DM-αKG-induced pyroptotic morphology or LDH release in SGC-7901 (f) and B16 (g) cells.

(**h**) HeLa cells were pretreated with different agents, including Z-DEVD (40 μM), NSA (5 μM), and Fer-1 (0.5 μM) for 2 hours. The cell morphology and LDH release were detected.

(i) Effect of Z-VAD (40 μM, pretreated with 2 hours) on DM-αKG-induced pyroptotic morphology, GSDMC cleavage, and LDH release in HeLa cells.

(**j**) Effect of Z-VAD on DM-αKG-induced GSDMC cleavage in SGC-7901 (left) and B16 (right) cells.

(k) Determination of caspase subtype in the cleavage of GSDMC. Flag-GSDMC was immunoprecipitated from HEK293T cells that had been transfected with Flag-GSDMC, and then separately incubated with different recombinant caspase proteins (rCASPs) as indicated. The cleavage of GSDMC is indicated with an arrow.

(**l**) DM-αKG induced the activation of Caspase-8, but not Caspase-9, in a time- and dose-dependent manner. HeLa cells were treated with DM-αKG for the indicated times. STS (an apoptotic inductor) was used as a positive control.

(**m**) Knocking down Caspase-9 did not affect the DM-αKG-induced pyroptotic morphology, GSDMC cleavage, or LDH release in HeLa cells.

(**n**) Knocking down Caspase-8 impaired the DM-αKG-induced pyroptotic morphology, GSDMC cleavage, or LDH release in SGC-7901 (top) and B16 (bottom) cells.

(**o**) The expression levels of caspase-8^{WT} and caspase-8^{C360S} in cells. Endogenous caspase-8 was knocked down first in the HeLa cells, and then, caspase-8^{WT} and caspase-8^{C360S} were re-expressed in the cells.

(**p**) Several potential Asp sites for caspase-8 cleavage, at D231, D232, D233, D240 and D270 in the GSDMC molecule are highlighted with red markers (top). Detection of GSDMC cleavage in different mutants after the treatment of cells with DM-αKG (bottom). Different point mutants as indicated were transfected into HeLa cells. $GSDMC^{WT}$ was used as a positive control.

(q) The cleavage of $GSDMC^{WT}$ and $GSDMC^{D240A}$ by recombinant caspase-8 protein *in vitro*. $GSDMC^{WT}$ and $GSDMC^{D240A}$ were immunoprecipitated from HEK293T cells that were transfected with Flag-HA-$GSDMC^{WT}$ or Flag-HA-$GSDMC^{D240A}$. The immunoprecipitated proteins were incubated with recombinant caspase-8 proteins.

(r) GSDMC was knocked out in HeLa cells based on CRISPR/Cas9 first, $GSDMC^{WT}$ or $GSDMC^{D240A}$ was then transfected into GSDMC KO pool cells, and pyroptosis upon DM-αKG stimulation was determined.

(s) The amino acid sequences flanking the cleavage site of GSDMC in human and mouse were shown.

(t) Overexpressed HA-mGSDMC1-4 in B16 cells were immunoprecipitated and detected

using anti-GSDMC antibody.

(**u**) Flag-tagged mouse GSDMC1-4 was transfected into B16 cells separately, and the cleavages of GSDMCs upon DM-αKG stimulation were determined. Z-VAD was used to pretreated with cells for 2 hours.

(**v**) Mouse GSDMC4 was specifically knocked down, the DM-αKG-induced cleavage of GSDMC, pyroptotic morphology and LDH release were detected.

(**w**) The cleavage of mouse GSDMC4 or GSDMC4^{D233A} was determined. B16 cells that were transfected with GSDMC4 or GSDMC4^{D233A} were treated with DM-αKG (left). GSDMC4 or GSDMC4^{D233A} proteins that was immunoprecipitated from GSDMC4 or GSDMC4^{D233A} overexpressed B16 cells was incubated with recombinant caspase-8 protein *in vitro* (right).

(x) The expression levels of GSDMC-WT-HBD*-HA and GSDMC-1-240-HBD*-HA in HeLa cells.

Tubulin was used to determine the amount of loading proteins. All data are presented as the mean±SEM of two or three independent experiments. *** $p<0.001$, ns: not significant. The data were analyzed using one-way ANOVA followed by Dunnett's multiple comparison test in (r) or two-way ANOVA followed by the Bonferroni test in (e, f, g, h, i, m, n, v).

Supplementary information, Figure S1

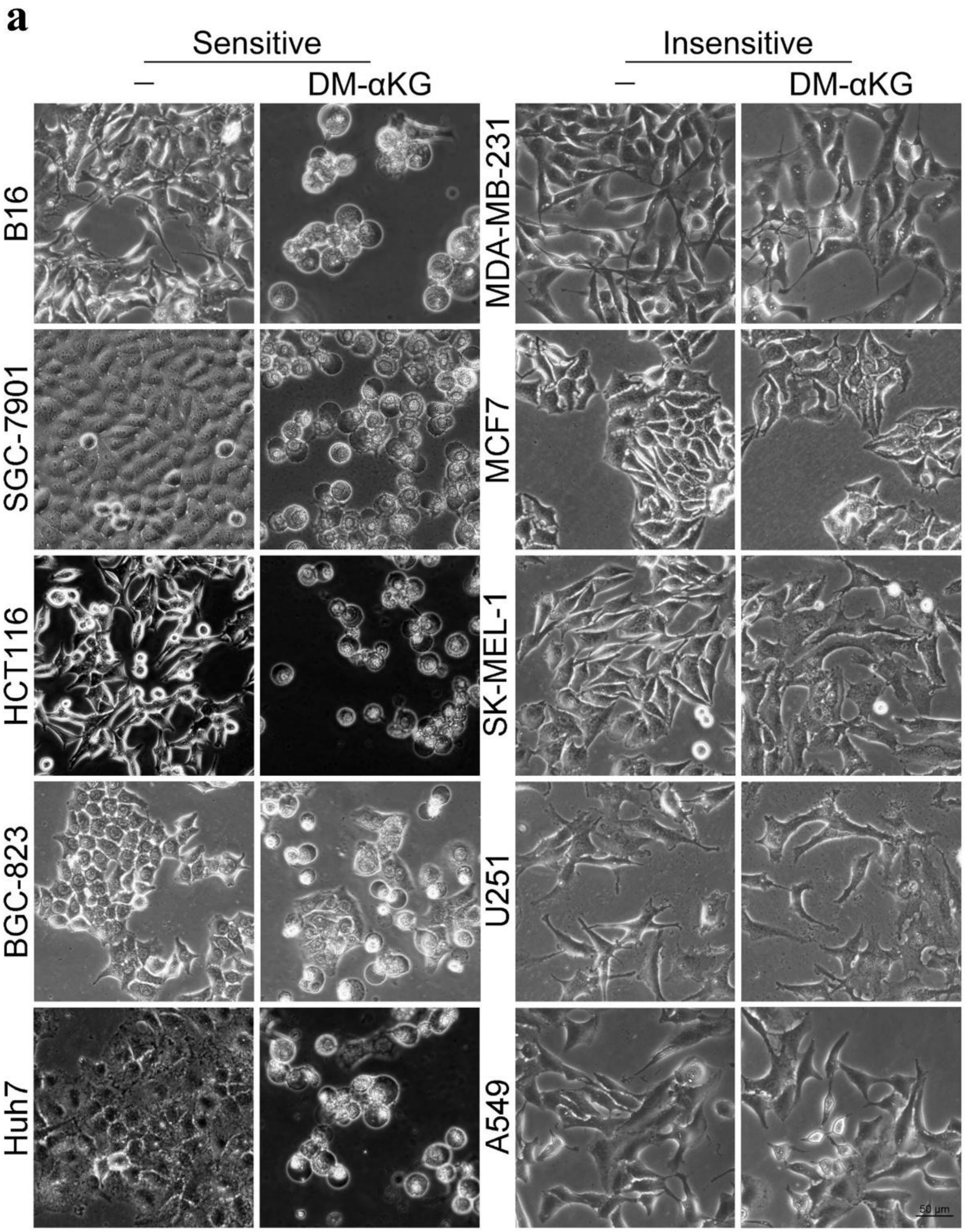

b

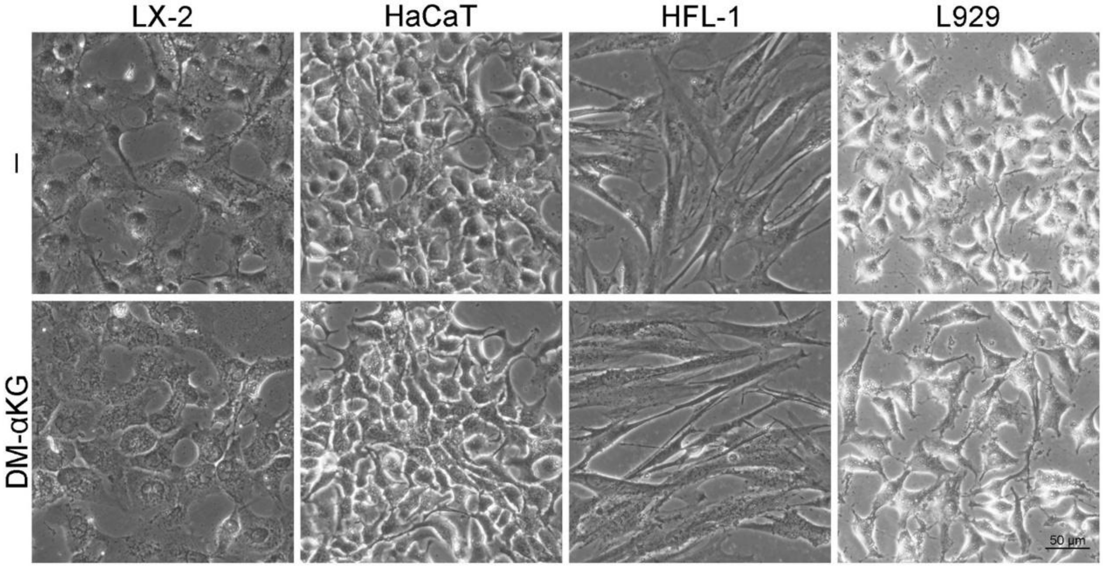
LX-2
HaCaT
HFL-1
L929
–
DM-αKG
50 μm

c

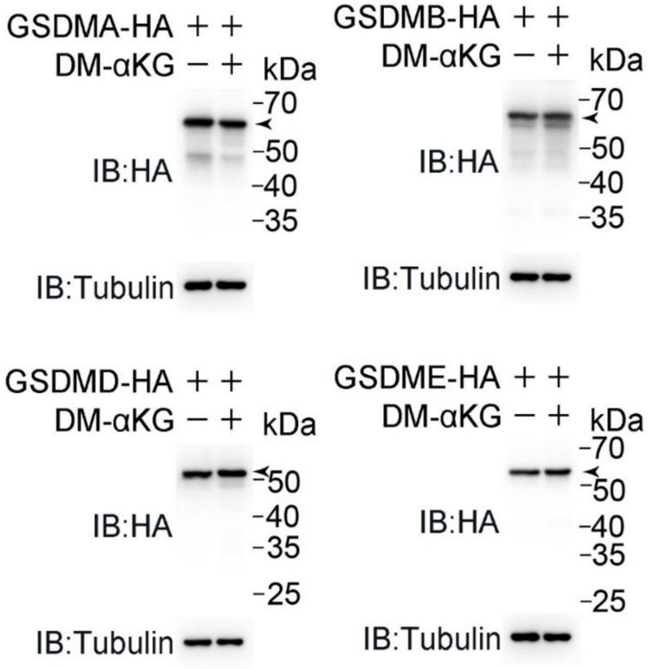
GSDMA-HA + +
DM-αKG – +
kDa
-70
-50
-40
-35
IB:HA
IB:Tubulin
GSDMB-HA + +
DM-αKG – +
kDa
-70
-50
-40
-35
IB:HA
IB:Tubulin
GSDMD-HA + +
DM-αKG – +
kDa
-50
-40
-35
-25
IB:HA
IB:Tubulin
GSDME-HA + +
DM-αKG – +
kDa
-70
-50
-40
-35
-25
IB:HA
IB:Tubulin

d

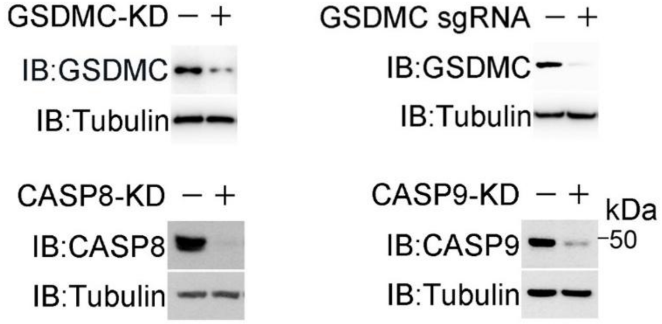
GSDMC-KD − +
IB:GSDMC
IB:Tubulin
GSDMC sgRNA − +
IB:GSDMC
IB:Tubulin
CASP8-KD − +
IB:CASP8
IB:Tubulin
CASP9-KD − +
kDa
IB:CASP9
-50
IB:Tubulin

e

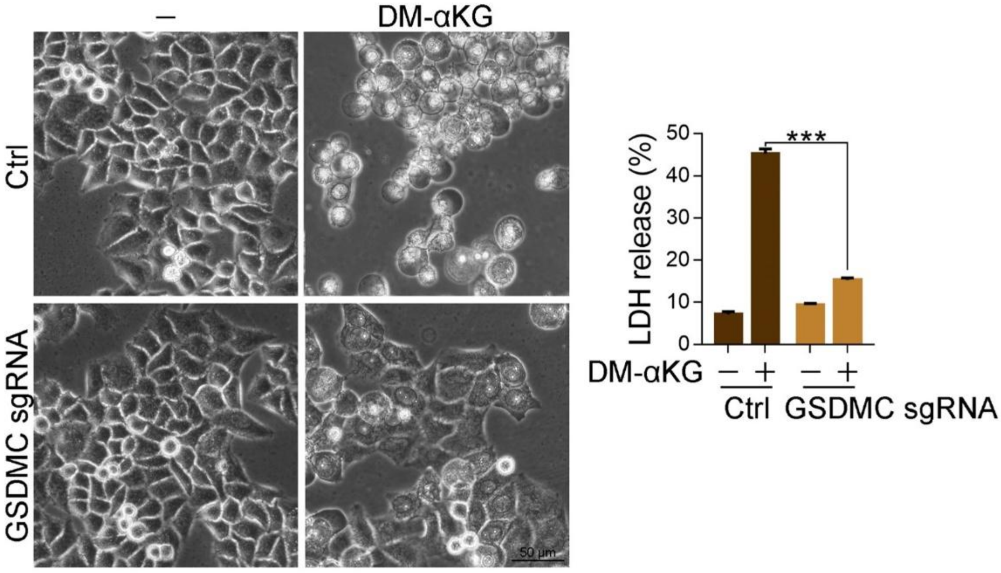
−
DM-αKG
Ctrl
GSDMC sgRNA
50 μm
LDH release (%)
50
40
30
20
10
0
***
DM-αKG − + − +
Ctrl
GSDMC sgRNA

f

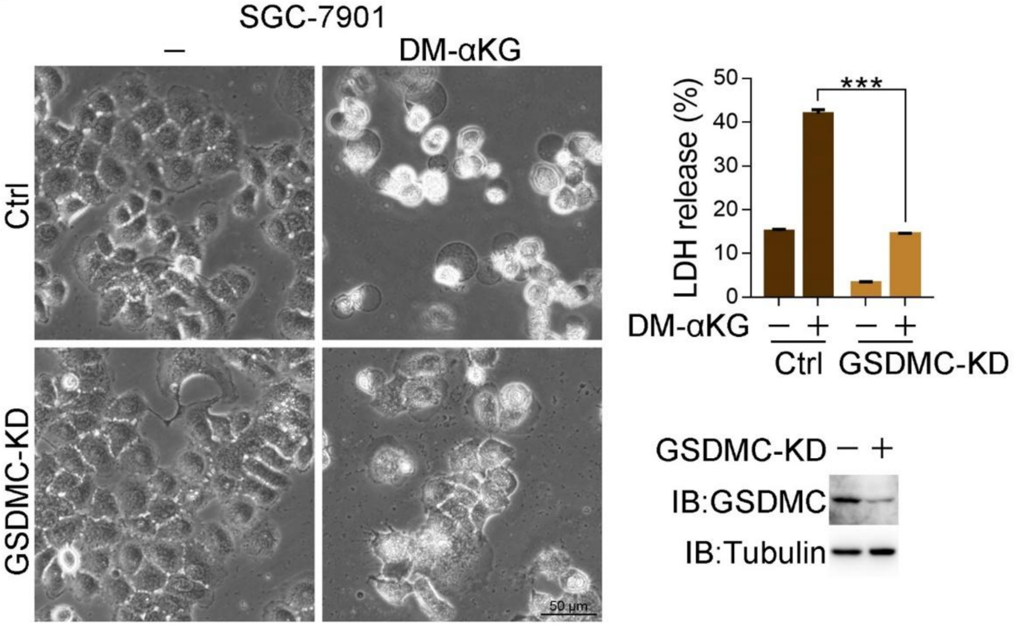
SGC-7901
—
DM-αKG
Ctrl
GSDMC-KD
50 μm
LDH release (%)
50
40
30
20
10
0
***
DM-αKG
— +
— +
Ctrl
GSDMC-KD
GSDMC-KD — +
IB:GSDMC
IB:Tubulin

g

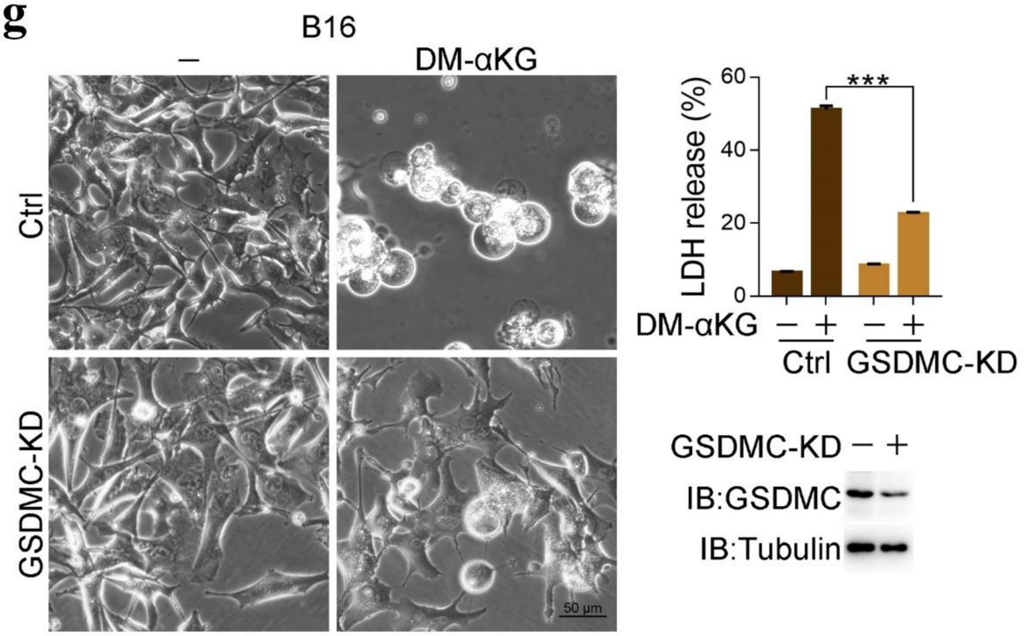
B16
—
DM-αKG
Ctrl
GSDMC-KD
50 μm
LDH release (%)
60
40
20
0
***
DM-αKG
— +
— +
Ctrl
GSDMC-KD
GSDMC-KD — +
IB:GSDMC
IB:Tubulin

h

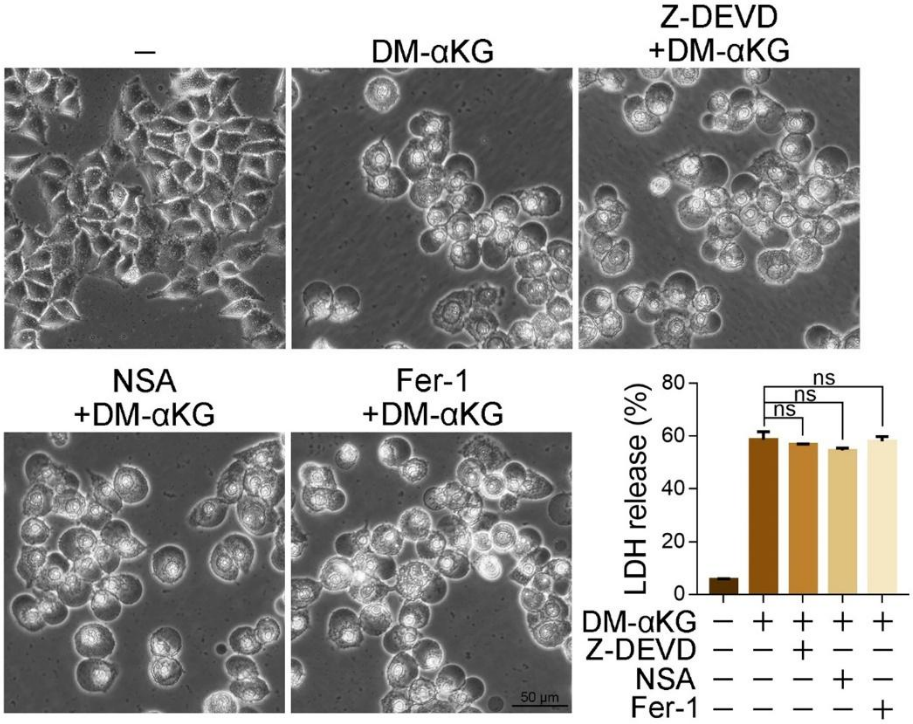
–
DM-αKG
Z-DEVD
+DM-αKG
NSA
+DM-αKG
Fer-1
+DM-αKG
50 μm
LDH release (%)
80
60
40
20
0
ns
ns
ns
DM-αKG – + + + +
Z-DEVD – – + – –
NSA – – – + –
Fer-1 – – – – +

i

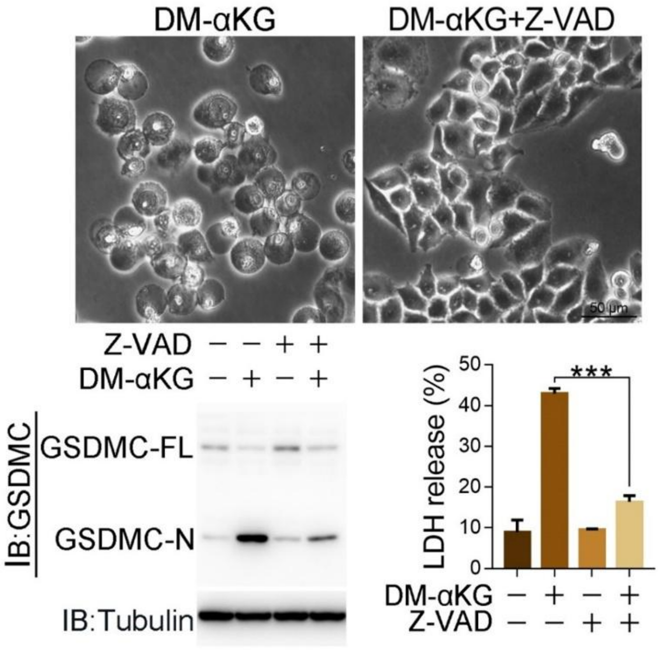
DM-αKG
DM-αKG+Z-VAD
50 μm
Z-VAD – – + +
DM-αKG – + – +
IB:GSDMC
GSDMC-FL
GSDMC-N
IB:Tubulin
LDH release (%)
50
40
30
20
10
0
***
DM-αKG – + – +
Z-VAD – – + +

j

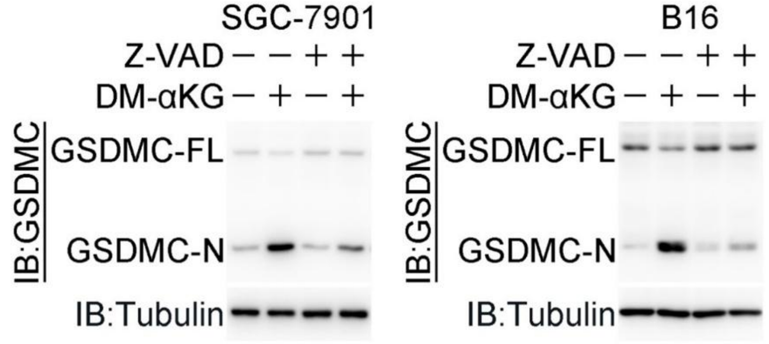
SGC-7901
Z-VAD − − + +
DM-αKG − + − +
IB:GSDMC
GSDMC-FL
GSDMC-N
IB:Tubulin
B16
Z-VAD − − + +
DM-αKG − + − +
IB:GSDMC
GSDMC-FL
GSDMC-N
IB:Tubulin

k

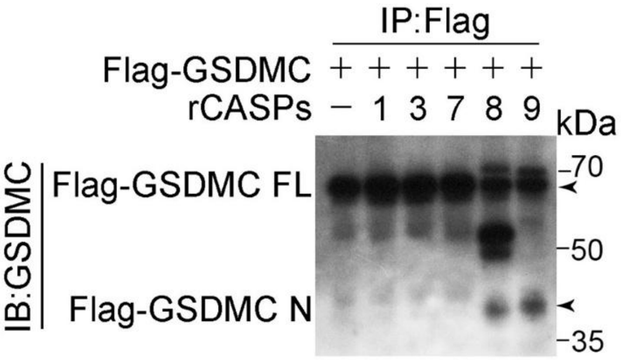
IP:Flag
Flag-GSDMC + + + + + +
rCASPs − 1 3 7 8 9
kDa
IB:GSDMC
Flag-GSDMC FL
Flag-GSDMC N
70
50
35

l

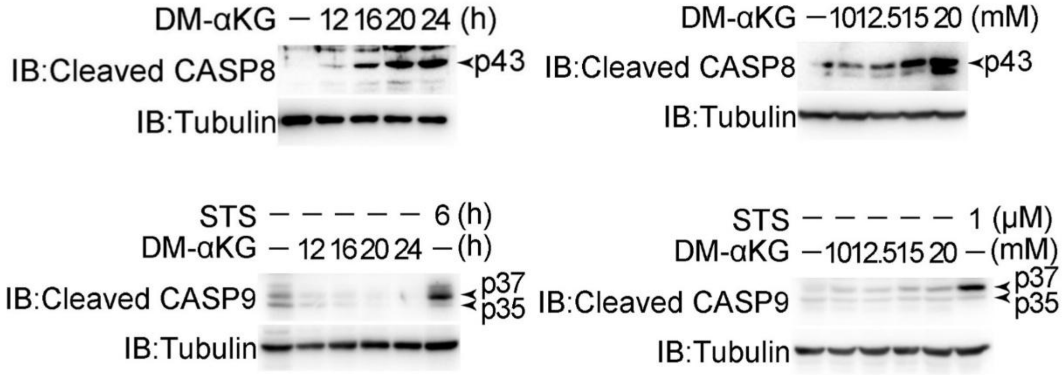
DM-αKG — 12 16 20 24 (h)
IB:Cleaved CASP8
p43
IB:Tubulin
DM-αKG — 10 12.5 15 20 (mM)
IB:Cleaved CASP8
p43
IB:Tubulin
STS — — — — — 6 (h)
DM-αKG — 12 16 20 24 — (h)
IB:Cleaved CASP9
p37
p35
IB:Tubulin
STS — — — — — 1 (μM)
DM-αKG — 10 12.5 15 20 — (mM)
IB:Cleaved CASP9
p37
p35
IB:Tubulin

m

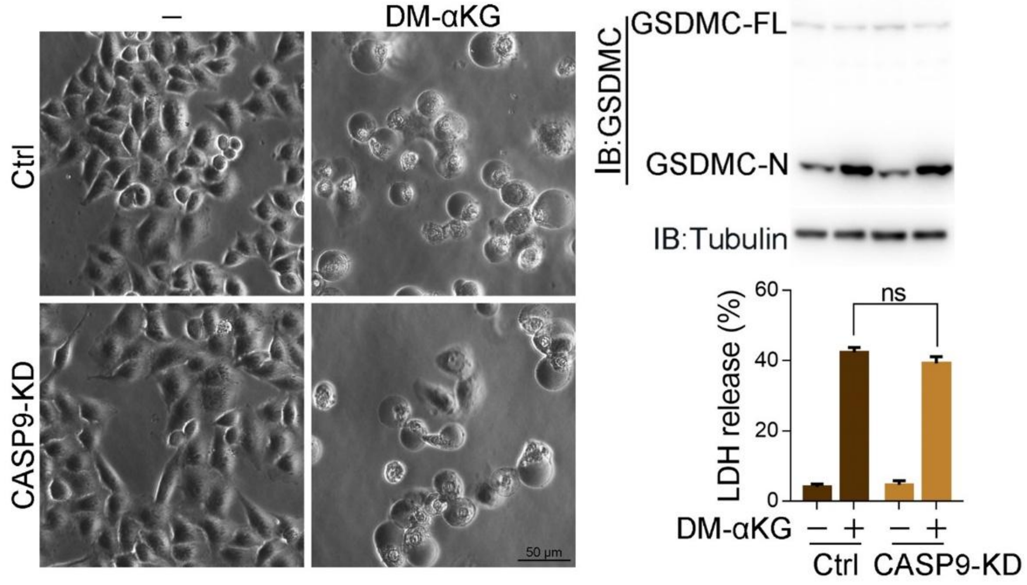
—
DM-αKG
Ctrl
CASP9-KD
50 μm
IB:GSDMC
GSDMC-FL
GSDMC-N
IB:Tubulin
LDH release (%)
60
40
20
0
ns
DM-αKG − + − +
Ctrl
CASP9-KD

n

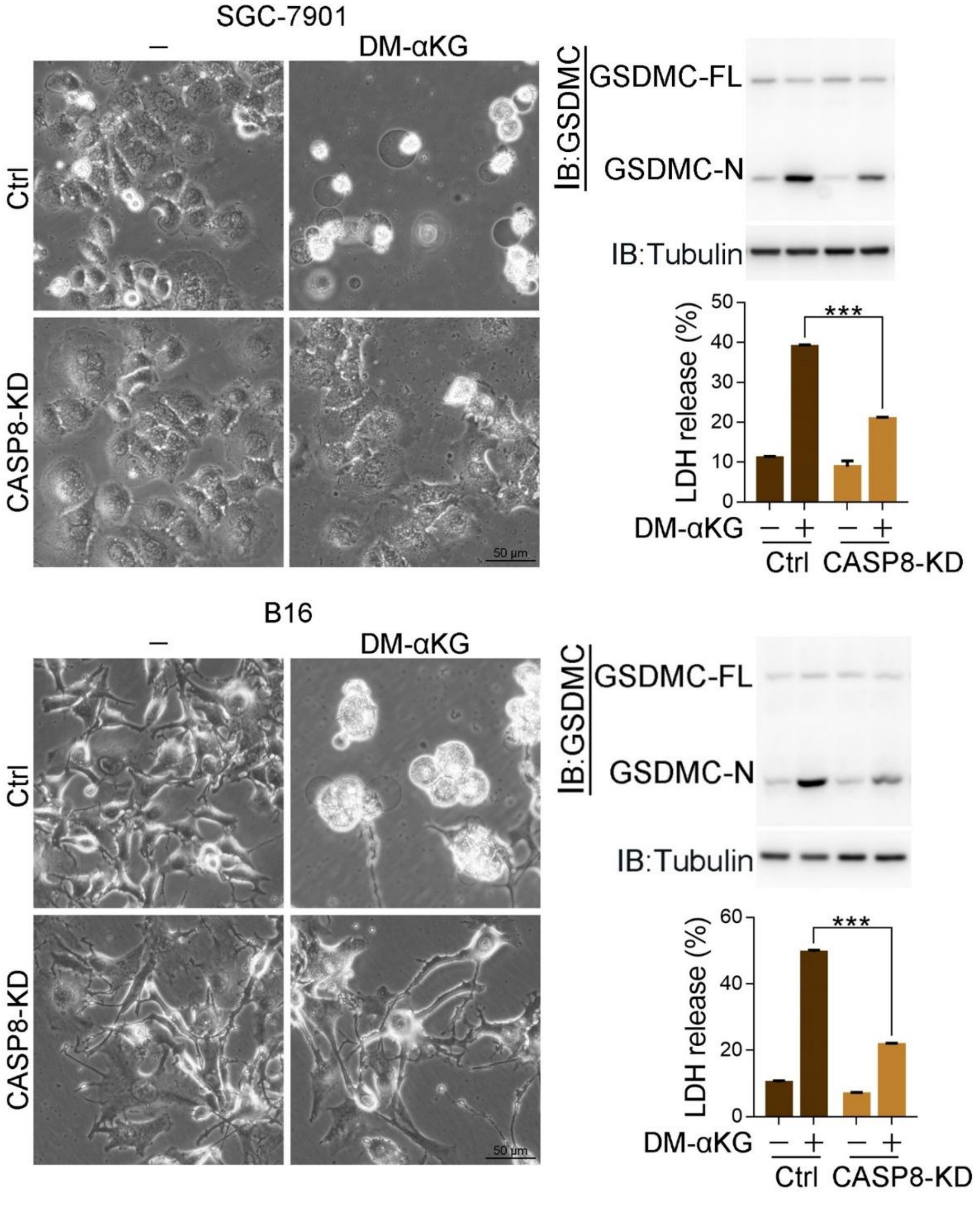
SGC-7901
–
DM-αKG
Ctrl
CASP8-KD
50 μm
IB:GSDMC
GSDMC-FL
GSDMC-N
IB:Tubulin
LDH release (%)
0
10
20
30
40
50
***
DM-αKG
– + – +
Ctrl
CASP8-KD
B16
–
DM-αKG
Ctrl
CASP8-KD
50 μm
IB:GSDMC
GSDMC-FL
GSDMC-N
IB:Tubulin
LDH release (%)
0
20
40
60
***
DM-αKG
– + – +
Ctrl
CASP8-KD

o

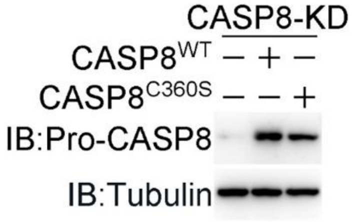

CASP8-KD
CASP8^WT − + −
CASP8^C360S − − +
IB:Pro-CASP8
IB:Tubulin

p

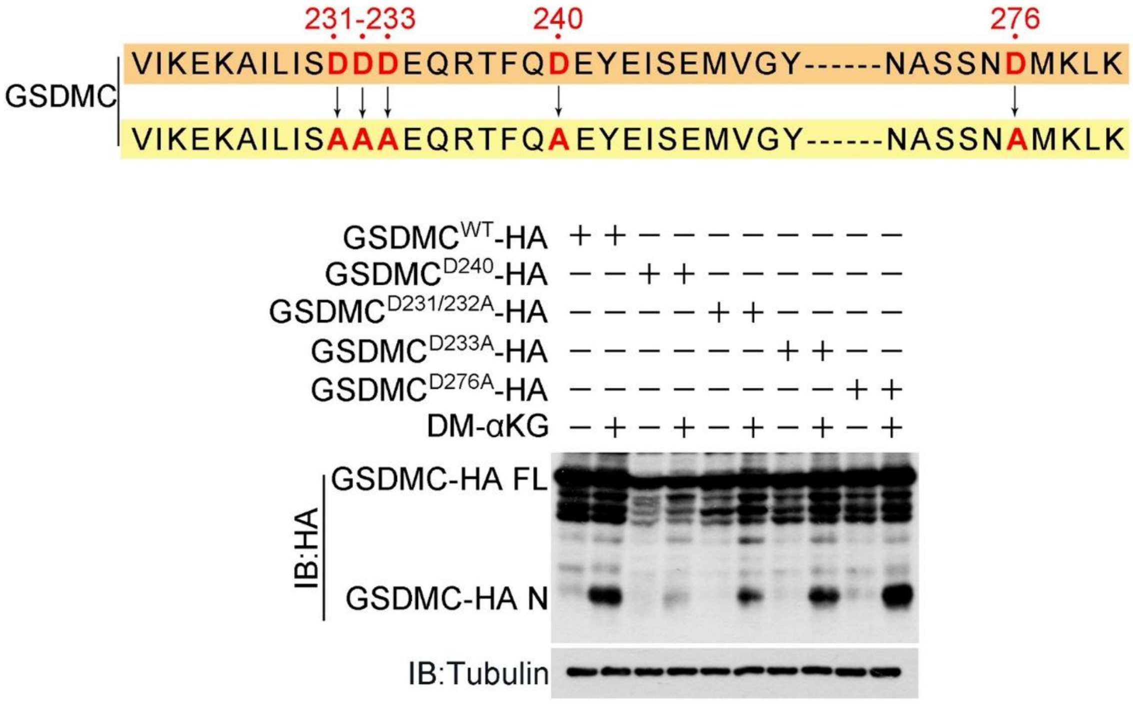

231-233
240
276
GSDMC
VIKEKAILISDDDEQRTFQDEYEISEMVGY------NASSNDMKLK
VIKEKAILISAAAEQRTFQAEYEISEMVGY------NASSNAMKLK
GSDMC^WT-HA + + − − − − − − − −
GSDMC^D240-HA − − + + − − − − − −
GSDMC^D231/232A-HA − − − − + + − − − −
GSDMC^D233A-HA − − − − − − + + − −
GSDMC^D276A-HA − − − − − − − − + +
DM-αKG − + − + − + − + − +
IB:HA
GSDMC-HA FL
GSDMC-HA N
IB:Tubulin

q

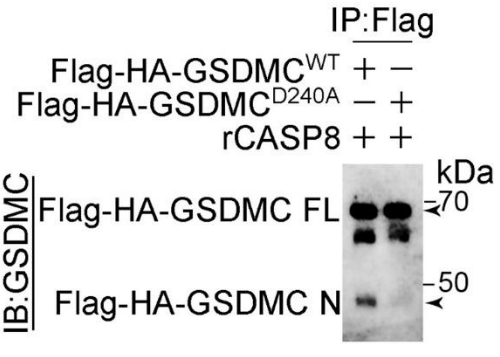
IP:Flag
Flag-HA-GSDMC^WT + −
Flag-HA-GSDMC^D240A − +
rCASP8 + +
kDa
IB:GSDMC
Flag-HA-GSDMC FL
70
50
Flag-HA-GSDMC N

r

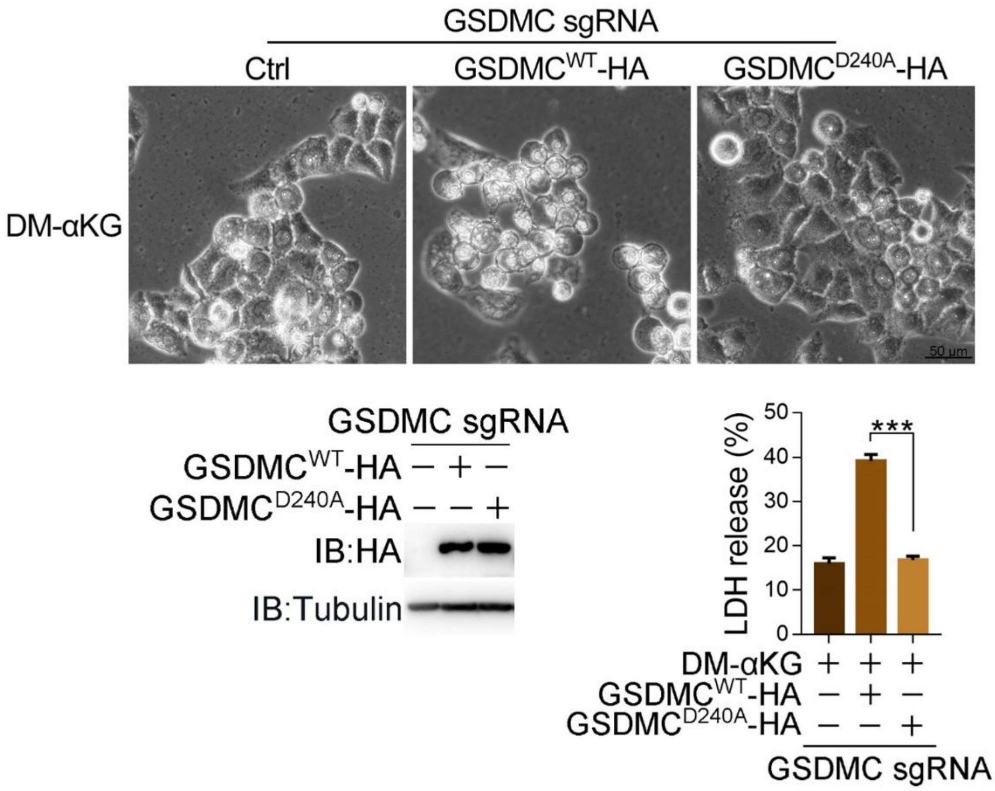
GSDMC sgRNA
Ctrl
GSDMC^WT-HA
GSDMC^D240A-HA
DM-αKG
50 μm
GSDMC sgRNA
GSDMC^WT-HA − + −
GSDMC^D240A-HA − − +
IB:HA
IB:Tubulin
LDH release (%)
0
10
20
30
40
50
***
DM-αKG + + +
GSDMC^WT-HA − + −
GSDMC^D240A-HA − − +
GSDMC sgRNA

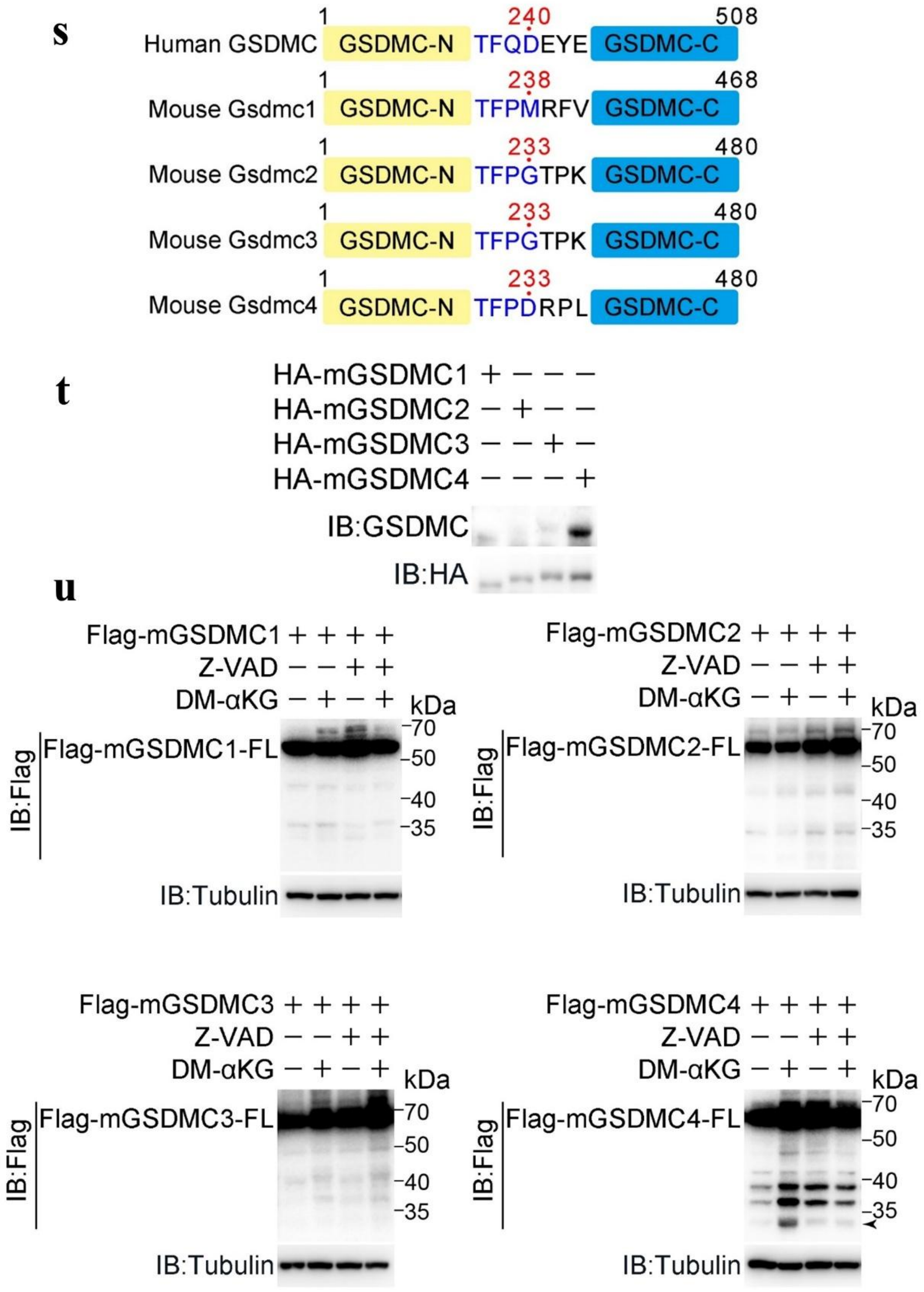
s
Human GSDMC
Mouse Gsdmc1
Mouse Gsdmc2
Mouse Gsdmc3
Mouse Gsdmc4
GSDMC-N
GSDMC-C
1
240
508
TFQDEYE
238
468
TFPMRFV
233
480
TFPGTPK
TFPDRPL
t
HA-mGSDMC1 + − − −
HA-mGSDMC2 − + − −
HA-mGSDMC3 − − + −
HA-mGSDMC4 − − − +
IB:GSDMC
IB:HA
u
Flag-mGSDMC1 + + + +
Flag-mGSDMC2 + + + +
Flag-mGSDMC3 + + + +
Flag-mGSDMC4 + + + +
Z-VAD − − + +
DM-αKG − + − +
kDa
70
50
40
35
IB:Flag
Flag-mGSDMC1-FL
Flag-mGSDMC2-FL
Flag-mGSDMC3-FL
Flag-mGSDMC4-FL
IB:Tubulin

V

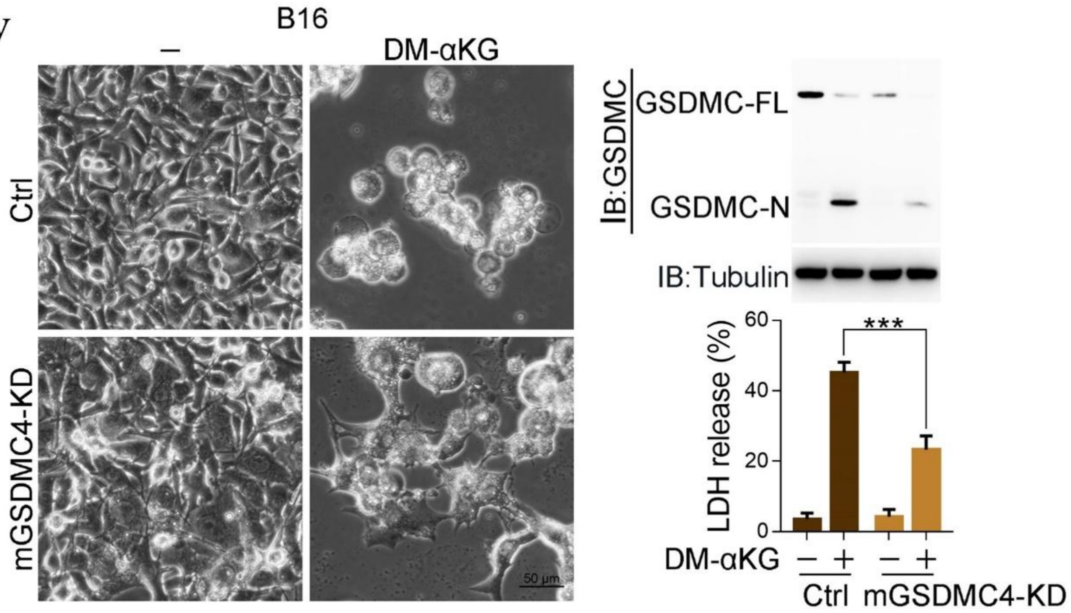
B16
–
DM-αKG
Ctrl
mGSDMC4-KD
50 μm
IB:GSDMC
GSDMC-FL
GSDMC-N
IB:Tubulin
LDH release (%)
60
40
20
0
***
DM-αKG – + – +
Ctrl
mGSDMC4-KD

W

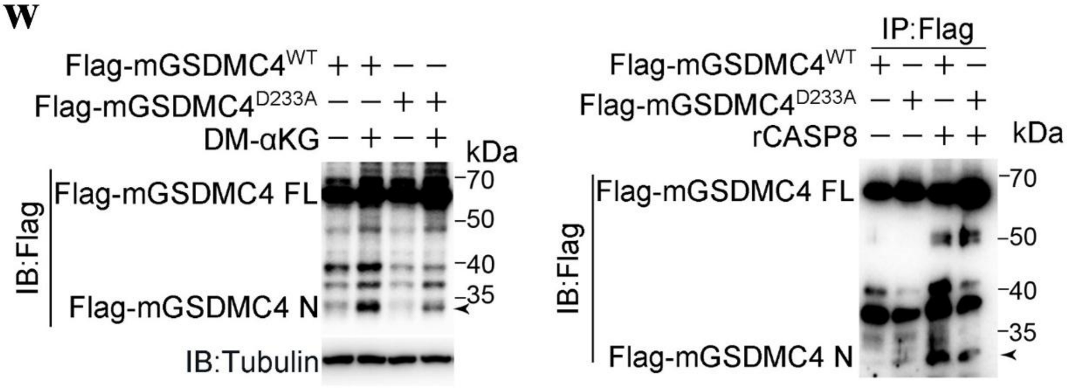
Flag-mGSDMC4^WT + + – –
Flag-mGSDMC4^D233A – – + +
DM-αKG – + – +
kDa
IB:Flag
Flag-mGSDMC4 FL
Flag-mGSDMC4 N
–70
–50
–40
–35
IB:Tubulin
IP:Flag
Flag-mGSDMC4^WT + – + –
Flag-mGSDMC4^D233A – + – +
rCASP8 – – + +
kDa
IB:Flag
Flag-mGSDMC4 FL
Flag-mGSDMC4 N
–70
–50
–40
–35

X

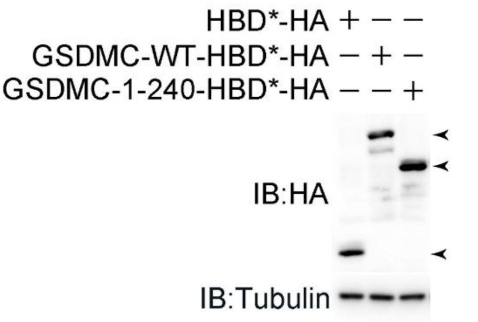
HBD*-HA + – –
GSDMC-WT-HBD*-HA – + –
GSDMC-1-240-HBD*-HA – – +
IB:HA
IB:Tubulin
