# Supplementary material for: The metabolite α-KG induces GSDMC-dependent pyroptosis through death receptor 6-activated caspase-8
Source: Cell Res. 2021 May 19;31(9):980–97. doi: 10.1038/s41422-021-00506-9 (PMC8410789; doi:10.1038/s41422-021-00506-9)
Supplement: Supplementary file 2 — Fig S2 [file 41422_2021_506_MOESM2_ESM.pdf]

Supplementary information, Fig. S2. In this figure, different cancer cells were treated with DM-αKG (15 mM) for 6 hours to determine the ROS levels or 24 hours to assess DR6 oxidation, pyroptotic features (including morphology, GSDMC cleavage, LDH release, and $Annv^{+}/PI^{+}$ cells), unless specially indicated otherwise.

(**a, b**) Effect of Trolox on DM-αKG-induced pyroptosis, as assessed by cell morphology, GSDMC cleavage, LDH release (a) and $Annv^{+}/PI^{+}$ cells (b) in HeLa cells. Trolox was used to pretreat cells for 2 hours.

(**c**) Effect of Trolox on DM-αKG-induced GSDMC cleavage in SGC-7901 (left) and B16 (right) cells.

(d) The siRNA-based knockdown (KO) or CRISPR/cas9-based knockout pool (sgRNA) of DR6, TNFR1, FAS, DR3, DR4 or DR5 in HeLa cells was determined by western blot or RT-qPCR analysis.

(**e**) Knocking down TNFR1, FAS, DR3, DR4 or DR5 had no effect on DM-αKG-induced pyroptosis, as indicated by cell morphology. Different genes indicated above had first been knocked down in HeLa cells.

(**f**) Knocking down DR6 impaired DM-αKG-induced pyroptosis, as assessed by cell morphology, GSDMC cleavage and LDH release in SGC-7901 (top) and B16 (bottom) cells.

(g) Knocking out DR6 impaired DM-αKG-induced pyroptosis in HeLa cells.

(**h**) DM-αKG induced the oxidation of DR6 in SGC-7901 cells.

(**i**) β-mercatoethanol abolished DR6 oxidation. Control or DM-αKG-treated HeLa cell lysates were boiled in loading buffer with or without β-mercatoethanol (0.713M) incubation.

(j) In DR6 knocking out HeLa cells DM-αKG did not induce the oxidation of DR6.

(k) Some ROS activators (including H_2O_2 (2 μM), Antimycin A (5 μM), Oligomycin (1 μM), $NaAsO_2$ (2.5 μM), and Rotenone (50 nM)) showed mild effects on DR6 oxidation. HeLa cells were treated with ROS activators for 24 hours.

(l) The amino acid sequences flanking Cys in C terminus of DR6 were shown. Mutation sequences from 1CS to 5CS in the DR6 molecule are highlighted with red markers as compared to the WT DR6.

(m) Comparison of DR6 oxidative levels in the C-terminal and N-terminal of DR6 in HeLa cells.

(**n-o**) Comparison of DM-αKG-induced oxidation levels among different DR6 single point (n) or multipoint (o) mutants. Different mutants of DR6 were transfected into HeLa cells as indicated.

(p-q) DR6 was knocked out first in HeLa cells based on CRISPR/Cas9. $DR6^{WT}$ or $DR6^{5CS}$

was then transfected into DR6 KO pool cells. Caspase-8 cleavage (p) and pyroptosis (q) upon DM-αKG stimulation was determined.

Tubulin was used to determine the amount of loading proteins. All data are presented as the mean±SEM of two or three independent experiments. *** $p<0.001$. The data were analyzed using one-way ANOVA followed by Dunnett's multiple comparison test in (q) or two-way ANOVA followed by the Bonferroni test in (a, b, f, g).

Supplementary information, Figure S2

a

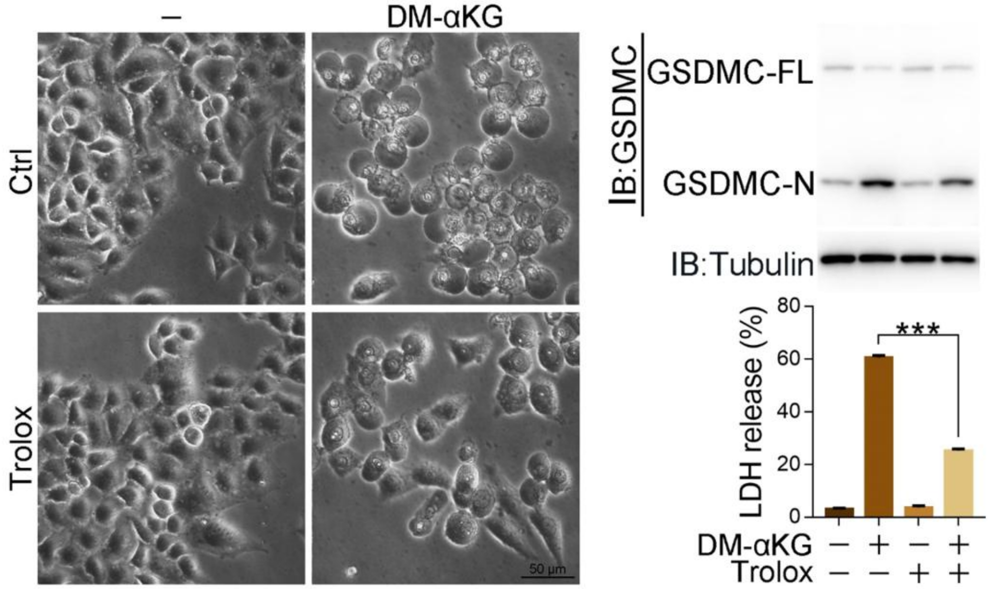

b

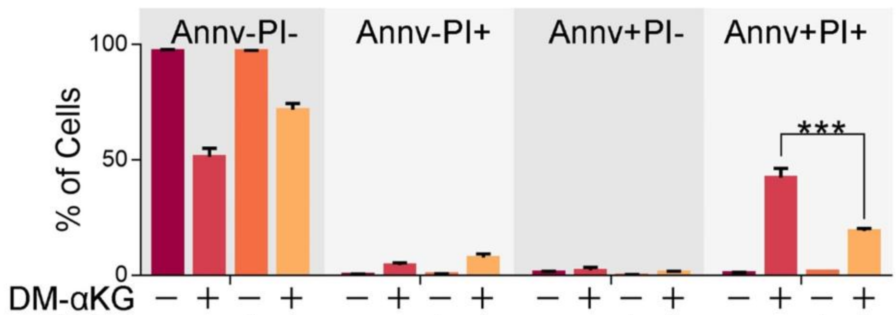

c

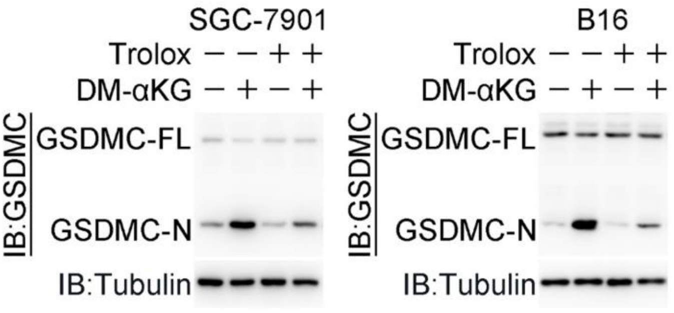
SGC-7901
Trolox − − + +
DM-αKG − + − +
IB:GSDMC
GSDMC-FL
GSDMC-N
IB:Tubulin
B16
Trolox − − + +
DM-αKG − + − +
IB:GSDMC
GSDMC-FL
GSDMC-N
IB:Tubulin

d

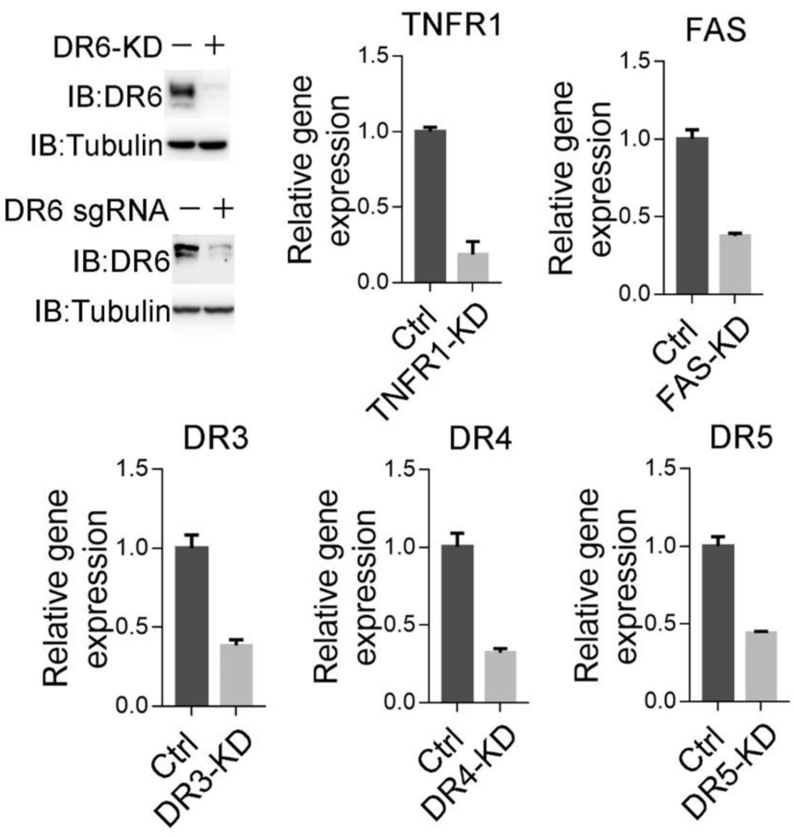
DR6-KD − +
IB:DR6
IB:Tubulin
DR6 sgRNA − +
IB:DR6
IB:Tubulin
TNFR1
FAS
DR3
DR4
DR5
Relative gene expression
0.0
0.5
1.0
1.5
Ctrl
TNFR1-KD
FAS-KD
DR3-KD
DR4-KD
DR5-KD

e

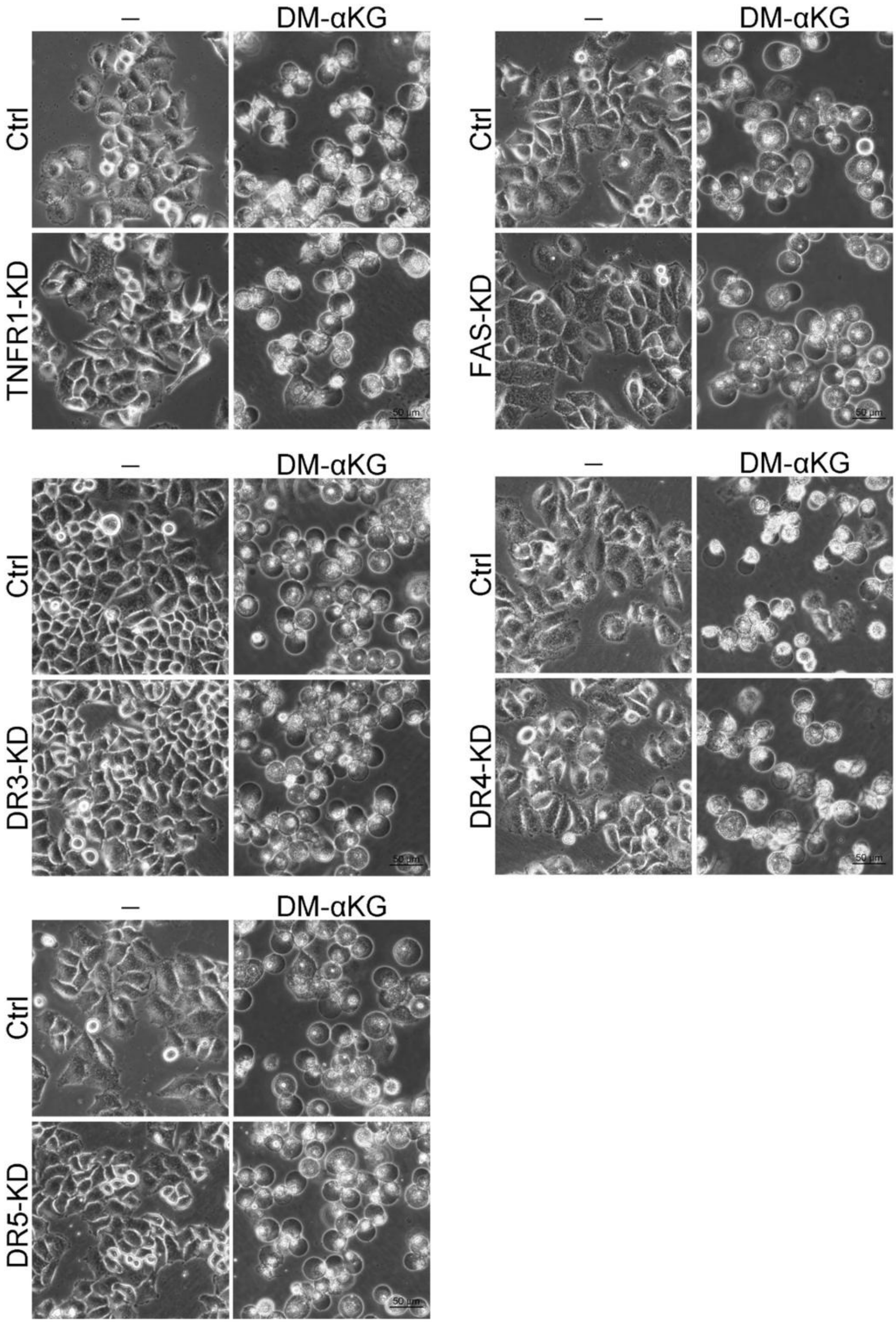
–
DM-αKG
Ctrl
TNFR1-KD
50 μm
–
DM-αKG
Ctrl
FAS-KD
50 μm
–
DM-αKG
Ctrl
DR3-KD
50 μm
–
DM-αKG
Ctrl
DR4-KD
50 μm
–
DM-αKG
Ctrl
DR5-KD
50 μm

f

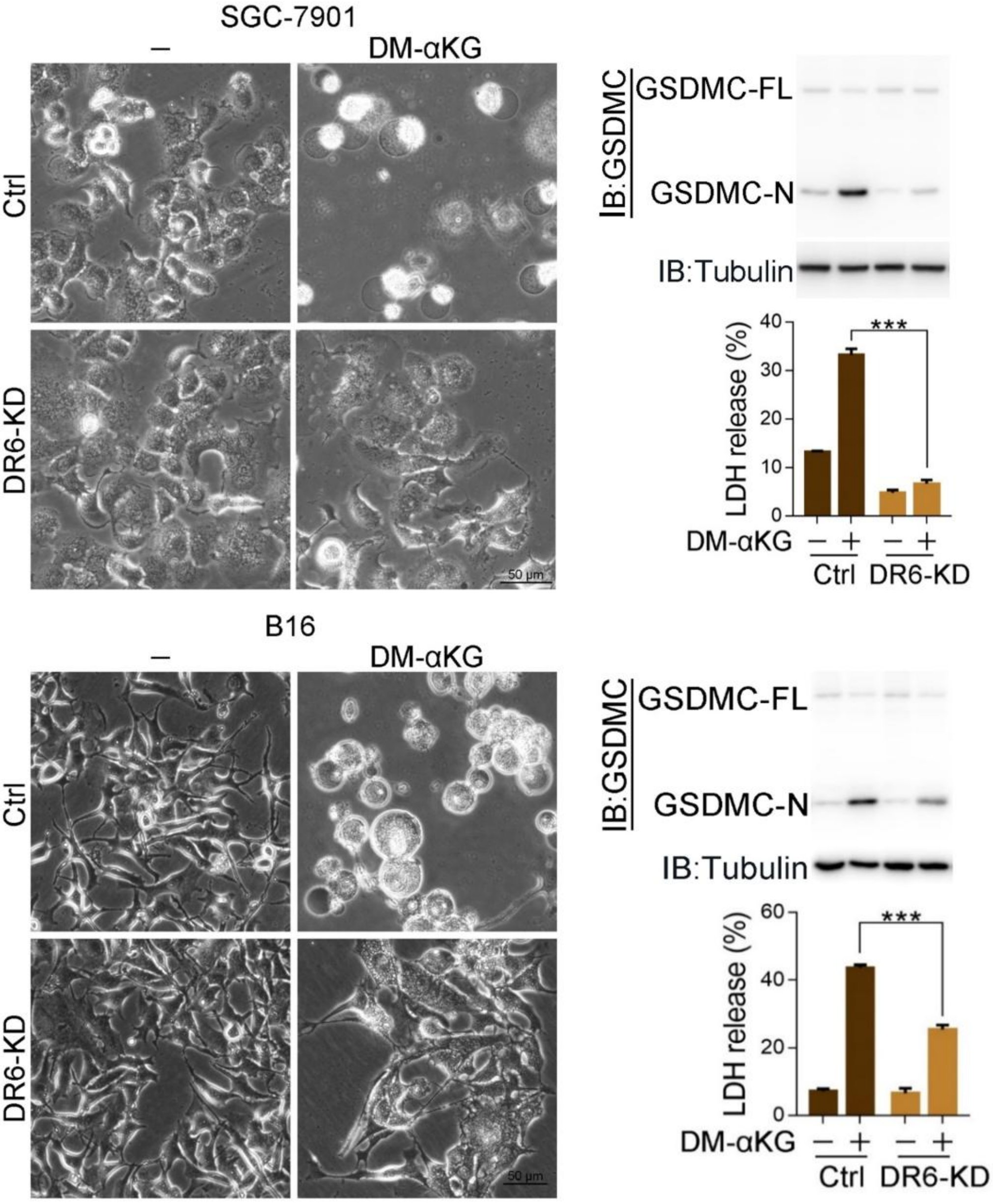

SGC-7901
–
DM-αKG
Ctrl
DR6-KD
50 μm
IB:GSDMC
GSDMC-FL
GSDMC-N
IB:Tubulin
LDH release (%)
***
DM-αKG
Ctrl
DR6-KD
B16
–
DM-αKG
Ctrl
DR6-KD
50 μm
IB:GSDMC
GSDMC-FL
GSDMC-N
IB:Tubulin
LDH release (%)
***
DM-αKG
Ctrl
DR6-KD

g

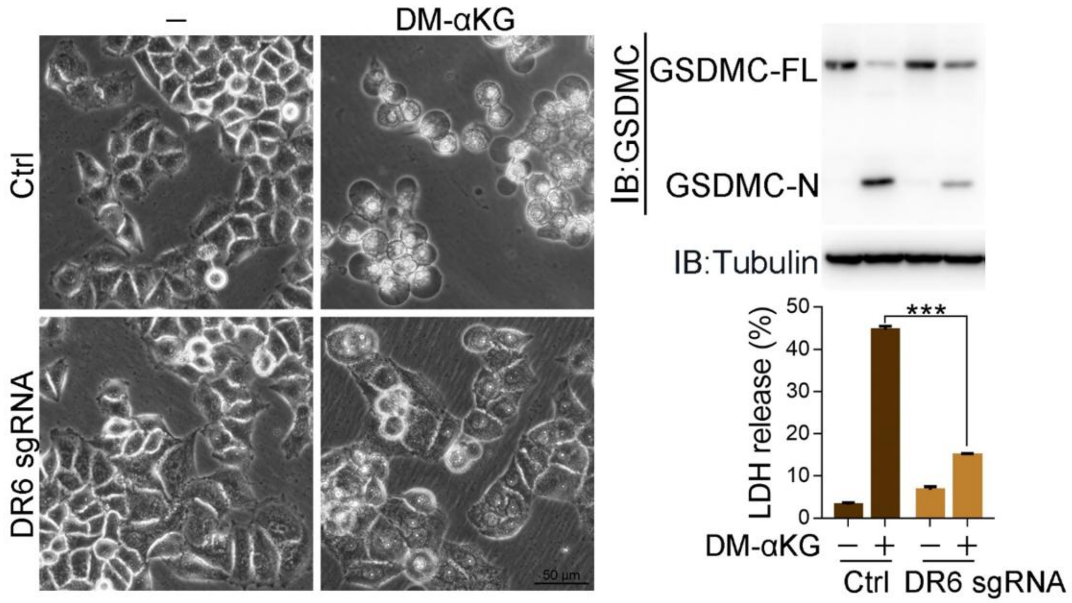

–
DM-αKG
Ctrl
DR6 sgRNA
50 μm
IB:GSDMC
GSDMC-FL
GSDMC-N
IB:Tubulin
LDH release (%)
50
40
30
20
10
0
***
DM-αKG
– + – +
Ctrl
DR6 sgRNA

h

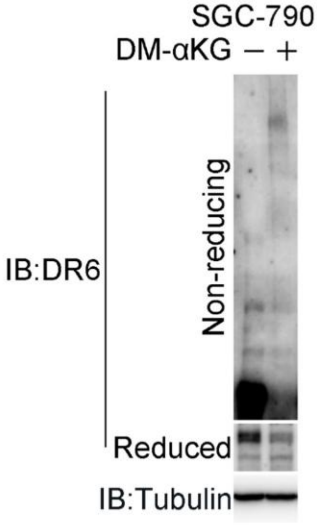

SGC-7901
DM-αKG – +
IB:DR6
Non-reducing
Reduced
IB:Tubulin

i

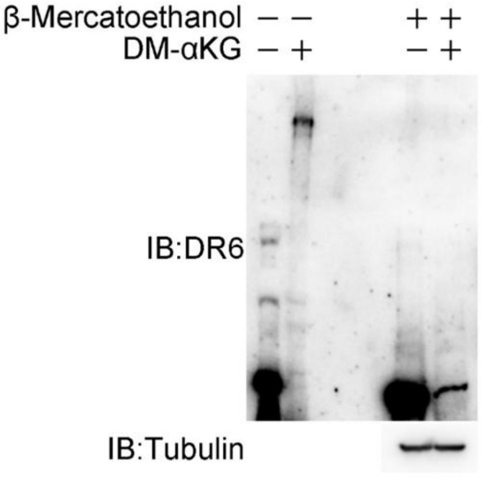

β-Mercatoethanol – – + +
DM-αKG – + – +
IB:DR6
IB:Tubulin

j

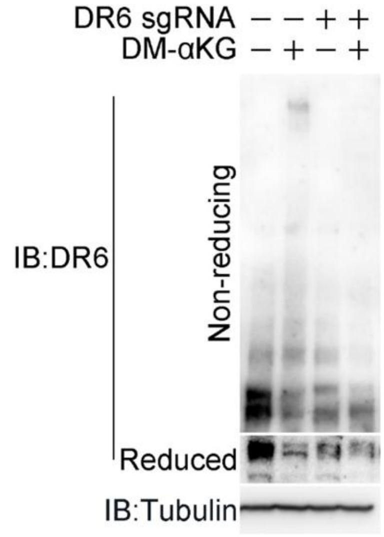
DR6 sgRNA − − + +
DM-αKG − + − +
IB:DR6
Non-reducing
Reduced
IB:Tubulin

k

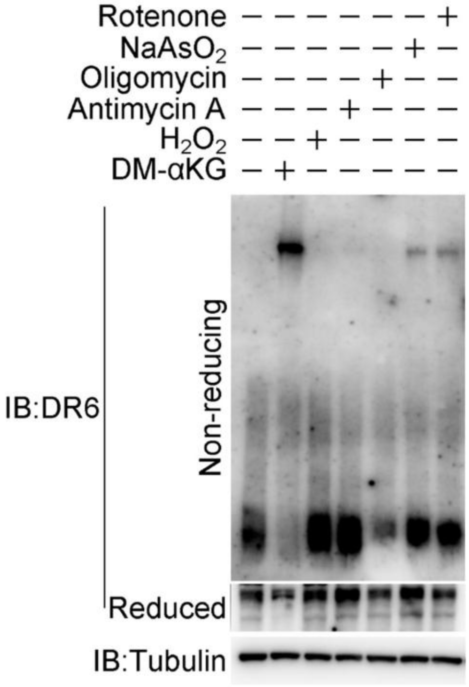
Rotenone − − − − − − +
NaAsO2 − − − − − + −
Oligomycin − − − − + − −
Antimycin A − − − + − − −
H2O2 − − + − − − −
DM-αKG − + − − − − −
IB:DR6
Non-reducing
Reduced
IB:Tubulin

l

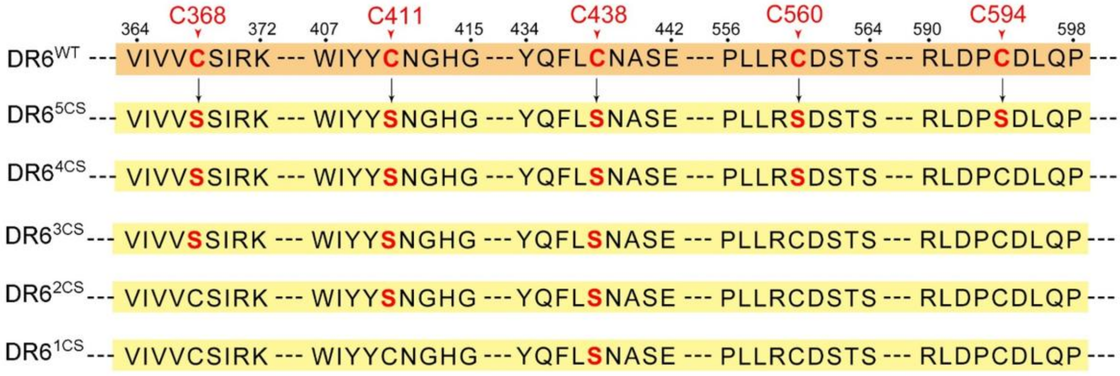
C368 C411 C438 C560 C594
364 372 407 415 434 442 556 564 590 598
DR6WT --- VIVVCSIRK --- WIYYCNGHG --- YQFLCNASE --- PLLRCDSTS --- RLDPCDLQP ---
DR6 5CS --- VIVVSSIRK --- WIYYSNGHG --- YQFLSNASE --- PLLRSDSTS --- RLDPSDLQP ---
DR6 4CS --- VIVVSSIRK --- WIYYSNGHG --- YQFLSNASE --- PLLRSDSTS --- RLDPCDLQP ---
DR6 3CS --- VIVVSSIRK --- WIYYSNGHG --- YQFLSNASE --- PLLRCDSTS --- RLDPCDLQP ---
DR6 2CS --- VIVVCSIRK --- WIYYSNGHG --- YQFLSNASE --- PLLRCDSTS --- RLDPCDLQP ---
DR6 1CS --- VIVVCSIRK --- WIYYCNGHG --- YQFLSNASE --- PLLRCDSTS --- RLDPCDLQP ---

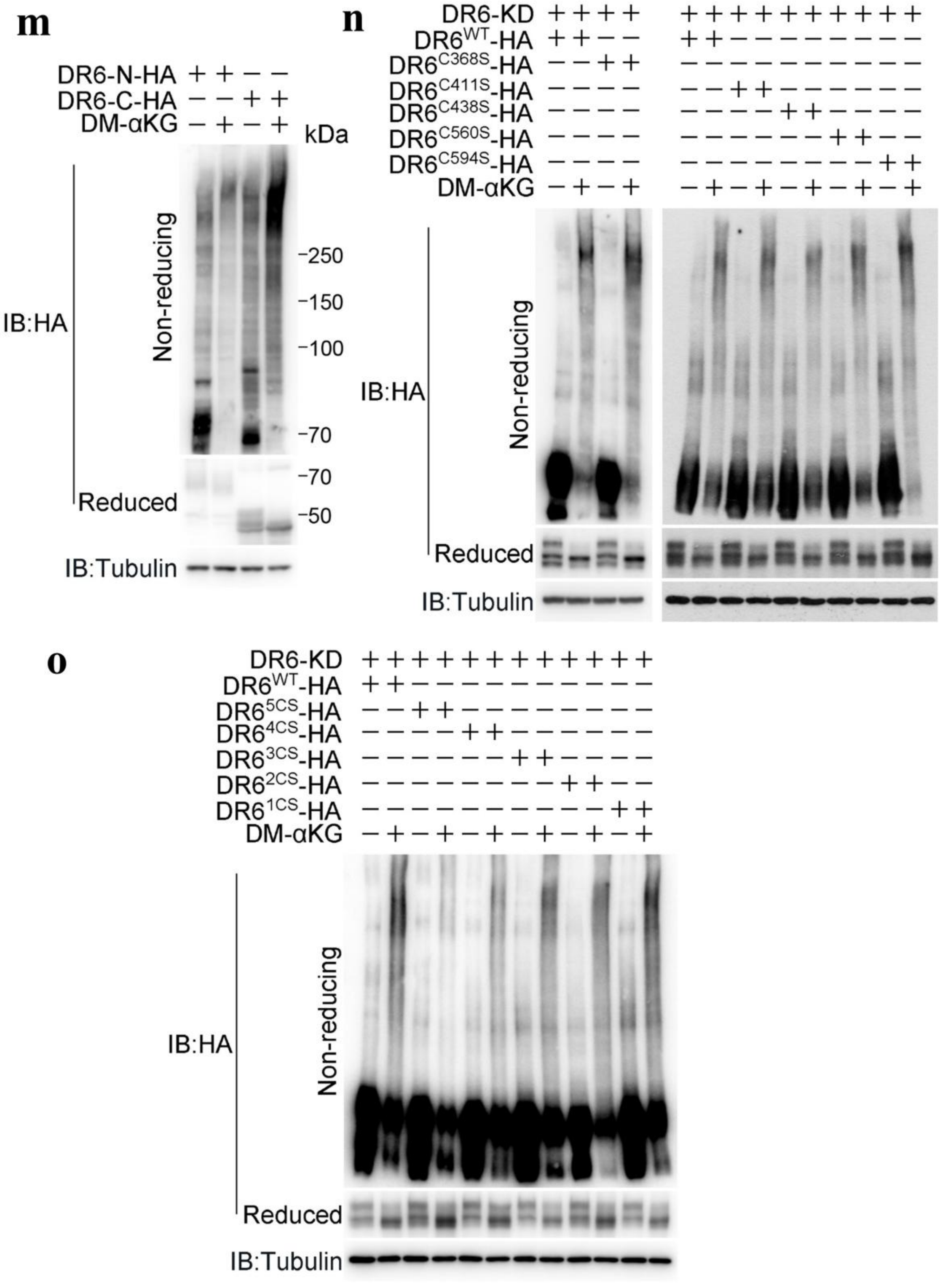

m
DR6-N-HA + + − −
DR6-C-HA − − + +
DM-αKG − + − +
kDa
IB:HA
Non-reducing
250
150
100
70
Reduced
70
50
IB:Tubulin
n
DR6-KD + + + + + + + + + + + + + +
DR6WT-HA + + − − + + − − − − − − − −
DR6C368S-HA − − + + − − − − − − − − − −
DR6C411S-HA − − − − − − + + − − − − − −
DR6C438S-HA − − − − − − − − + + − − − −
DR6C560S-HA − − − − − − − − − − + + − −
DR6C594S-HA − − − − − − − − − − − − + +
DM-αKG − + − + − + − + − + − + − +
IB:HA
Non-reducing
Reduced
IB:Tubulin
o
DR6-KD + + + + + + + + + + + +
DR6WT-HA + + − − − − − − − − − −
DR65CS-HA − − + + − − − − − − − −
DR64CS-HA − − − − + + − − − − − −
DR63CS-HA − − − − − − + + − − − −
DR62CS-HA − − − − − − − − + + − −
DR61CS-HA − − − − − − − − − − + +
DM-αKG − + − + − + − + − + − +
IB:HA
Non-reducing
Reduced
IB:Tubulin

p

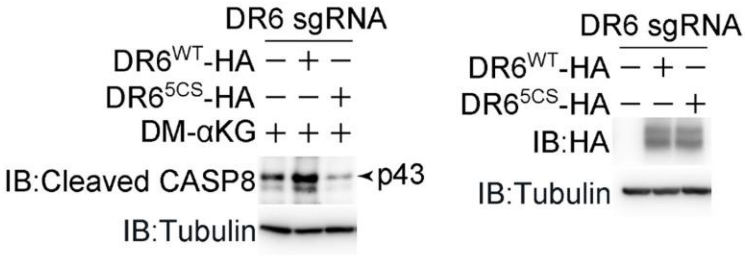
DR6 sgRNA
DR6WT-HA − + −
DR65CS-HA − − +
DM-αKG + + +
IB:Cleaved CASP8
p43
IB:Tubulin
DR6 sgRNA
DR6WT-HA − + −
DR65CS-HA − − +
IB:HA
IB:Tubulin

q

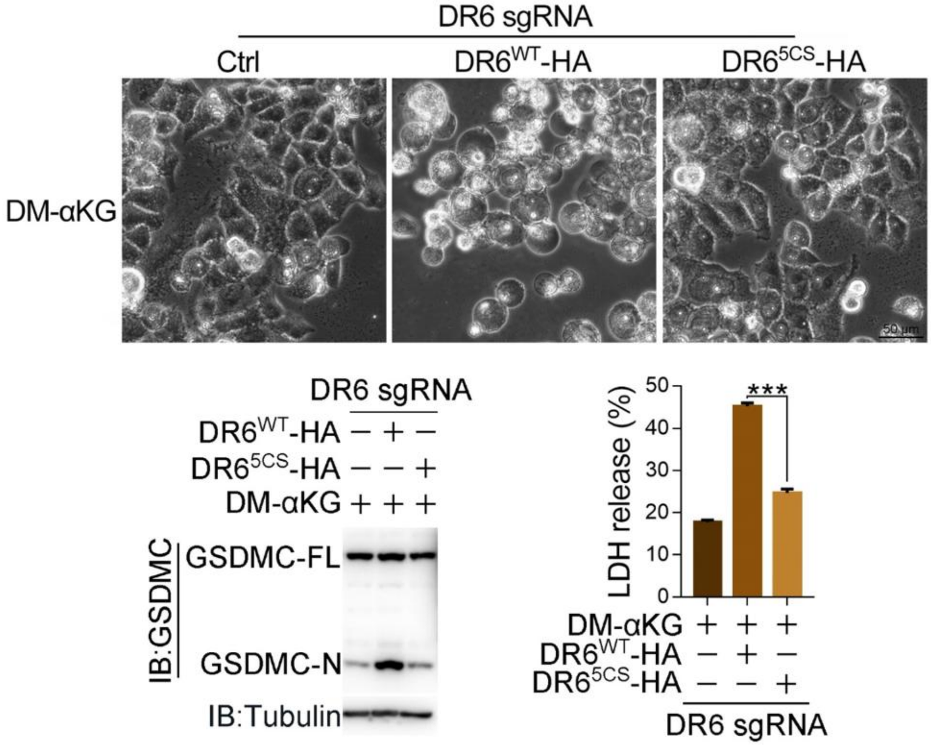
DR6 sgRNA
Ctrl
DR6WT-HA
DR65CS-HA
DM-αKG
50 μm
DR6 sgRNA
DR6WT-HA − + −
DR65CS-HA − − +
DM-αKG + + +
IB:GSDMC
GSDMC-FL
GSDMC-N
IB:Tubulin
LDH release (%)
0
10
20
30
40
50
***
DM-αKG + + +
DR6WT-HA − + −
DR65CS-HA − − +
DR6 sgRNA
