# Supplementary material for: The metabolite α-KG induces GSDMC-dependent pyroptosis through death receptor 6-activated caspase-8
Source: Cell Res. 2021 May 19;31(9):980–97. doi: 10.1038/s41422-021-00506-9 (PMC8410789; doi:10.1038/s41422-021-00506-9)
Supplement: Supplementary file 3 — Fig S3 [file 41422_2021_506_MOESM3_ESM.pdf]

Supplementary information, Fig. S3. In this figure, cancer cells were treated with DM-αKG (15 mM) for 24 hours to assess DR6 oxidation and pyroptotic features (including morphology, GSDMC cleavage, and LDH release), unless specially indicated otherwise.

(**a**) Inhibitors, Genistein or MβCD, showed no effect on DM-αKG-induced DR6 oxidation. HeLa cells were pretreated with Genistein and MβCD for 2 hours.

(**b**) The knockdown efficiency of CLTA, CLTB, CLAC, DNM1 and DNM2 in HeLa cells as determined by RT-qPCR.

(**c, d, e**) Effect of CLTs on DM-αKG-induced caspase-8 activation (d), pyroptotic morphology (d), GSDMC cleavage and LDH release (e). CLTA, CLTB, and CLAC had first been knocked down in HeLa cells.

(**f, g, h**) Effect of DNMs on DM-αKG-induced pyroptotic morphology (f), caspase-8 activation (g), GSDMC cleavage and LDH release (h). DNM1 and DNM2 had first been knocked down in the cells.

(**i**) Genistein or MβCD impaired DM-αKG-induced GSDMC cleavage in SGC-7901 (top) and B16 (bottom) cells.

Tubulin was used to determine the amount of loading proteins. All data are presented as the mean±SEM of two or three independent experiments. *** $p<0.001$. The data

were analyzed using two-way ANOVA followed by the Bonferroni test.

Supplementary information, Figure S3

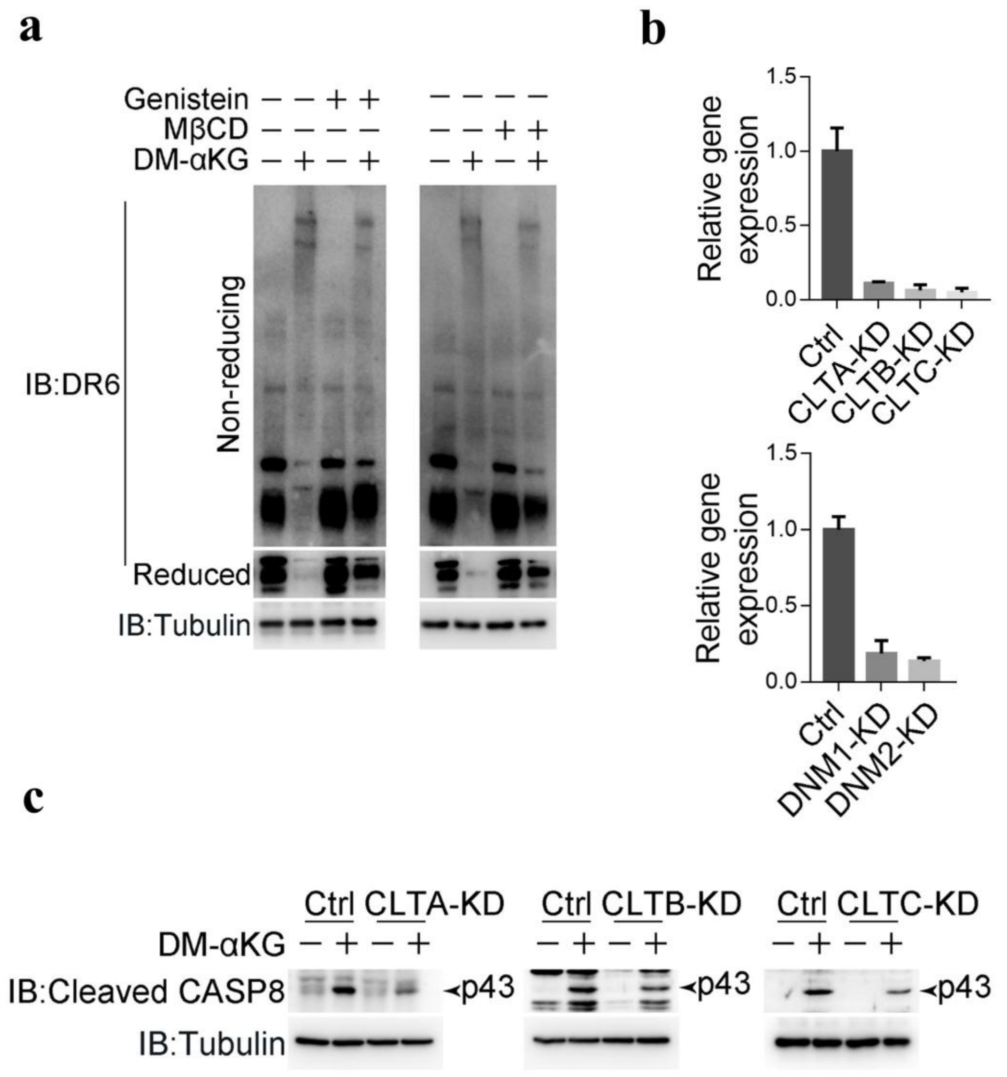

d

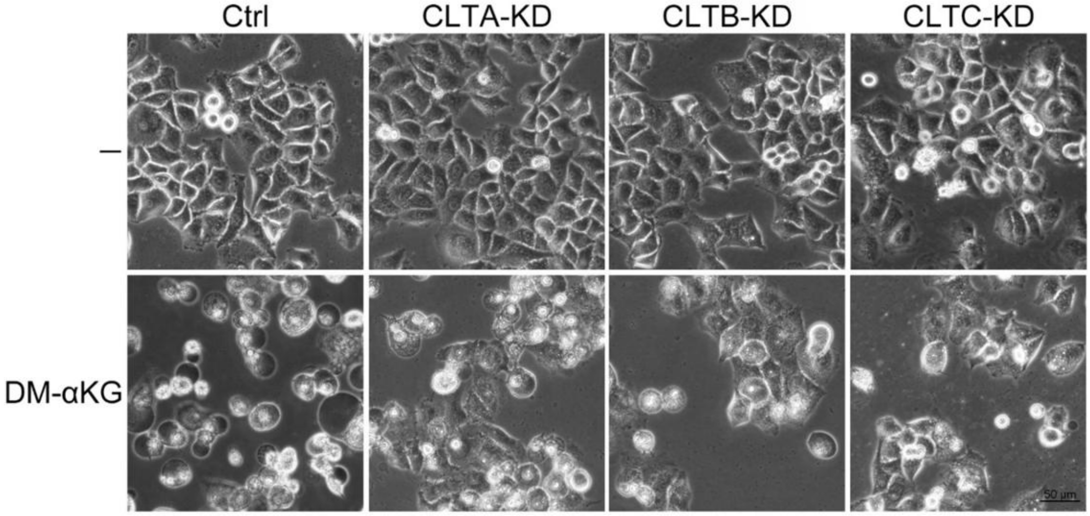
Ctrl
CLTA-KD
CLTB-KD
CLTC-KD
—
DM-αKG
50 µm

e

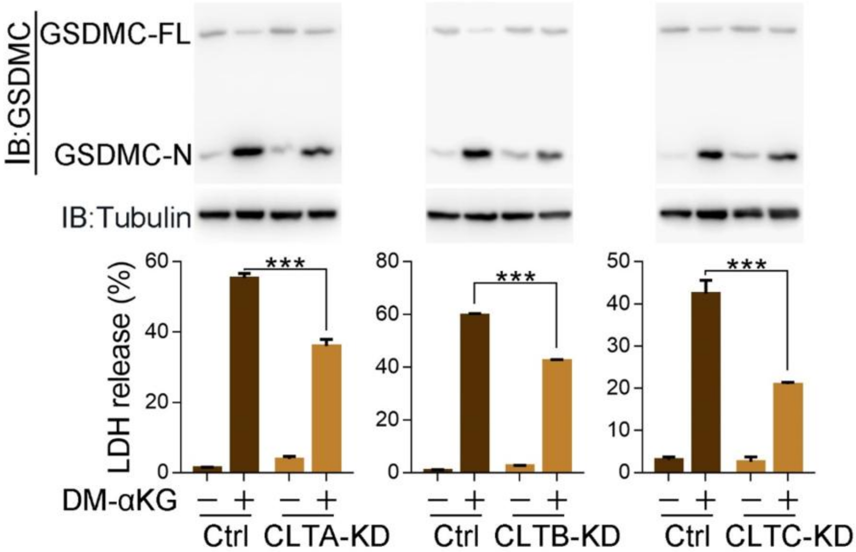
IB:GSDMC
GSDMC-FL
GSDMC-N
IB:Tubulin
LDH release (%)
0
20
40
60
80
0
10
20
30
40
50
***
DM-αKG
— + — +
Ctrl
CLTA-KD
CLTB-KD
CLTC-KD

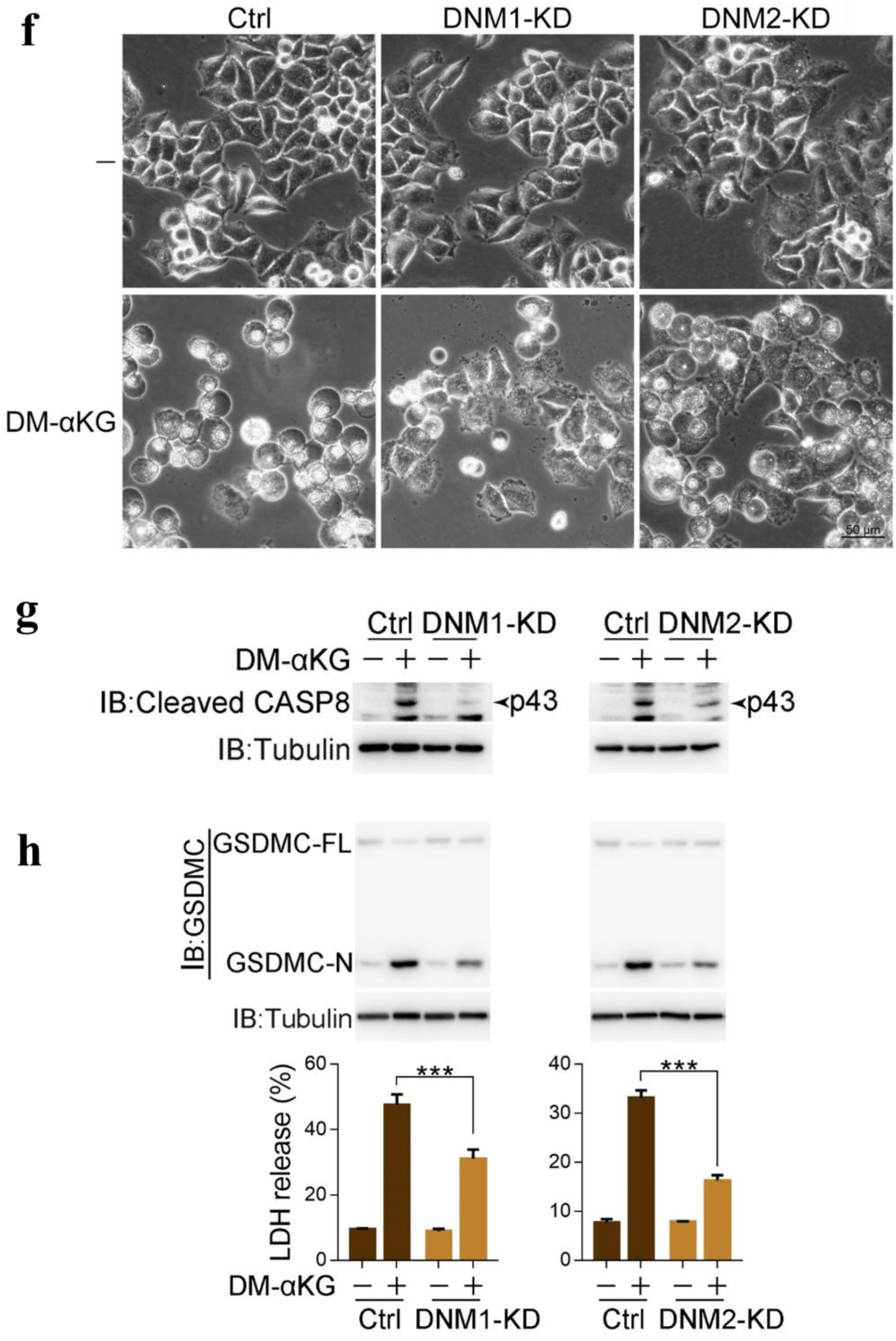
f
Ctrl
DNM1-KD
DNM2-KD
DM-αKG
50 μm
g
Ctrl
DNM1-KD
Ctrl
DNM2-KD
DM-αKG
IB:Cleaved CASP8
p43
IB:Tubulin
h
IB:GSDMC
GSDMC-FL
GSDMC-N
IB:Tubulin
LDH release (%)
***
***
DM-αKG

i

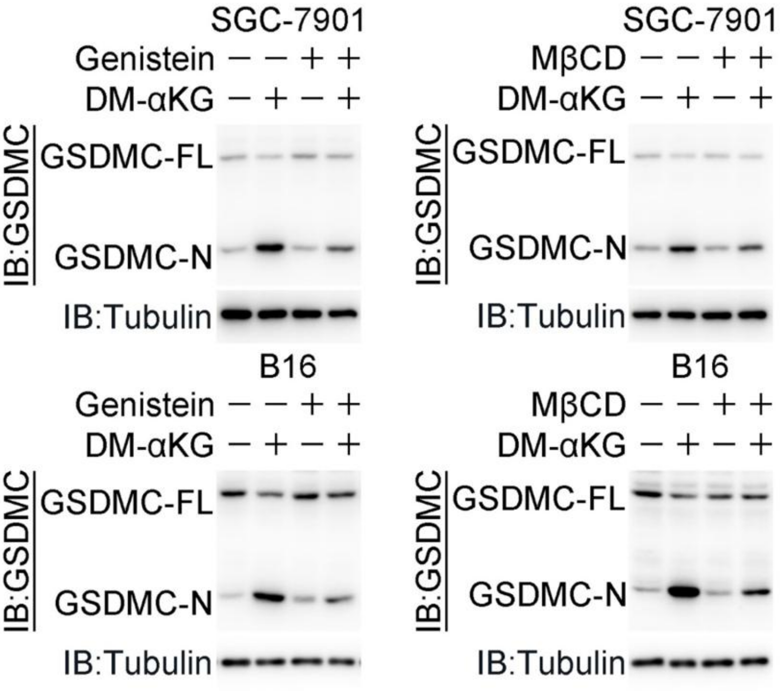
SGC-7901
Genistein − − + +
DM-αKG − + − +
IB:GSDMC
GSDMC-FL
GSDMC-N
IB:Tubulin
SGC-7901
MβCD − − + +
DM-αKG − + − +
IB:GSDMC
GSDMC-FL
GSDMC-N
IB:Tubulin
B16
Genistein − − + +
DM-αKG − + − +
IB:GSDMC
GSDMC-FL
GSDMC-N
IB:Tubulin
B16
MβCD − − + +
DM-αKG − + − +
IB:GSDMC
GSDMC-FL
GSDMC-N
IB:Tubulin
