# Supplementary material for: The metabolite α-KG induces GSDMC-dependent pyroptosis through death receptor 6-activated caspase-8
Source: Cell Res. 2021 May 19;31(9):980–97. doi: 10.1038/s41422-021-00506-9 (PMC8410789; doi:10.1038/s41422-021-00506-9)
Supplement: Supplementary file 4 — Fig S4 [file 41422_2021_506_MOESM4_ESM.pdf]

Supplementary information, Fig. S4. In this figure, HeLa cells were treated with DM-αKG (15 mM) for 6 hours to observe the protein localization under confocal microscopy, or 24 hours to assess DR6 oxidation and pyroptotic features (including cell morphology, GSDMC cleavage, and LDH release), unless specially indicated otherwise.

(**a, b**) DM-αKG induced DR6 colocalization with either caspase-8 (a) or GSDMC (b) as observed under confocal microscopy. Cells were transfected with different plasmids as indicated.

(**c**) Knocking down caspase-8 (left) or GSDMC (right) had no effect on DM-αKG-induced DR6 puncta formation. DR6-GFP was transfected into caspase-8- or GSDMC-knockdown cells.

(**d**) Co-IP assay indicated the interaction between DR6-caspase-8 (left) and DR6-GSDMC (right). Different plasmids were transfected into cells as indicated.

(**e**) The knockdown efficiency of FADD and TRADD in the cells as determined by western blotting.

(**f**) Knocking down TRADD had no effect on DM-αKG induced pyroptosis, including cell morphology and LDH release.

(**g**) Genistein impaired the DM-αKG-induced plasma membrane localization of

GSDMC-N.

(h) TNFα/CHX but not DM-αKG induced the cleavage of Bid and caspase-3.

Tubulin was used to determine the amount of loading proteins. All data are presented as the mean±SEM of two or three independent experiments. ns: not significant. The data were analyzed two-way ANOVA followed by the Bonferroni test.

Supplementary information, Figure S4

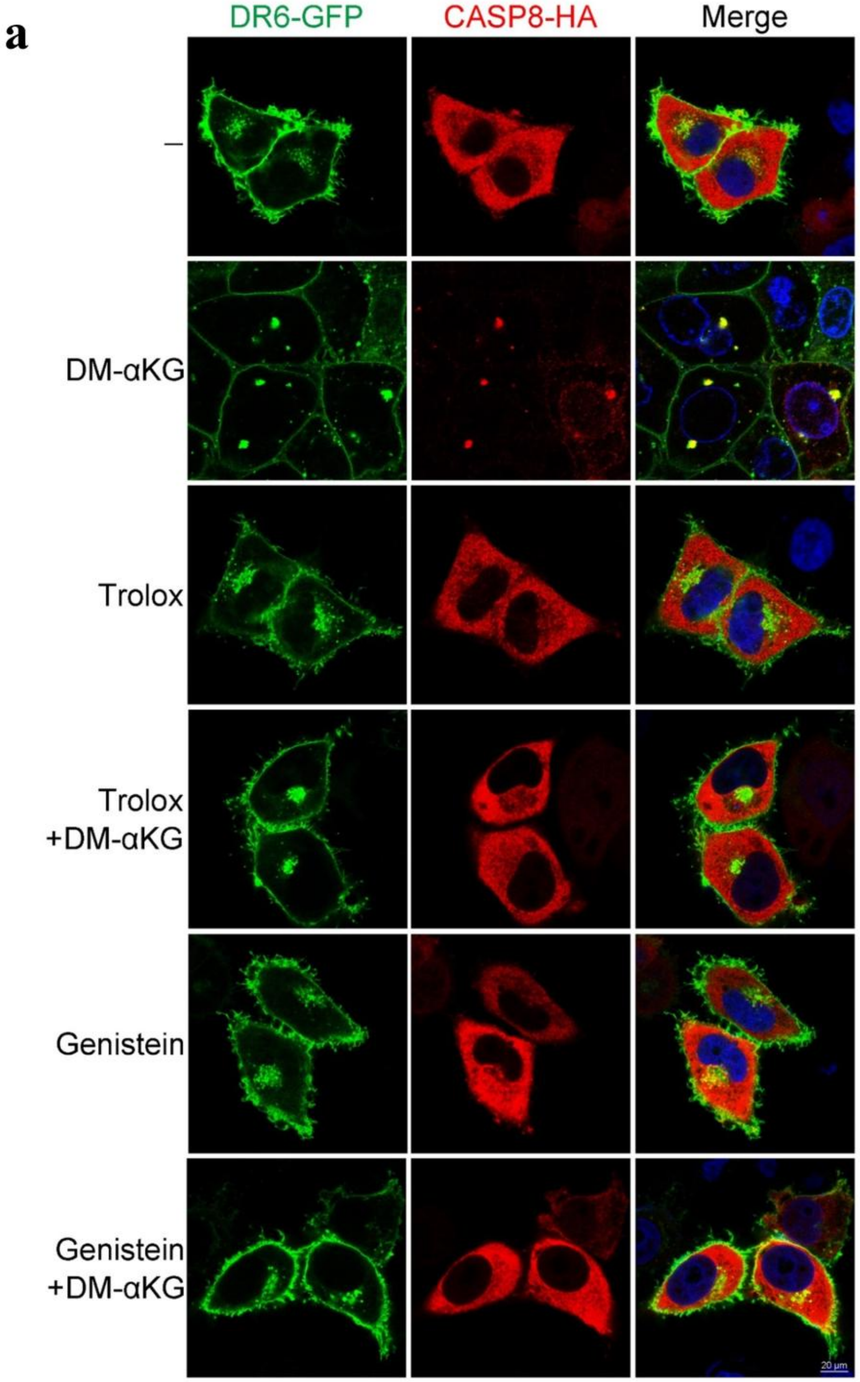

b

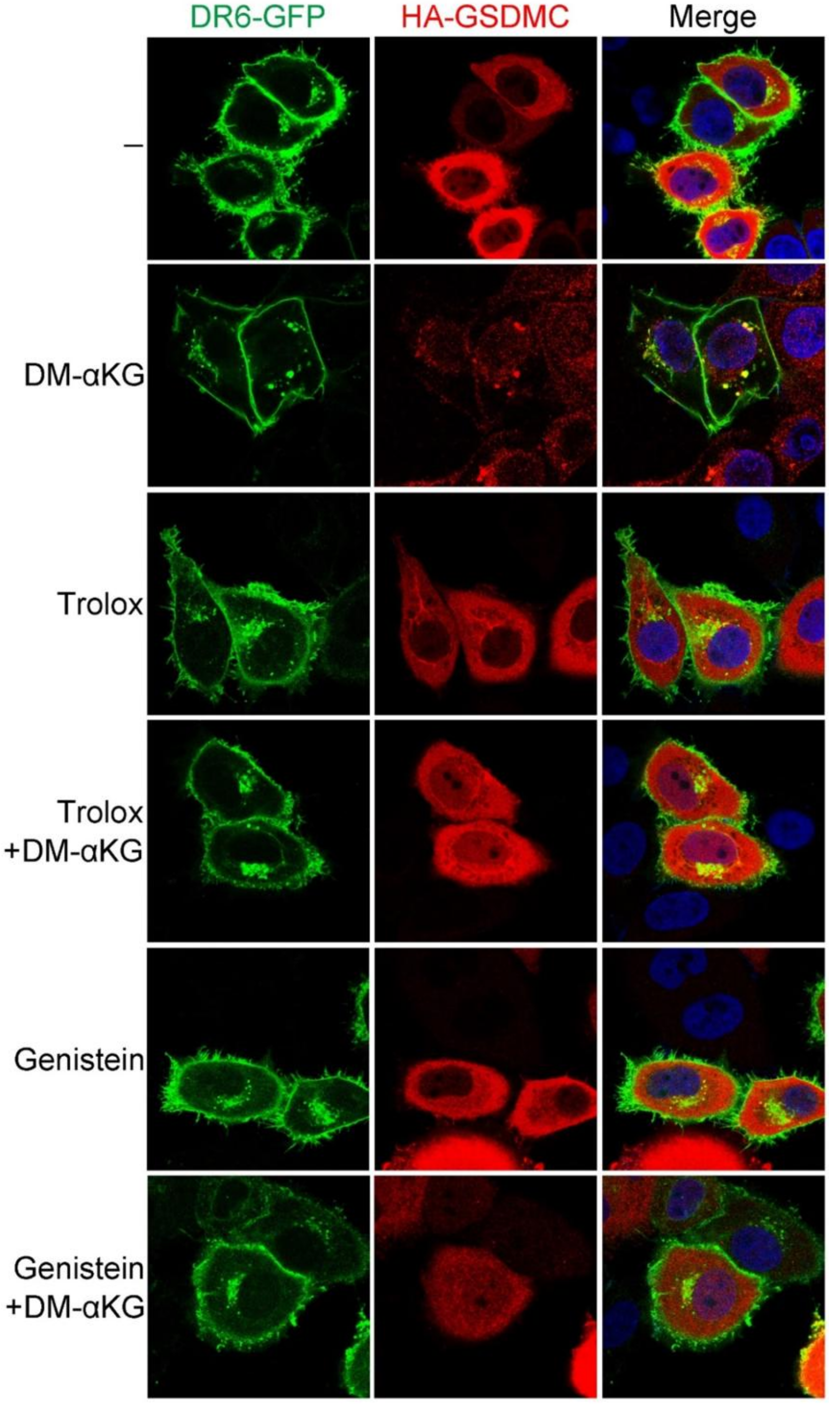
DR6-GFP
HA-GSDMC
Merge
–
DM-αKG
Trolox
Trolox
+DM-αKG
Genistein
Genistein
+DM-αKG

c

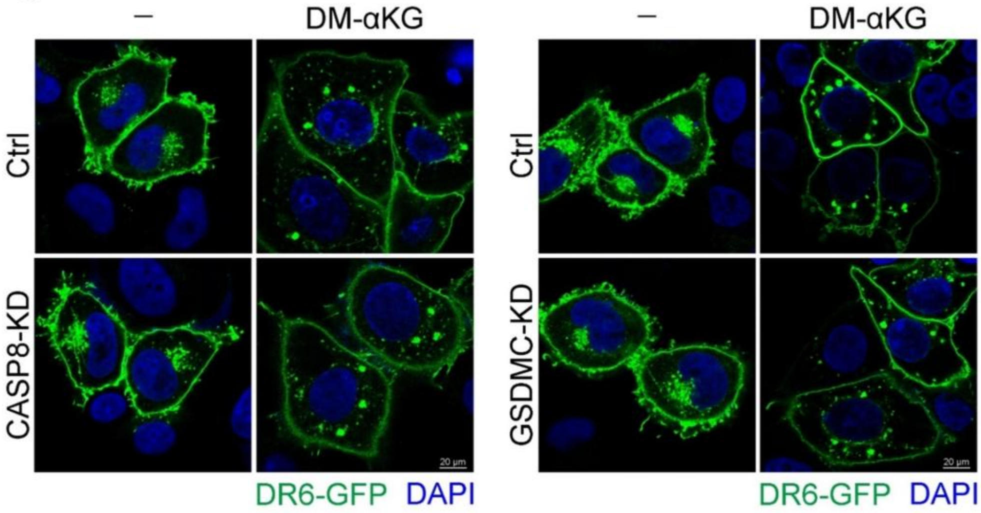

–
DM-αKG
Ctrl
CASP8-KD
DR6-GFP DAPI
–
DM-αKG
Ctrl
GSDMC-KD
DR6-GFP DAPI

d

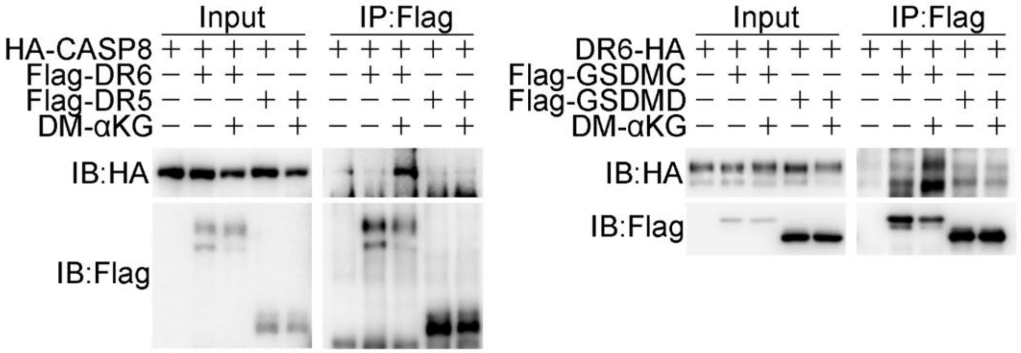

Input
IP:Flag
HA-CASP8
Flag-DR6
Flag-DR5
DM-αKG
IB:HA
IB:Flag
Input
IP:Flag
DR6-HA
Flag-GSDMC
Flag-GSDMD
DM-αKG
IB:HA
IB:Flag

e

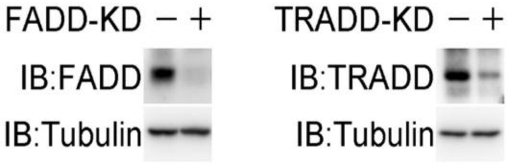

FADD-KD – +
IB:FADD
IB:Tubulin
TRADD-KD – +
IB:TRADD
IB:Tubulin

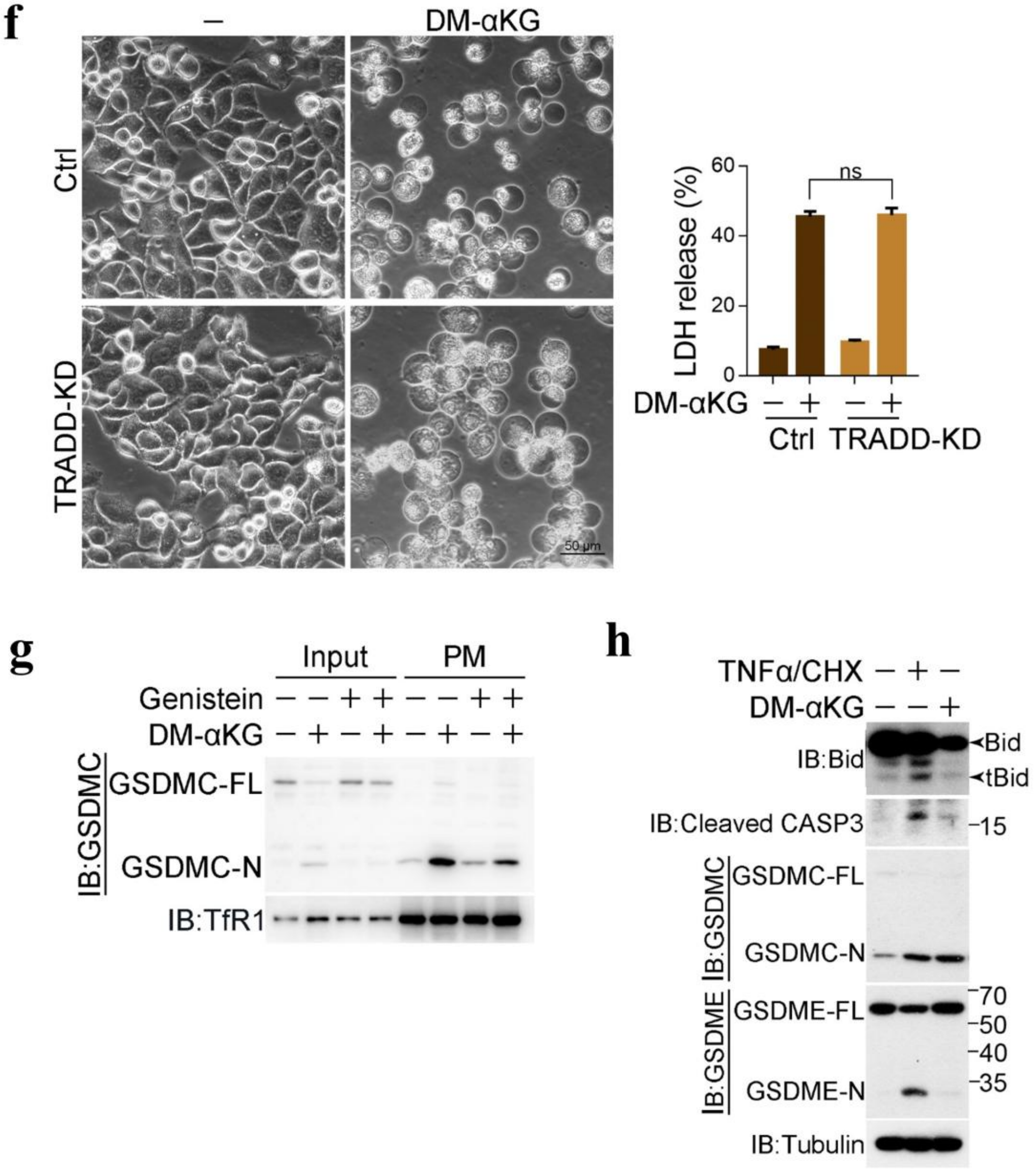
f
–
DM-αKG
Ctrl
TRADD-KD
50 μm
LDH release (%)
60
40
20
0
ns
DM-αKG – + – +
Ctrl
TRADD-KD
g
Input
PM
Genistein – – + + – – + +
DM-αKG – + – + – + – +
IB:GSDMC
GSDMC-FL
GSDMC-N
IB:TfR1
h
TNFα/CHX – + –
DM-αKG – – +
IB:Bid
Bid
tBid
IB:Cleaved CASP3
15
IB:GSDMC
GSDMC-FL
GSDMC-N
IB:GSDME
GSDME-FL
GSDME-N
70
50
40
35
IB:Tubulin
