# Supplementary material for: The metabolite α-KG induces GSDMC-dependent pyroptosis through death receptor 6-activated caspase-8
Source: Cell Res. 2021 May 19;31(9):980–97. doi: 10.1038/s41422-021-00506-9 (PMC8410789; doi:10.1038/s41422-021-00506-9)
Supplement: Supplementary file 5 — Fig S5 [file 41422_2021_506_MOESM5_ESM.pdf]

Supplementary information, Fig. S5.

(**a, b**) Effects of knocking down DR6 (a) or GSDMC (b) on tumor size and weight. DR6 or GSDMC was knocked down in HeLa cells, which were then injected subcutaneously into the posterior flanks of nude mice (n=8). The mice were treated with DM-αKG (500 mg/kg) every other day for 2 weeks. Images of xenograft tumors in the nude mice and tumor weights are shown. PBS was used as a control.

(**c**) The cleavage of GSDMC was determined in tumor samples obtained from (a).

(**d, e**) Comparison of the effects of $DR6^{WT}$ and $DR6^{5CS}$ on tumor growth (d) and GSDMC cleavage (e). DR6 was knocked down first in HeLa cells, and then $DR6^{WT}$ and $DR6^{5CS}$ were transfected into cells. Other treatment was the same as (a) and (c).

(f) Comparison of $GSDMC^{WT}$ and $GSDMC^{D240A}$ on tumor growth. GSDMC was knocked down first in HeLa cells, and then $GSDMC^{WT}$ and $GSDMC^{D240A}$ were transfected into cells. Other treatment was the same as (a).

(g, h, i) Administration of DM-αKG showed no side effects in the normal C57BL/6J mice. Mice were intraperitoneally injected with DM-αKG (500 mg/kg) or PBS control once per day for 7 days. Mouse body weights (g), images of mouse colon (h) and spleen (i) with colon lengths and spleen weights were indicated.

Tubulin was used to determine the amount of loading proteins. All data are presented as the mean±SEM of two or three independent experiments. ** $p<0.01$, *** $p<0.001$, ns: not significant. The data were analyzed using two-tailed Student's t-test in (g, h, i) or

two-way ANOVA followed by the Bonferroni test in (a, b, d, f).

Supplementary information, Figure S5

a

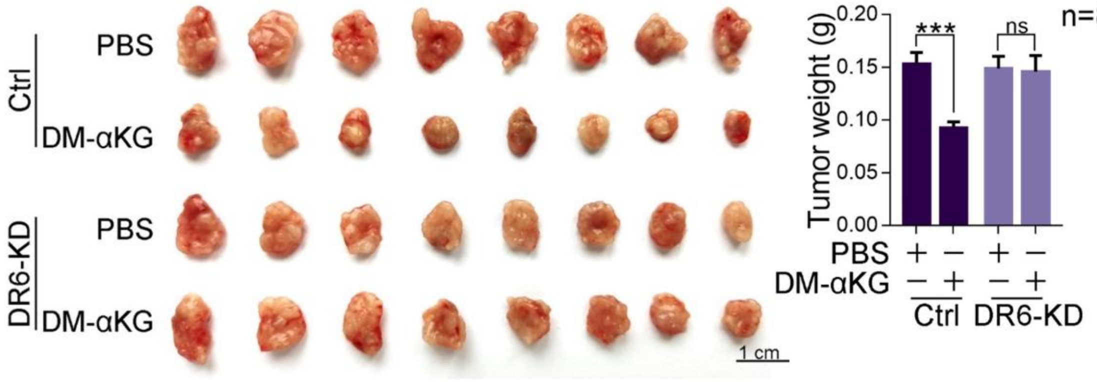

b

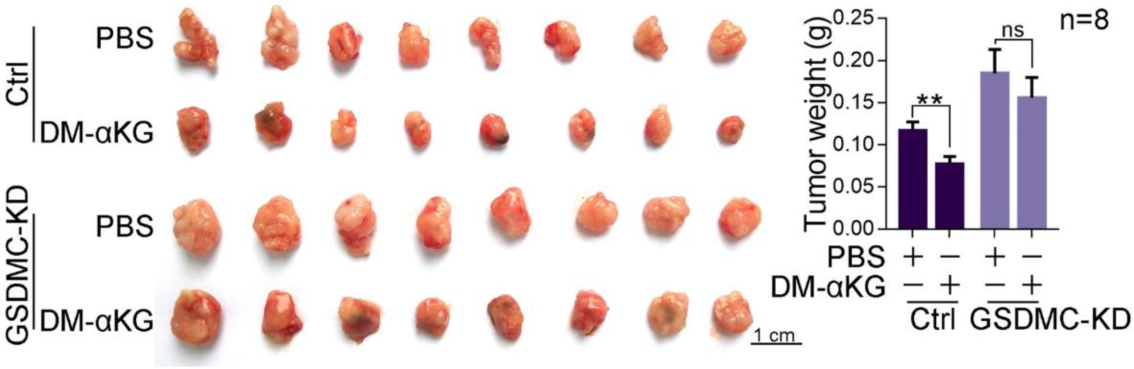

c

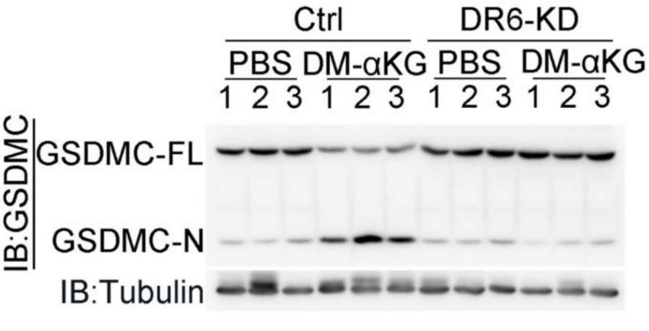

d

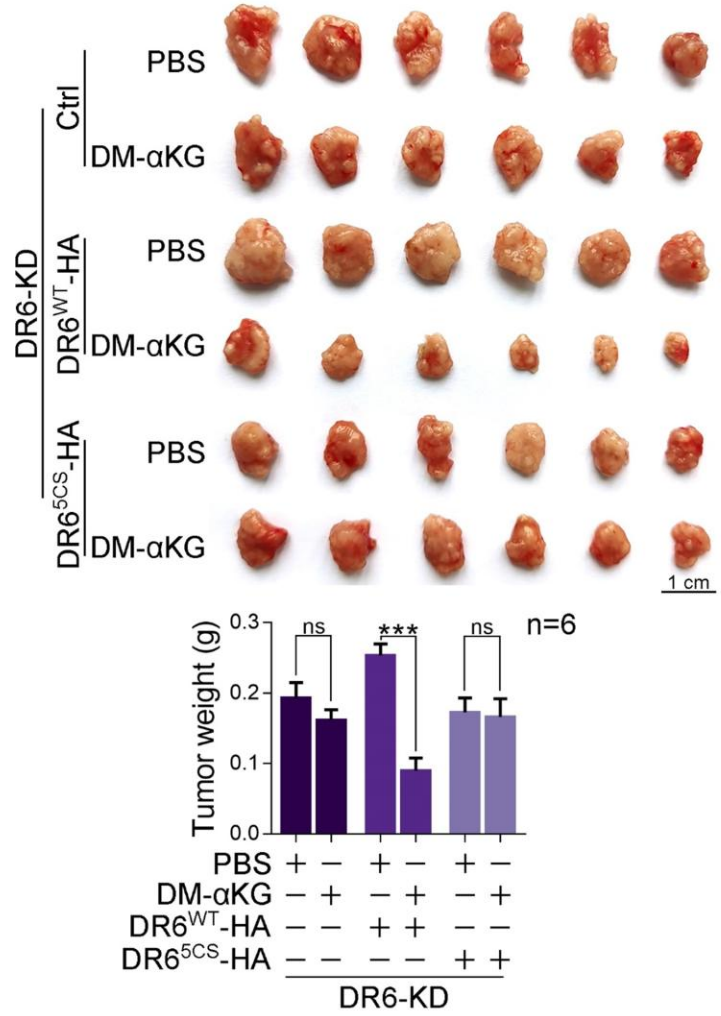
DR6-KD
Ctrl
DR6^{WT}-HA
DR6^{5CS}-HA
PBS
DM-αKG
PBS
DM-αKG
PBS
DM-αKG
1 cm
Tumor weight (g)
0.0
0.1
0.2
0.3
ns
***
ns
n=6
PBS
DM-αKG
DR6^{WT}-HA
DR6^{5CS}-HA
DR6-KD

e

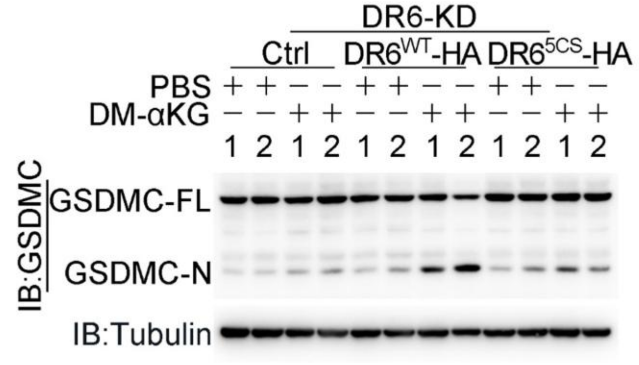
DR6-KD
Ctrl
DR6^{WT}-HA
DR6^{5CS}-HA
PBS
DM-αKG
1 2 1 2 1 2 1 2 1 2 1 2
IB:GSDMC
GSDMC-FL
GSDMC-N
IB:Tubulin

f

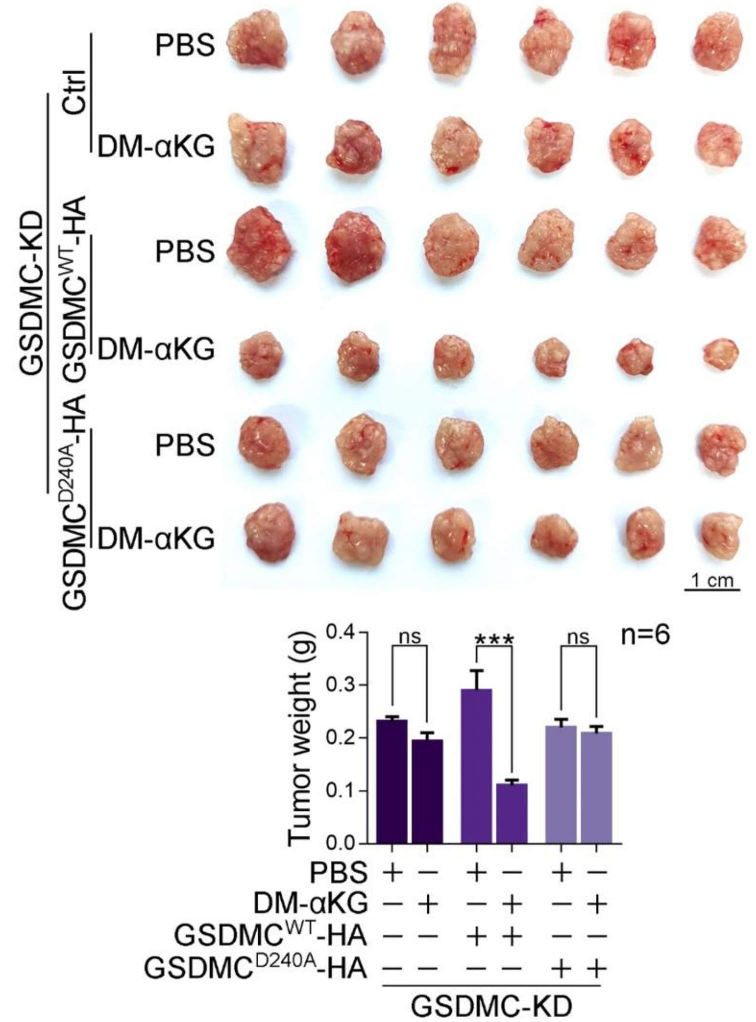

GSDMC-KD
Ctrl
GSDMC^{WT}-HA
GSDMC^{D240A}-HA
PBS
DM-αKG
PBS
DM-αKG
PBS
DM-αKG
1 cm
Tumor weight (g)
0.4
0.3
0.2
0.1
0.0
ns
***
ns
n=6
PBS + − + − + −
DM-αKG − + − + − +
GSDMC^{WT}-HA − − + + − −
GSDMC^{D240A}-HA − − − − + +
GSDMC-KD

g

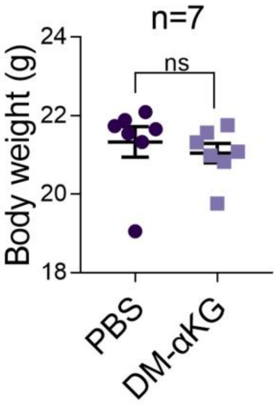
n=7
ns
Body weight (g)
24
22
20
18
PBS
DM-αKG

h

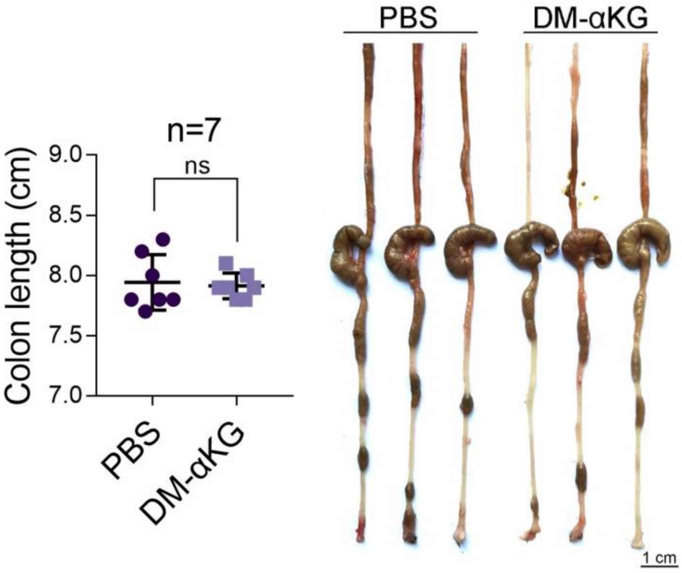
n=7
ns
Colon length (cm)
9.0
8.5
8.0
7.5
7.0
PBS
DM-αKG
PBS
DM-αKG
1 cm

i

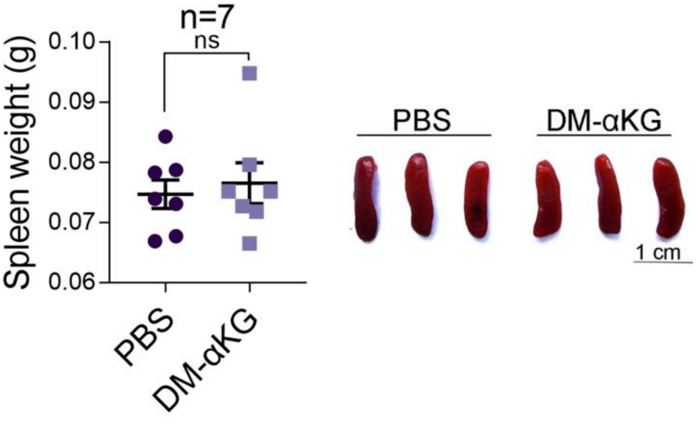
n=7
ns
Spleen weight (g)
0.10
0.09
0.08
0.07
0.06
PBS
DM-αKG
PBS
DM-αKG
1 cm
