# Supplementary material for: The metabolite α-KG induces GSDMC-dependent pyroptosis through death receptor 6-activated caspase-8
Source: Cell Res. 2021 May 19;31(9):980–97. doi: 10.1038/s41422-021-00506-9 (PMC8410789; doi:10.1038/s41422-021-00506-9)
Supplement: Supplementary file 6 — Fig S6 [file 41422_2021_506_MOESM6_ESM.pdf]

Supplementary information, Fig. S6. In this figure, cancer cells were treated with DM-αKG (15 mM) for 6 hours to determine the ROS level, or 24 hours to assess pyroptotic features (including cell morphology, GSDMC cleavage, LDH release, and $Annv^+/PI^+$ cells), unless specially indicated otherwise.

(**a**) DM-αKG-induced ROS level and pyroptosis were not influenced by dimethyloxalylglycine (DMOG). HeLa cells were pretreated with DMOG (50 μM) for 2 hours before DM-αKG treatment.

(b) The knockdown efficiency of OGDH, IDH1, IDH2, MDH1, MDH2 and LDHA in HeLa cells, as determined by western blot or RT-qPCR analysis.

(**c, d**) Knocking down IDH1 or IDH2 showed no effects on DM-αKG-induced pyroptotic morphology, LDH release (c), or ROS levels (d) in HeLa cells.

(**e, f, g, h**) Knocking down OGDH increased ROS levels (e), GSDMC cleavage and LDH release (f), $Annv^+/PI^+$ cells (g) and pyroptotic morphology (h) as induced by DM-αKG. HeLa cells were treated with DM-αKG with or without OGDH knocked down.

(**i**) Knocking down LDHA had no effect on DM-αKG-induced ROS levels or pyroptotic morphology in HeLa cells.

(**j**) Knocking down MDH2 had no effect on DM-αKG-induced ROS levels or pyroptosis in HeLa cells, as determined by cell morphology and LDH release.

(**k**) Knocking down MDH1 impaired DM-αKG-induced pyroptosis in SGC-7901 (top) and B16 (bottom) cells, as determined by cell morphology, GSDMC cleavage and LDH release.

(**l**) The expression levels of $MDH1^{WT}$ and $MDH1^{H187Y}$ in HeLa cells.

(**m, n, o**) Knocking down caspase-8 (m), DR6 (n) or GSDMC (o) impaired Octyl-L-2HG-induced pyroptosis in HeLa cells, as determined by cell morphology and LDH release.

(p, q) Knocking out DR6 (p) and GSDMC (q) abolished hypoxia-induced pyroptosis in HeLa cells.

Tubulin was used to determine the amount of loading proteins. All data are presented as the mean±SEM of two or three independent experiments. *** $p<0.001$, ns: not significant. The data were analyzed using two-way ANOVA followed by the Bonferroni test.

Supplementary information, Figure S6

a

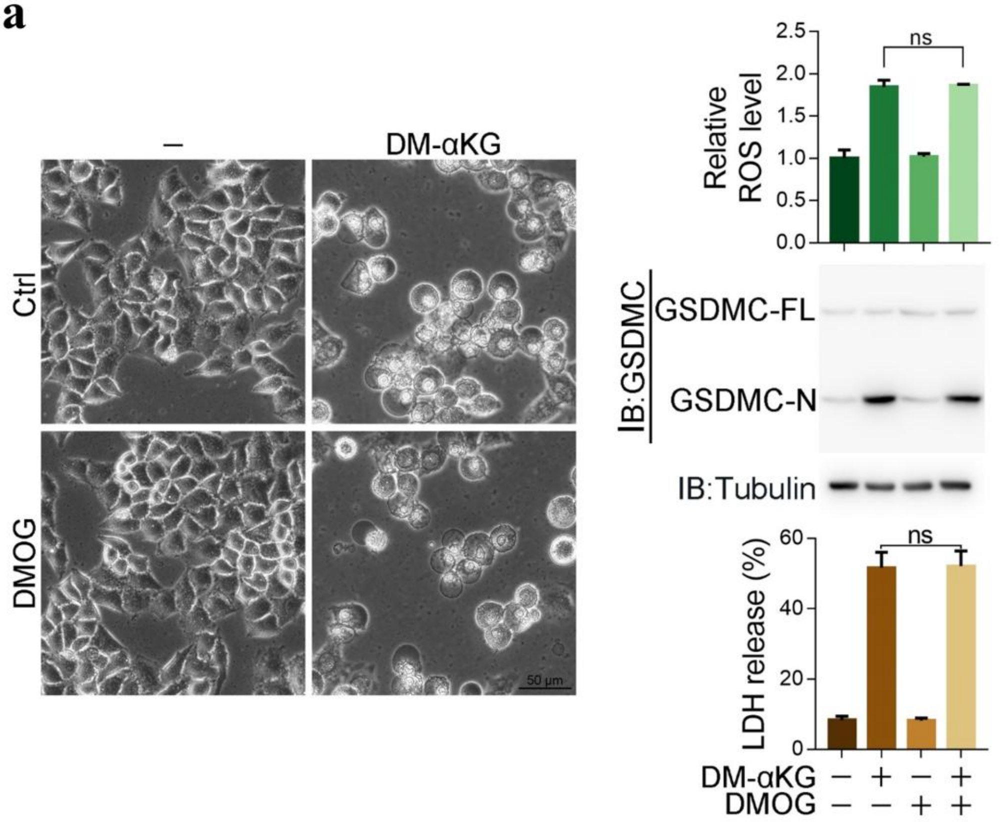

b

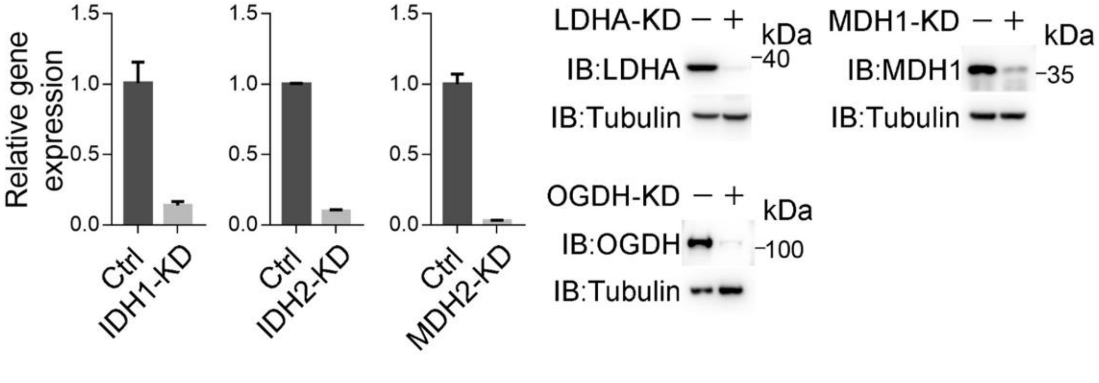

c

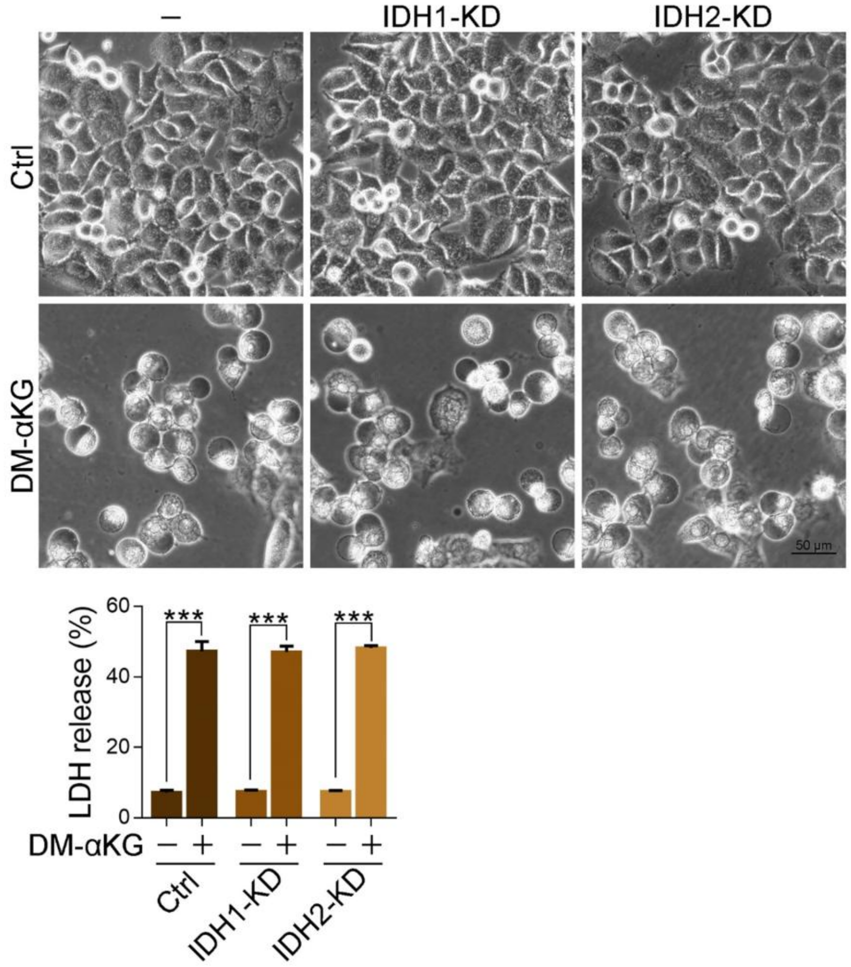

–
IDH1-KD
IDH2-KD
Ctrl
DM-αKG
50 μm
LDH release (%)
60
40
20
0
***
***
***
DM-αKG – + – + – +
Ctrl
IDH1-KD
IDH2-KD

d

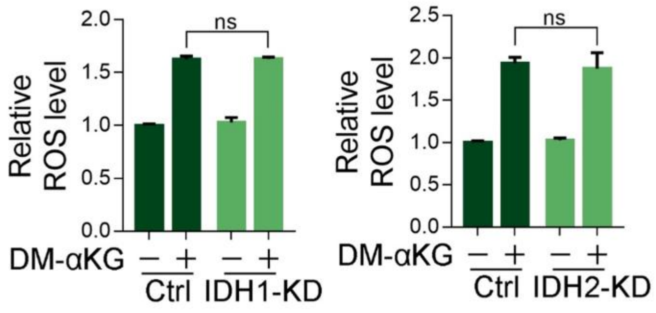

Relative ROS level
2.0
1.5
1.0
0.5
0.0
ns
DM-αKG – + – +
Ctrl IDH1-KD
Relative ROS level
2.5
2.0
1.5
1.0
0.5
0.0
ns
DM-αKG – + – +
Ctrl IDH2-KD

e

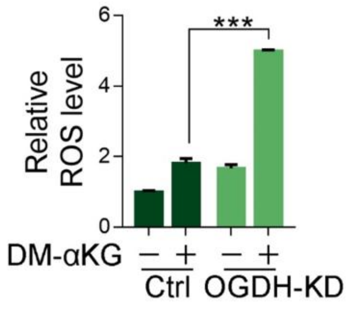

Relative ROS level
6
4
2
0
***
DM-αKG – + – +
Ctrl OGDH-KD

f

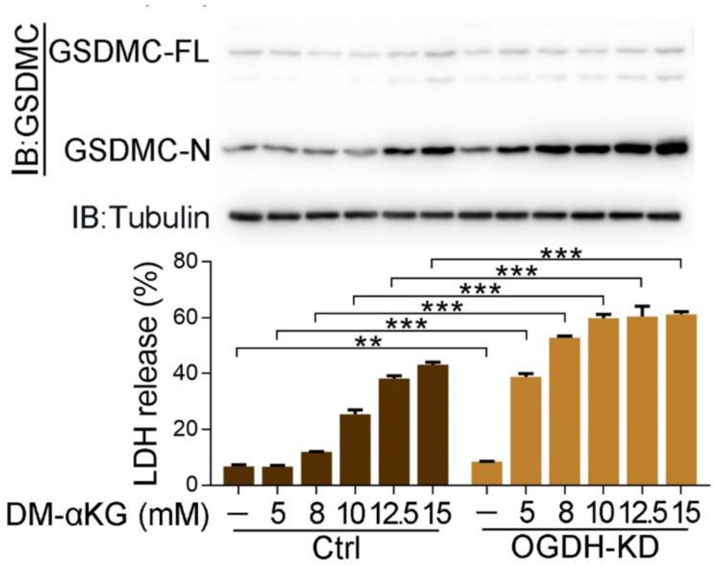
IB:GSDMC
GSDMC-FL
GSDMC-N
IB:Tubulin
LDH release (%)
80
60
40
20
0
**
***
***
***
***
DM-αKG (mM)
— 5 8 10 12.5 15
— 5 8 10 12.5 15
Ctrl
OGDH-KD

g

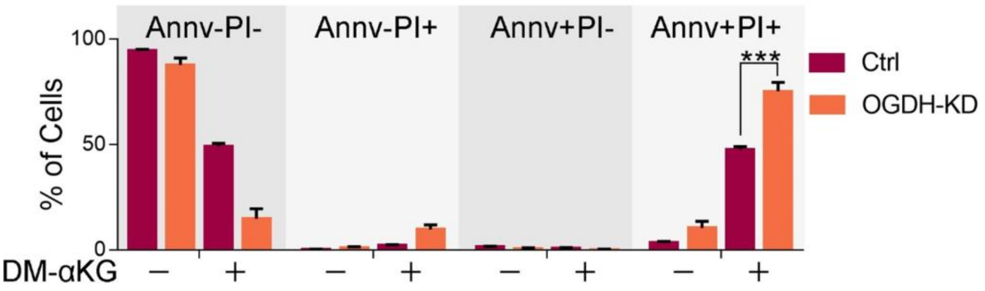
Annv-PI-
Annv-PI+
Annv+PI-
Annv+PI+
% of Cells
100
50
0
***
Ctrl
OGDH-KD
DM-αKG
— + — + — + — +

h

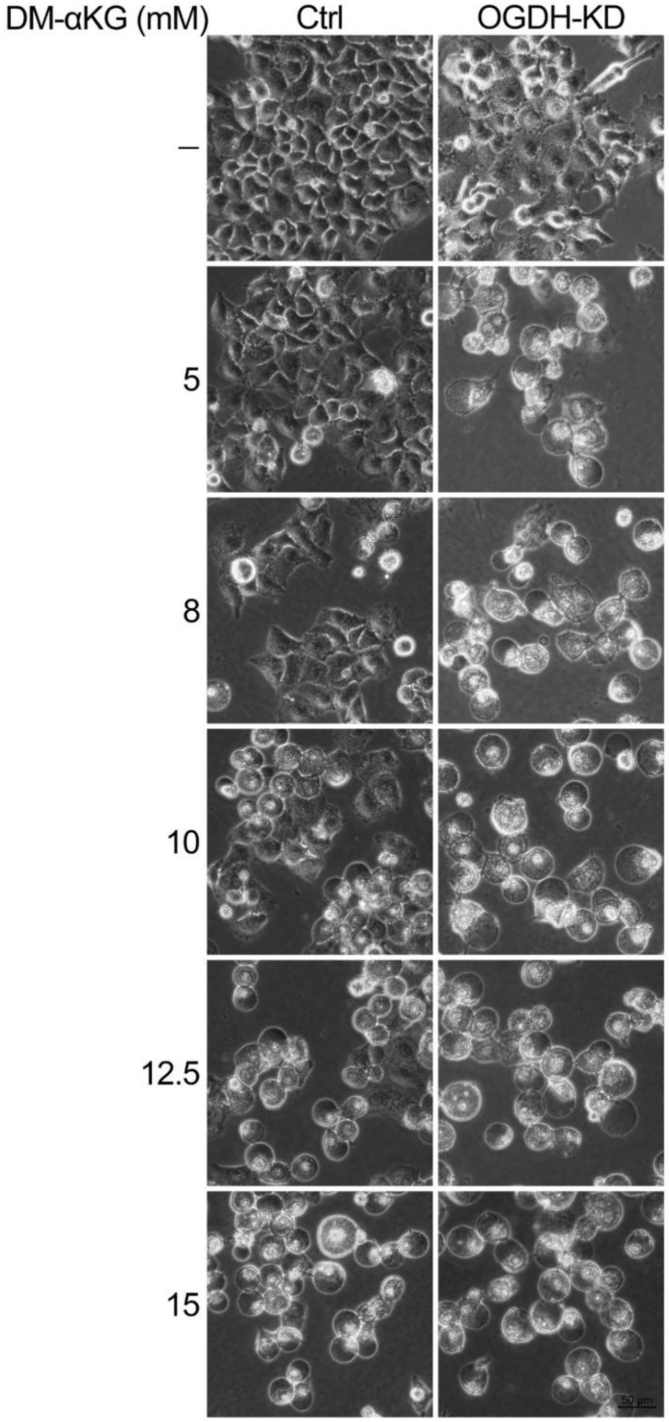

i

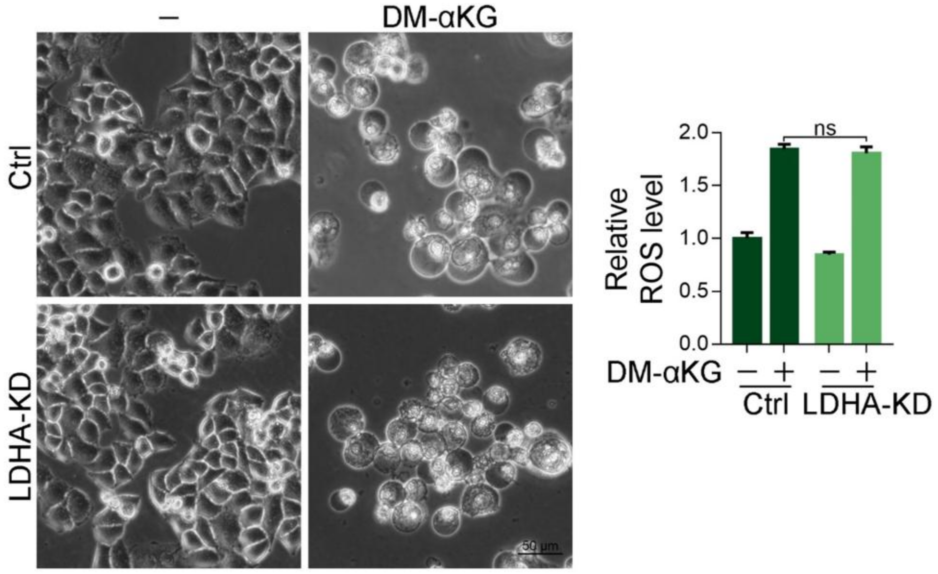
–
DM-αKG
Ctrl
LDHA-KD
50 μm
ns
2.0
1.5
1.0
0.5
0.0
Relative ROS level
DM-αKG
– + – +
Ctrl
LDHA-KD

j

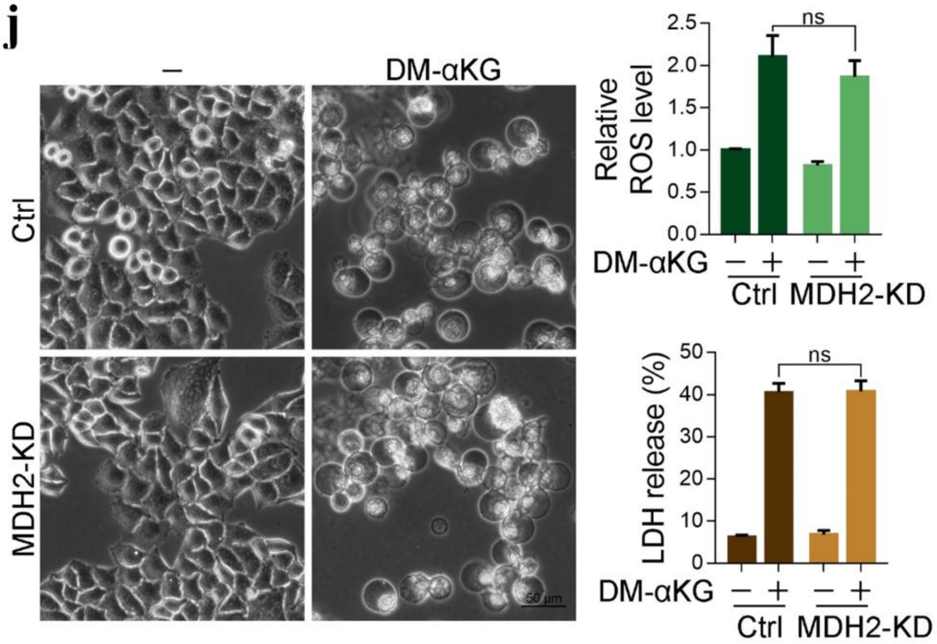
–
DM-αKG
Ctrl
MDH2-KD
50 μm
ns
2.5
2.0
1.5
1.0
0.5
0.0
Relative ROS level
DM-αKG
– + – +
Ctrl
MDH2-KD
ns
50
40
30
20
10
0
LDH release (%)
DM-αKG
– + – +
Ctrl
MDH2-KD

k

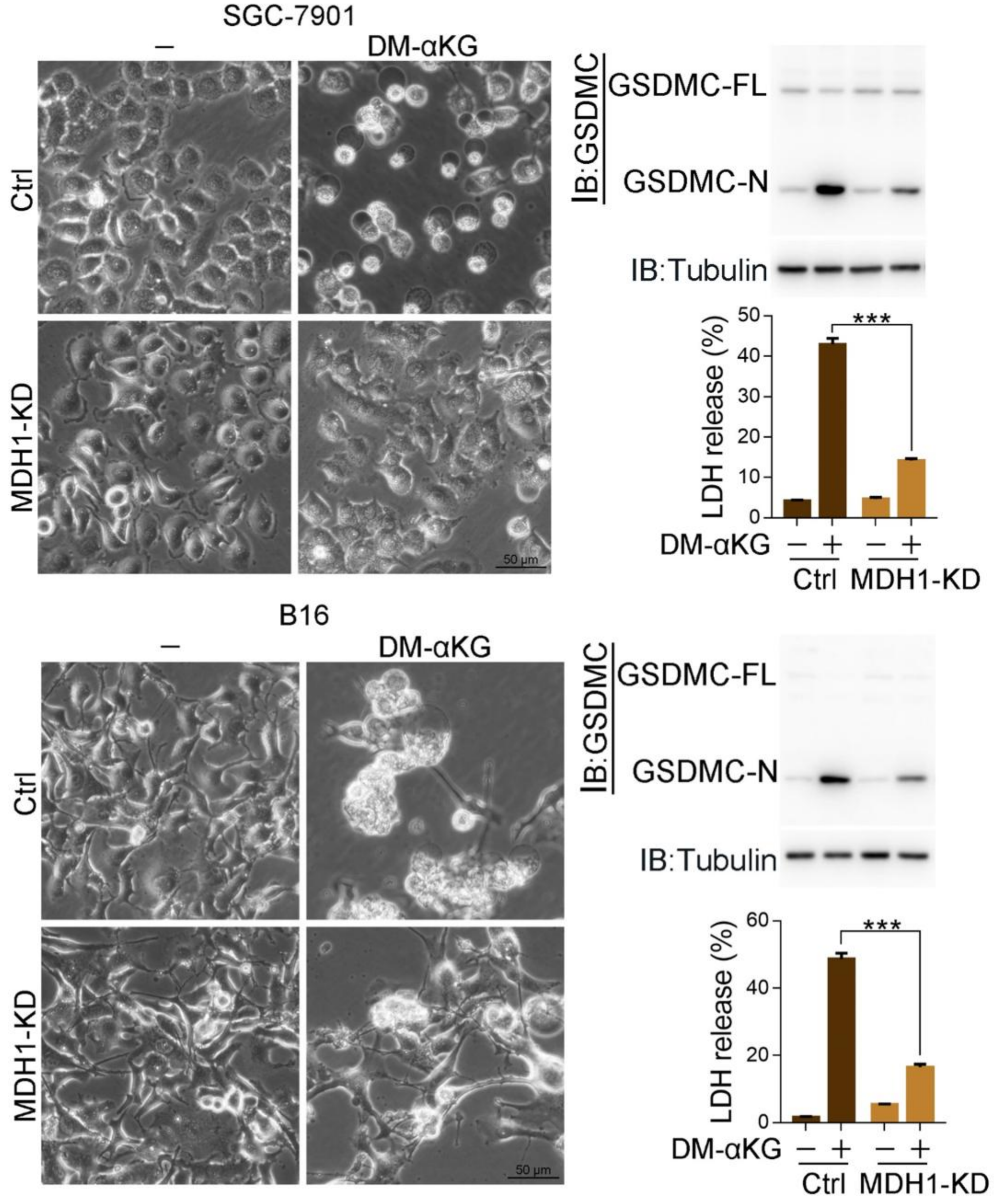

SGC-7901
–
DM-αKG
Ctrl
MDH1-KD
50 μm
IB:GSDMC
GSDMC-FL
GSDMC-N
IB:Tubulin
LDH release (%)
0
10
20
30
40
50
***
DM-αKG – + – +
Ctrl
MDH1-KD
B16
–
DM-αKG
Ctrl
MDH1-KD
50 μm
IB:GSDMC
GSDMC-FL
GSDMC-N
IB:Tubulin
LDH release (%)
0
20
40
60
***
DM-αKG – + – +
Ctrl
MDH1-KD

l

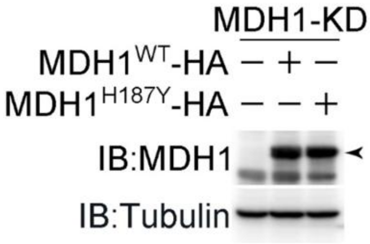
MDH1-KD
MDH1WT-HA − + −
MDH1H187Y-HA − − +
IB:MDH1
IB:Tubulin

m

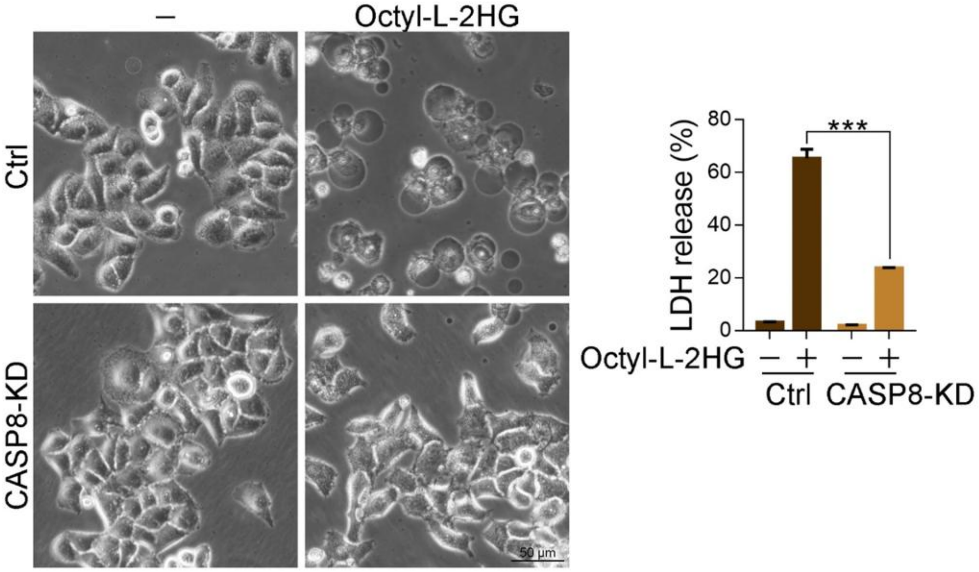
−
Octyl-L-2HG
Ctrl
CASP8-KD
50 µm
LDH release (%)
80
60
40
20
0
***
Octyl-L-2HG − + − +
Ctrl
CASP8-KD

n

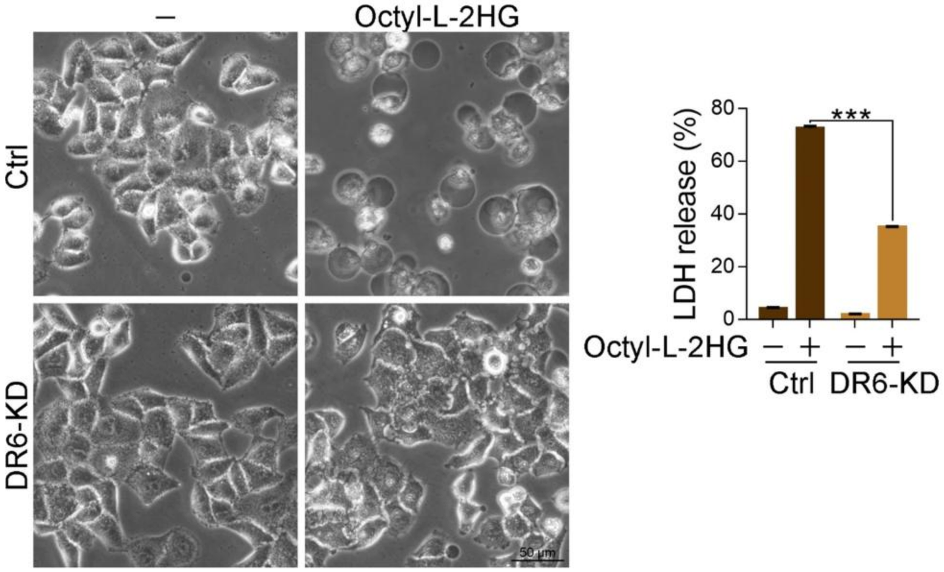
−
Octyl-L-2HG
Ctrl
DR6-KD
50 µm
LDH release (%)
80
60
40
20
0
***
Octyl-L-2HG − + − +
Ctrl
DR6-KD

o

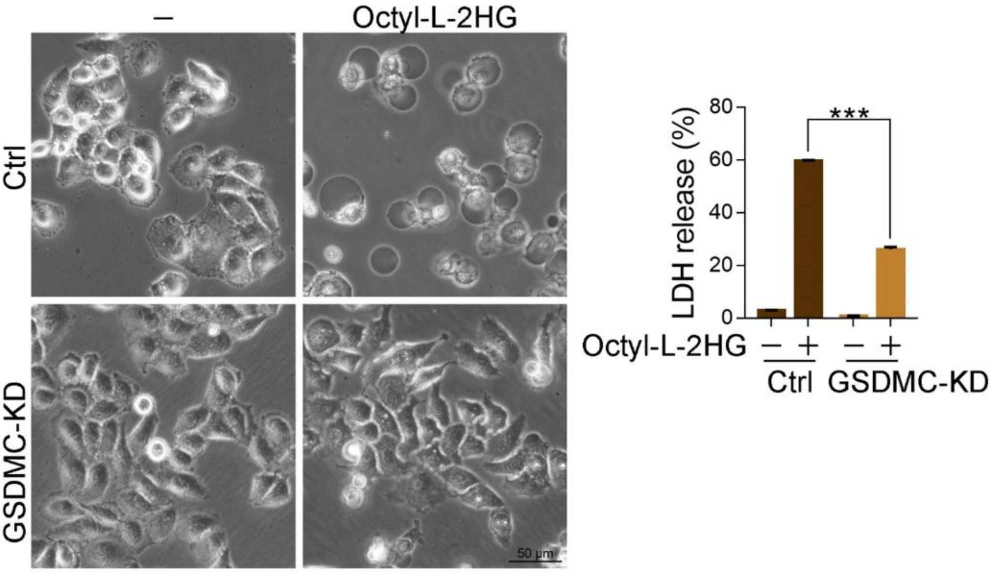
–
Octyl-L-2HG
Ctrl
GSDMC-KD
50 μm
LDH release (%)
80
60
40
20
0
***
Octyl-L-2HG – + – +
Ctrl
GSDMC-KD

p

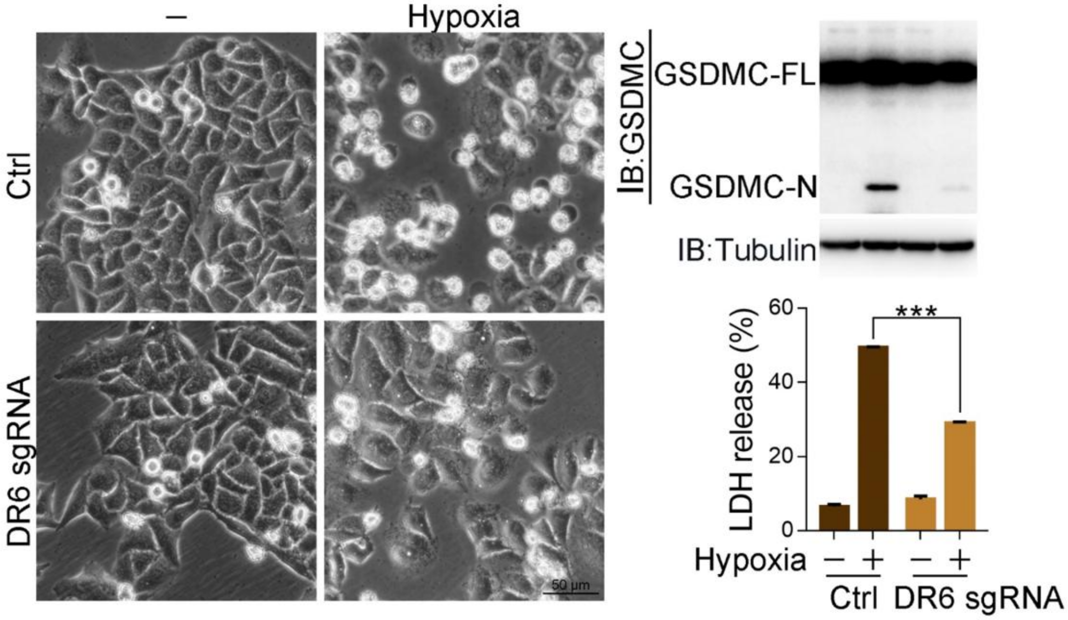
–
Hypoxia
Ctrl
DR6 sgRNA
50 μm
IB:GSDMC
GSDMC-FL
GSDMC-N
IB:Tubulin
LDH release (%)
60
40
20
0
***
Hypoxia – + – +
Ctrl
DR6 sgRNA

q

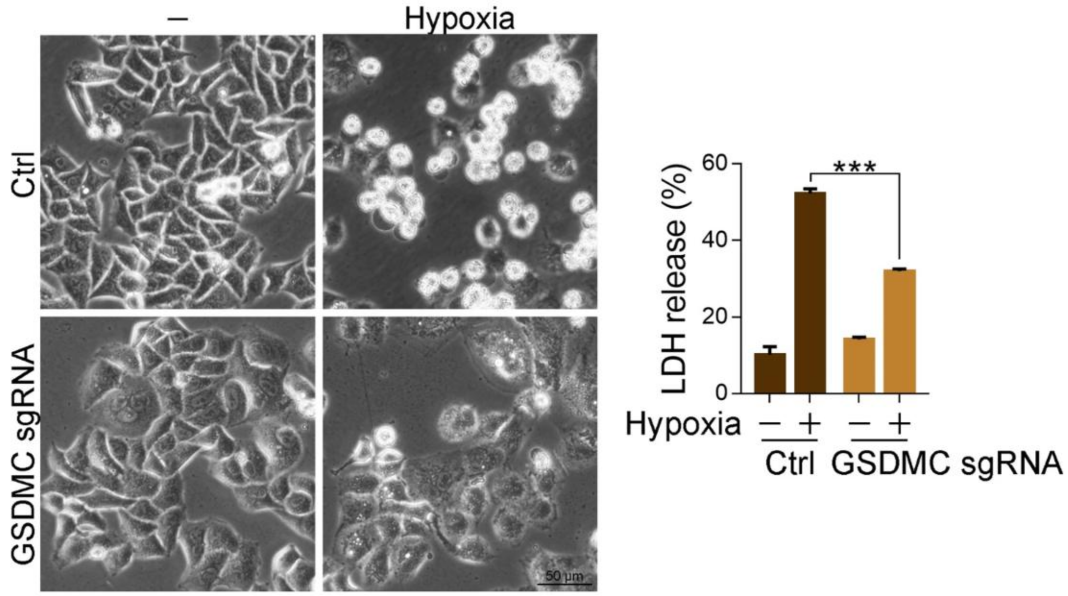

–
Hypoxia
Ctrl
GSDMC sgRNA
50 μm
LDH release (%)
60
40
20
0
***
Hypoxia
–
+
–
+
Ctrl
GSDMC sgRNA
