# Supplementary material for: The metabolite α-KG induces GSDMC-dependent pyroptosis through death receptor 6-activated caspase-8
Source: Cell Res. 2021 May 19;31(9):980–97. doi: 10.1038/s41422-021-00506-9 (PMC8410789; doi:10.1038/s41422-021-00506-9)
Supplement: Supplementary file 7 — Fig S7 [file 41422_2021_506_MOESM7_ESM.pdf]

**Supplementary information, Fig. S7.** In this figure, different normal or cancer cell lines were treated with DM-αKG (15 mM) for 24 hours to indicate cell morphology, unless specially indicated otherwise.

(**a**) Comparison of different protein expression levels in pyroptosis-sensitive and pyroptosis-insensitive cancer cell lines. Protein expression levels were measured by western blot.

(**b**) Intracellular pH was determined in the different pyroptosis-sensitive and pyroptosis-insensitive cell lines.

(**c, d**) Different cancer (c) or non-cancer (d) cell lines were cultured in medium under different pH conditions as indicated for 24 hours. The pyroptotic features are shown.

**(e)** Effects of different pH conditions on DM-αKG induced pyroptosis in HeLa and B16 cells.

(**f**) Lactic acid decreased the intracellular pH in different cancer cell lines. Cells were treated with lactic acid (20 mM) for 0.5 hours.

(**g**) Lactic acid induced the conversion of pyroptosis-insensitive cells to pyroptosis-sensitive cells. Cells were treated with lactic acid (20 mM) for 24 hours.

(**h, i, j, k**) Effect of phloretin (50 μM) or lonidamine (50 μM) on DM-αKG induced pyroptosis (h, i), intracellular pH (j) and L-2HG levels (k). HeLa cells were pretreated

with phloretin or lonidamine for 2 hours.

Tubulin was used to determine the amount of loading proteins. All data are presented as the mean±SEM of two or three independent experiments. * $p<0.05$, ** $p<0.01$, *** $p<0.001$. The data were analyzed using two-tailed Student's t-test in (f, j) or two-way ANOVA followed by the Bonferroni test in (e, h, i, k).

## Supplementary information, Figure S7

**a**

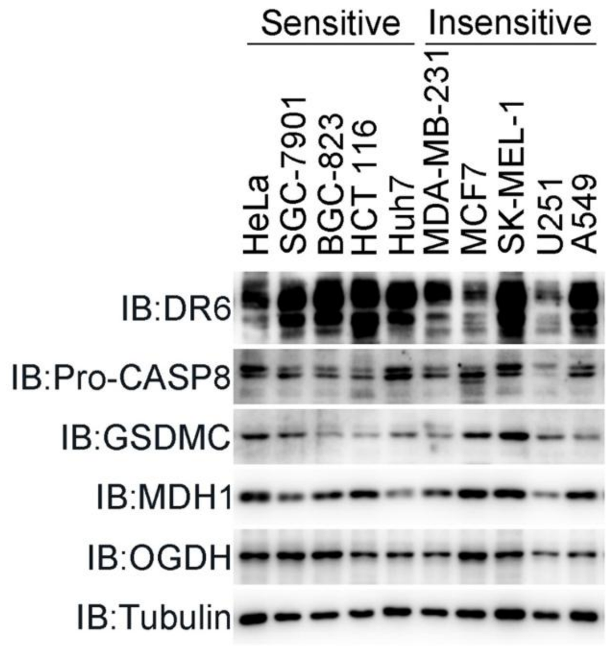


**b**

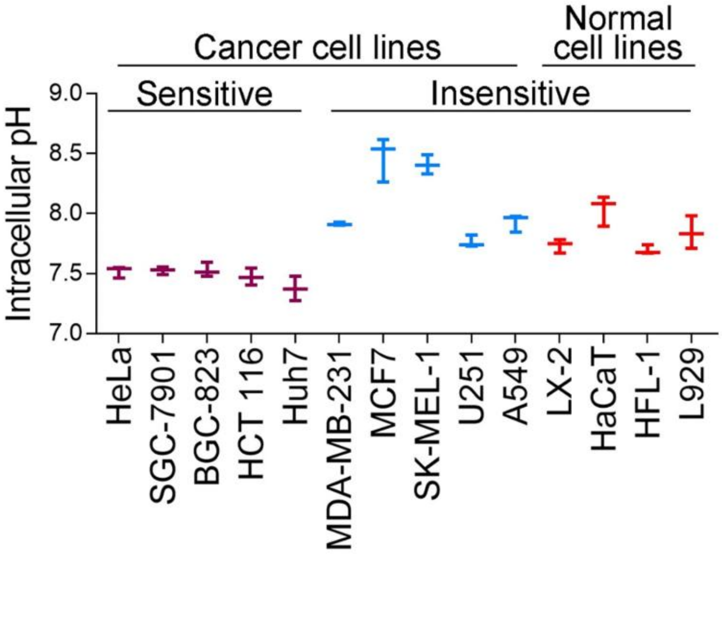

c

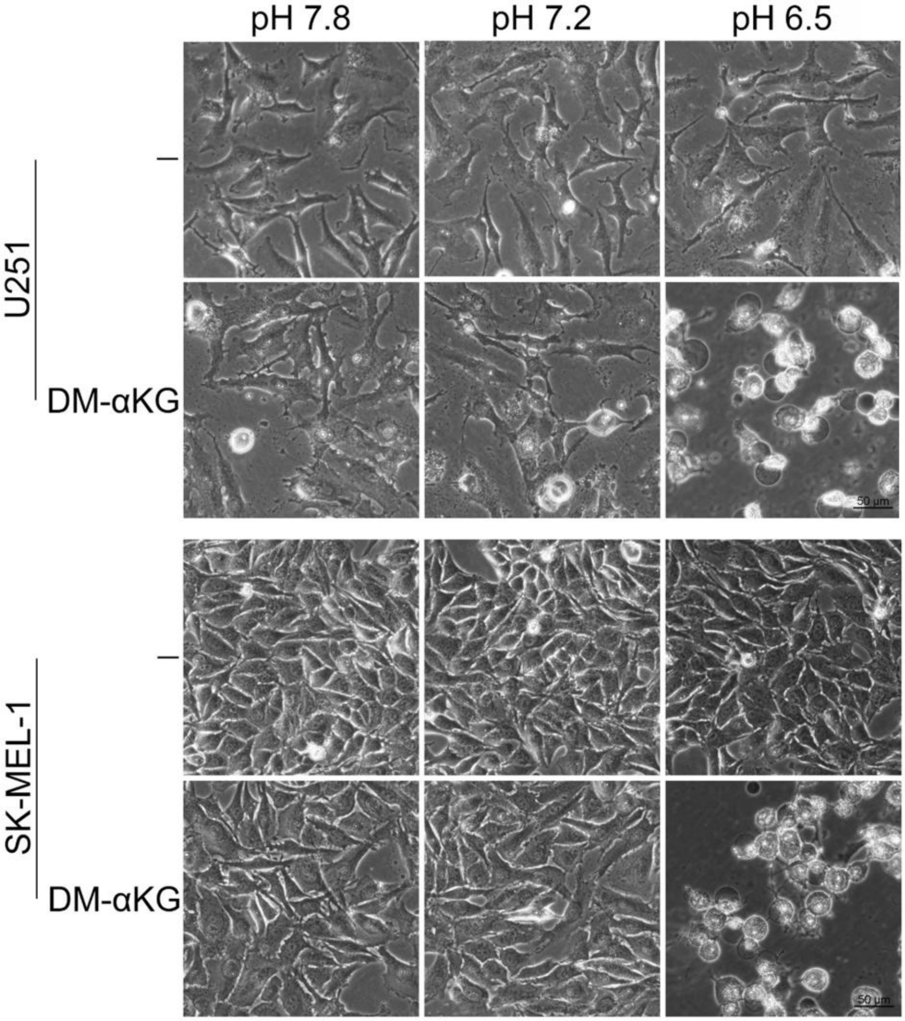


**(Continue)**

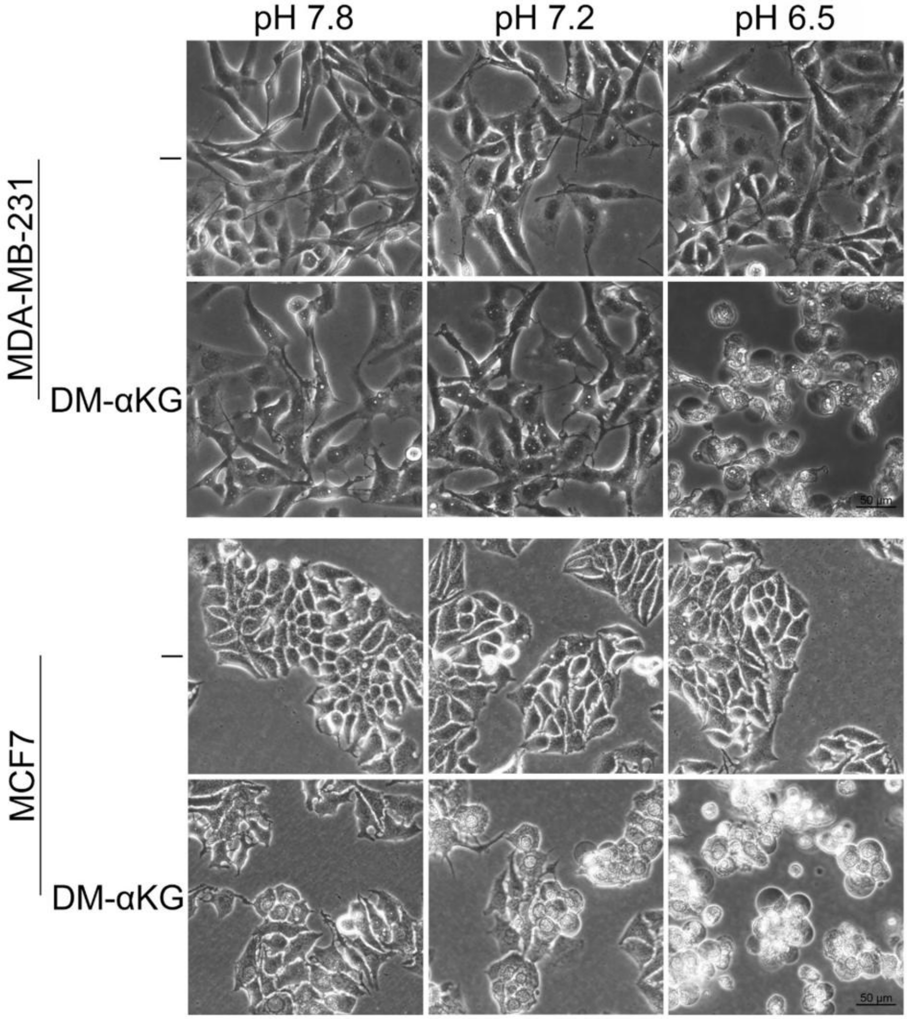

pH 7.8
pH 7.2
pH 6.5
MDA-MB-231
–
DM-αKG
MCF7
–
DM-αKG
50 µm
50 µm

**d**

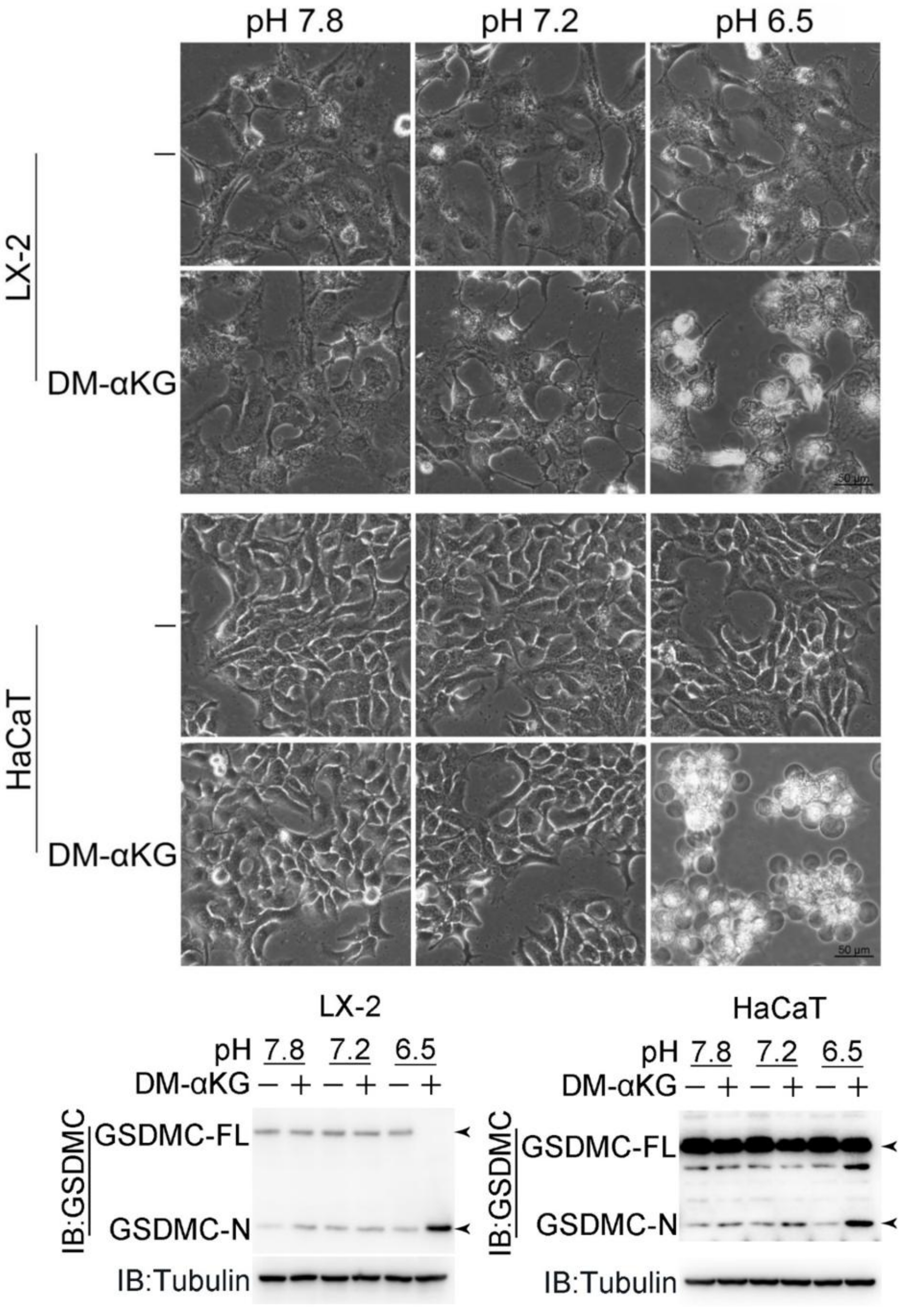
pH 7.8
pH 7.2
pH 6.5
LX-2
–
DM-αKG
HaCaT
–
DM-αKG
50 μm
50 μm
LX-2
pH 7.8 7.2 6.5
DM-αKG – + – + – +
IB:GSDMC
GSDMC-FL
GSDMC-N
IB:Tubulin
HaCaT
pH 7.8 7.2 6.5
DM-αKG – + – + – +
IB:GSDMC
GSDMC-FL
GSDMC-N
IB:Tubulin

**(Continue)**

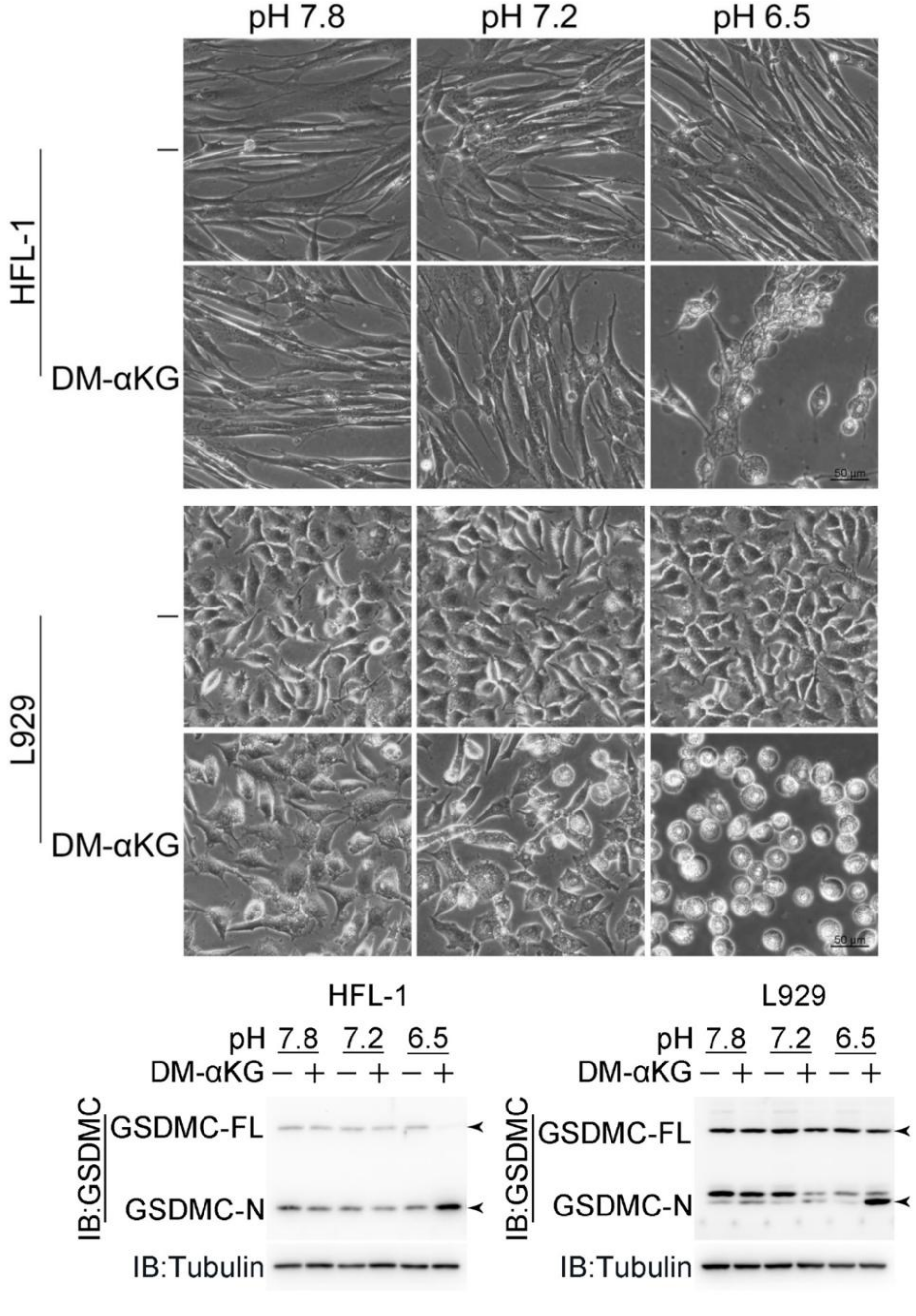
pH 7.8
pH 7.2
pH 6.5
HFL-1
DM-αKG
L929
DM-αKG
50 μm
50 μm
HFL-1
L929
pH 7.8 7.2 6.5
DM-αKG − + − + − +
IB:GSDMC
GSDMC-FL
GSDMC-N
IB:Tubulin

**e**

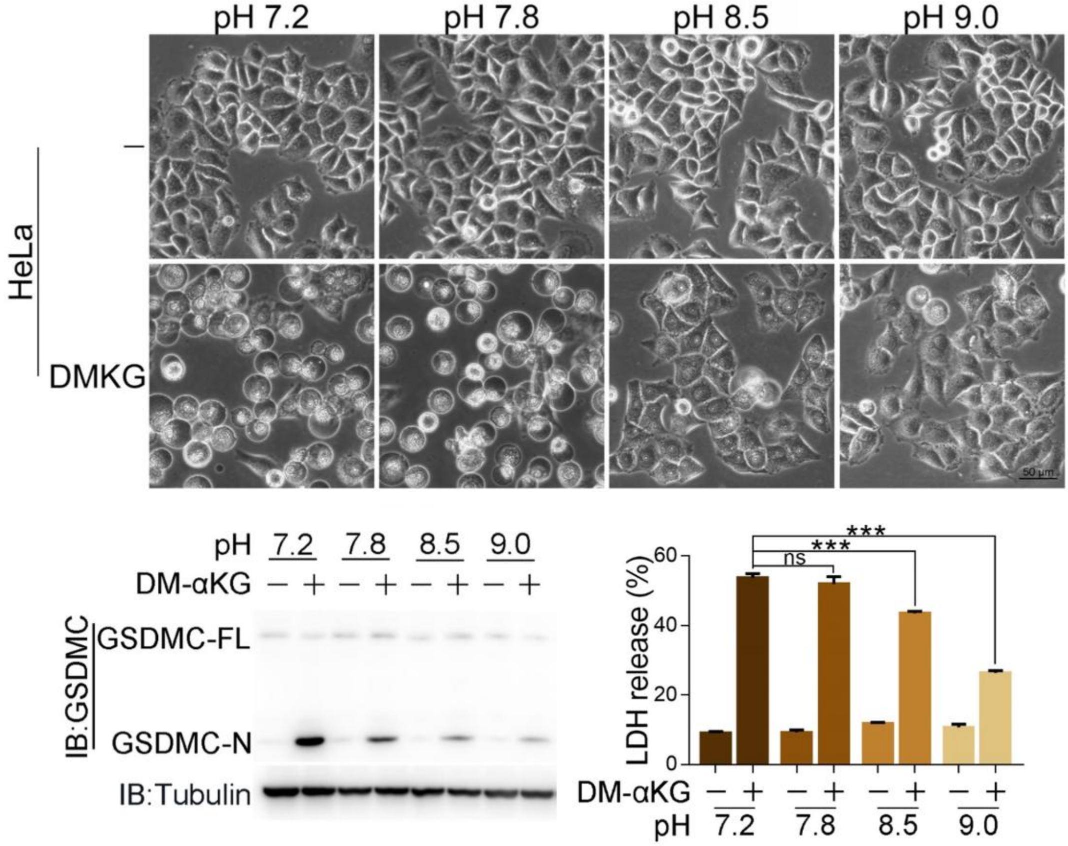
pH 7.2
pH 7.8
pH 8.5
pH 9.0
HeLa
–
DMKG
50 μm
pH 7.2 7.8 8.5 9.0
DM-αKG – + – + – + – +
IB:GSDMC
GSDMC-FL
GSDMC-N
IB:Tubulin
LDH release (%)
60
40
20
0
ns
***
***
DM-αKG – + – + – + – +
pH 7.2 7.8 8.5 9.0

**(Continue)**

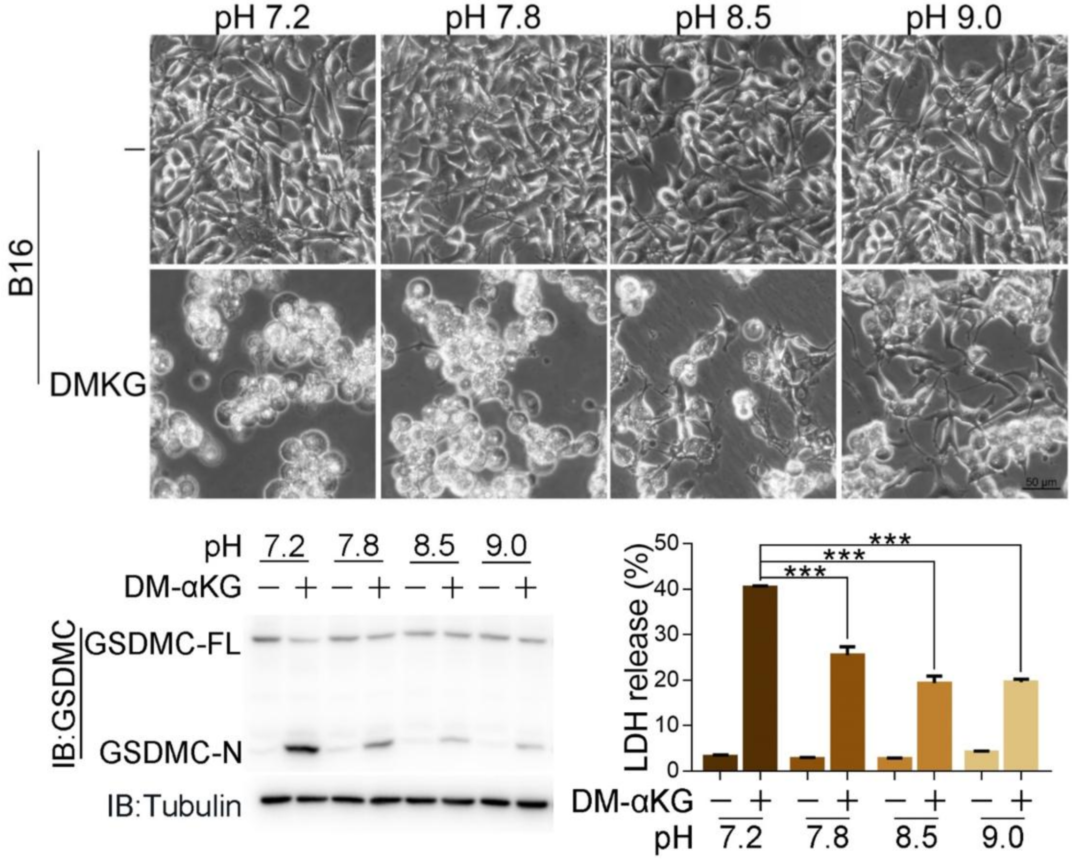

pH 7.2
pH 7.8
pH 8.5
pH 9.0
B16
–
DMKG
50 μm
pH 7.2 7.8 8.5 9.0
DM-αKG – + – + – + – +
IB:GSDMC
GSDMC-FL
GSDMC-N
IB:Tubulin
LDH release (%)
50
40
30
20
10
0
***
***
***
DM-αKG – + – + – + – +
pH 7.2 7.8 8.5 9.0

**f**

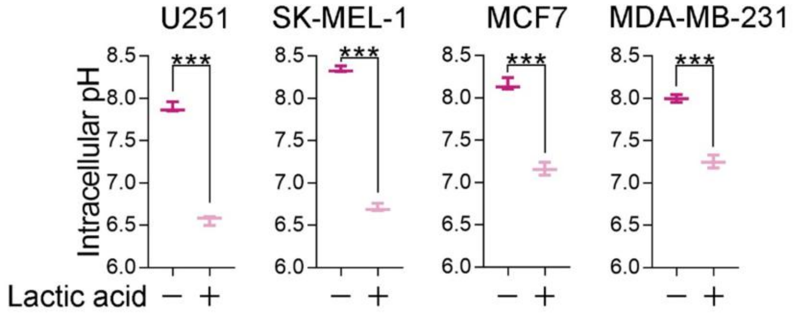
U251
SK-MEL-1
MCF7
MDA-MB-231
Intracellular pH
8.5
8.0
7.5
7.0
6.5
6.0
***
Lactic acid
−
+

**g**

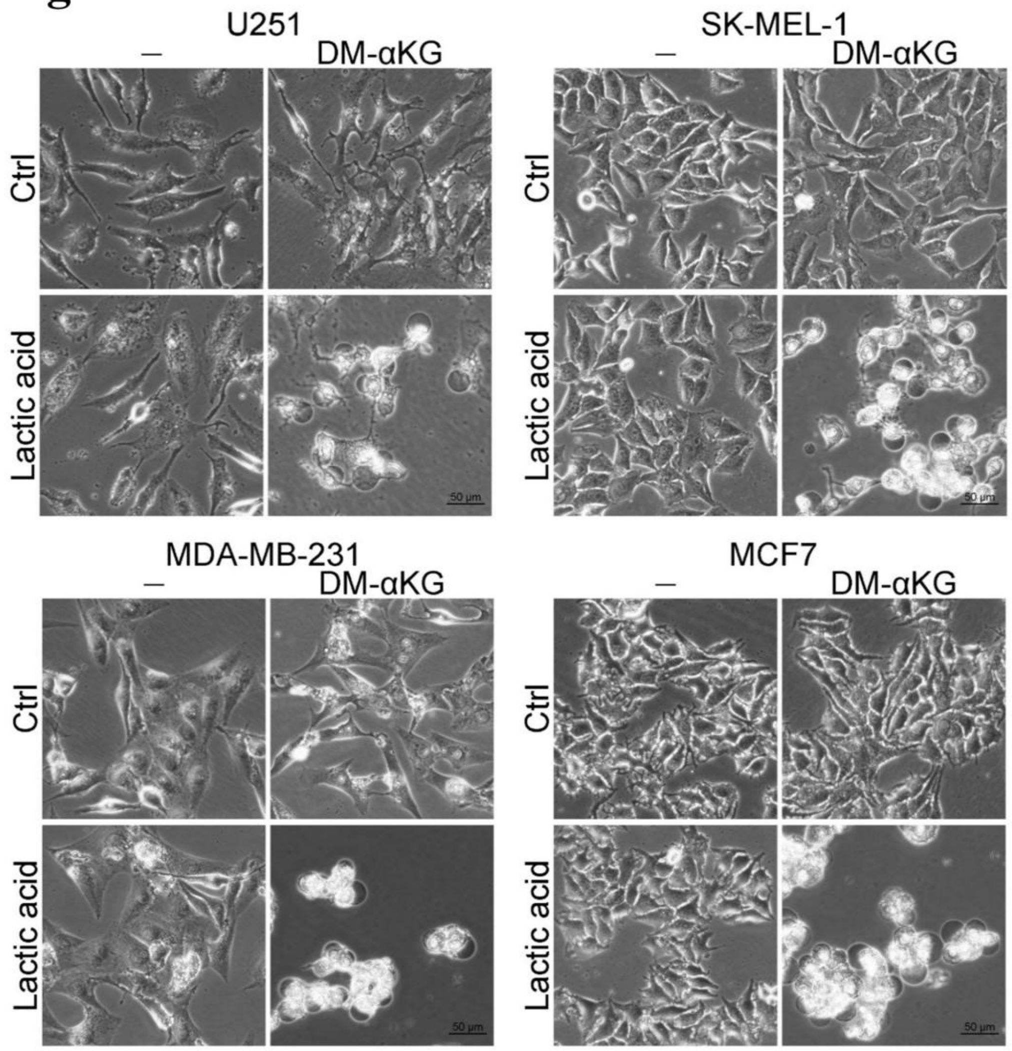
U251
SK-MEL-1
MDA-MB-231
MCF7
−
DM-αKG
Ctrl
Lactic acid
50 μm

**h**

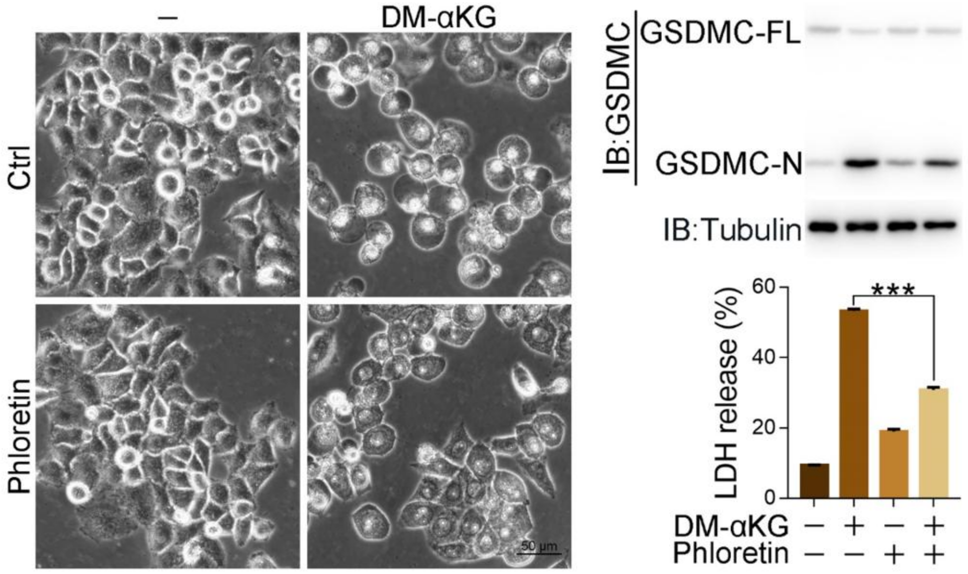

–
DM-αKG
Ctrl
Phloretin
50 μm
IB:GSDMC
GSDMC-FL
GSDMC-N
IB:Tubulin
***
LDH release (%)
0
20
40
60
DM-αKG – + – +
Phloretin – – + +


**i**

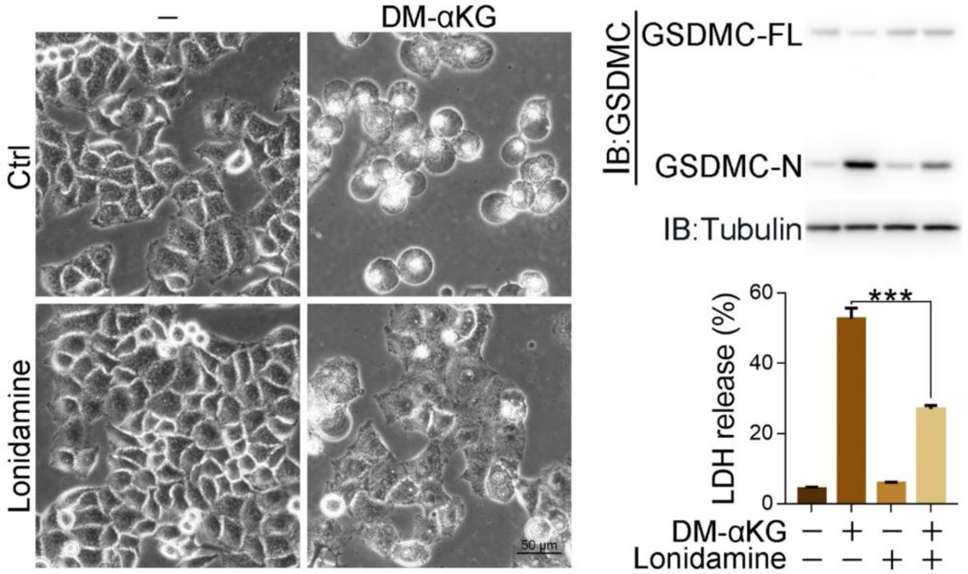

–
DM-αKG
Ctrl
Lonidamine
50 μm
IB:GSDMC
GSDMC-FL
GSDMC-N
IB:Tubulin
***
LDH release (%)
0
20
40
60
DM-αKG – + – +
Lonidamine – – + +

**j**

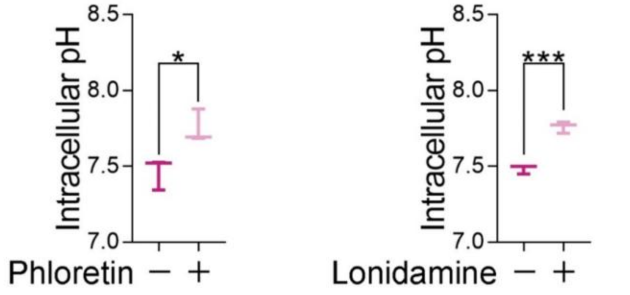
Intracellular pH
8.5
8.0
7.5
7.0
*
Phloretin − +
Intracellular pH
8.5
8.0
7.5
7.0
***
Lonidamine − +

**k**

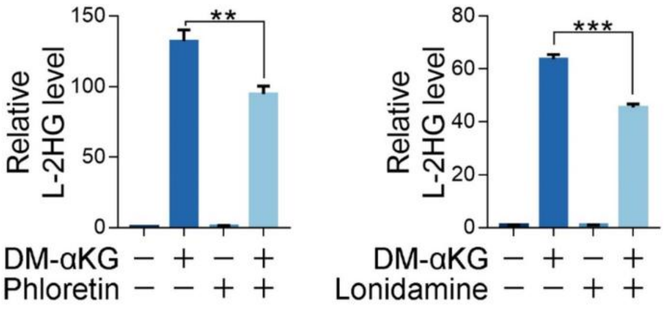
Relative L-2HG level
150
100
50
0
**
DM-αKG − + − +
Phloretin − − + +
Relative L-2HG level
80
60
40
20
0
***
DM-αKG − + − +
Lonidamine − − + +
